# Proteomic approach for understanding milder neurotoxicity of Carfilzomib against Bortezomib

**DOI:** 10.1038/s41598-018-34507-3

**Published:** 2018-11-05

**Authors:** Betul Karademir, Gulce Sari, Ayse Tarbin Jannuzzi, Sravani Musunuri, Grzegorz Wicher, Tilman Grune, Jia Mi, Husniye Hacioglu-Bay, Karin Forsberg-Nilsson, Jonas Bergquist, Tobias Jung

**Affiliations:** 1 Department of Biochemistry, School of Medicine/Genetic and Metabolic Diseases Research and Investigation Center, Marmara University, Istanbul, Turkey; 2 Department of Pharmaceutical Toxicology, Faculty of Pharmacy, Istanbul University, Istanbul, Turkey; 3 Department of Chemistry - BMC, Analytical Chemistry, Uppsala University, Uppsala, Sweden; 4 Department of Immunology, Genetics and Pathology, Neuro-Oncology, Uppsala University, Uppsala, Sweden; 5 Department of Molecular Toxicology, German Institute of Human Nutrition Potsdam-Rehbruecke (DIfE), 14558 Nuthetal, Germany; 6 German Center for Diabetes Research (DZD), 85764 Muenchen-Neuherberg, Germany; 7 German Center for Cardiovascular Research (DZHK), 10117 Berlin, Germany; 8 Department of Anatomy, School of Medicine, Marmara University, Istanbul, Turkey; 9 Department of Genetics and Bioengineering, Faculty of Engineering, Okan University, Istanbul, Turkey; 10 Medicine and Pharmacy Research Center, Binzhou Medical University, Yantai, China

## Abstract

The proteasomal system is responsible for the turnover of damaged proteins. Because of its important functions in oncogenesis, inhibiting the proteasomal system is a promising therapeutic approach for cancer treatment. Bortezomib (BTZ) is the first proteasome inhibitor approved by FDA for clinical applications. However neuropathic side effects are dose limiting for BTZ as many other chemotherapeutic agents. Therefore second-generation proteasome inhibitors have been developed including carfilzomib (CFZ). Aim of the present work was investigating the mechanisms of peripheral neuropathy triggered by the proteasome inhibitor BTZ and comparing the pathways affected by BTZ and CFZ, respectively. Neural stem cells, isolated from the cortex of E14 mouse embryos, were treated with BTZ and CFZ and mass spectrometry was used to compare the global protein pool of treated cells. BTZ was shown to cause more severe cytoskeletal damage, which is crucial in neural cell integrity. Excessive protein carbonylation and actin filament destabilization were also detected following BTZ treatment that was lower following CFZ treatment. Our data on cytoskeletal proteins, chaperone system, and protein oxidation may explain the milder neurotoxic effects of CFZ in clinical applications.

## Introduction

The proteasomal system regulates the cellular protein pool and proteolytic activities are localized on three different β-subunits: β1 (trypsin-like), β2 (peptidyl-glutamyl peptide-hydrolysing) and β5 (chymotrypsin-like). The main activity of the proteasome is localized on the β5 subunit. There are different “types” of the proteasome; 20S (ATP-independent proteolysis of unfolded proteins), 26S (ATP-dependent degradation of functional proteins), immunoproteasome and hybrid proteasome^1^.

Proteasome inhibitors are used in cancer therapy, because inhibition of the proteasome results in enhanced susceptibility of cancer cells to oxidative stress/chemotherapy^2,3^. Bortezomib (N-pyrazinecarbonyl-L-phenylalanine-L-leucine boronic acid, PS-341, VELCADE®, Millenium Pharmaceuticals Inc.) is the first generation of proteasome inhibitors approved by FDA in 2003 for treatment of multiple myeloma and in 2006 for mantle cell lymphoma^4–7^. Bortezomib (BTZ) inhibits proteasome mainly via binding the β5 subunit^1^. Carfilzomib (PR-171, KYPROLIS®, Onyx Pharmaceuticals Inc.), which inhibits the proteasome irreversibly, is an epoxy ketone proteasome inhibitor. Carfilzomib (CFZ) also inhibits the activity of β5 subunit. This second-generation proteasome inhibitor was approved by FDA for relapsed or refractory multiple myeloma treatment in 2012.

Following the approval by FDA, BTZ has been used in the clinic for a large number of hematologic cancer patients as adjuvant therapy^8^. In addition to its anticancer effect, BTZ has a serious side effect mainly on neural cells, which has been called BTZ induced peripheral neuropathy (BIPN). Most common symptoms of BIPN are neuropathic pain and paresthesia and these neuropathies not only distract motor ability but also lead to sensory symptoms^5^. Data explaining the exact mechanism of BIPN are limited, but there are several hypotheses suggesting mitochondria dependent apoptosis^9^, failure of calcium homeostasis^10^, and failure in the regulation of neurotrophins^11^.

The main reason for the development of second-generation proteasome inhibitors was to decrease these peripheral neuropathy side effects^12^. CFZ is currently in phase III studies and the combination of BTZ + dexamethasone was compared with CFZ + dexamethasone. The peripheral neuropathy effects of the BTZ combination were approximately 5 fold higher than CFZ combination^13^. A very recent study, showed the mechanisms of milder cardiotoxic effects of CFZ compared to BTZ in the *Drosophila* model^14^.

In this study we used neural stem cells (NSCs) as a model to minimize the cell specific phenotype and response differences. Our aim was to compare the toxicity of BTZ and CFZ on these cells. In line with this purpose, we treated NSCs isolated from E14 mouse embryos with BTZ and CFZ and analyzed the neural proteome for the prospective targets of neuropathy using nanoLC-MS/MS. In accordance with the proteomic data, we performed further expression analysis of cytoskeleton members and heat shock proteins (HSPs) as well as the interaction between HSP70 and actin monomers. Our study confirmed lower toxicity of CFZ when compared to BTZ. Furthermore, our data point to protein damage and related stress response system (heat shock proteins) as the most significant reason of higher BTZ toxicity. On the other hand, non-neuronal cell lines, which are cardiomyoblasts and embryo fibroblasts were used to compare if the effects of proteasome inhibitors are distinguishable on the neuronal and non-neuronal cell lines.

## Results

### BTZ and CFZ inhibit proteasome activity equally in NSCs

**Figure 1:**
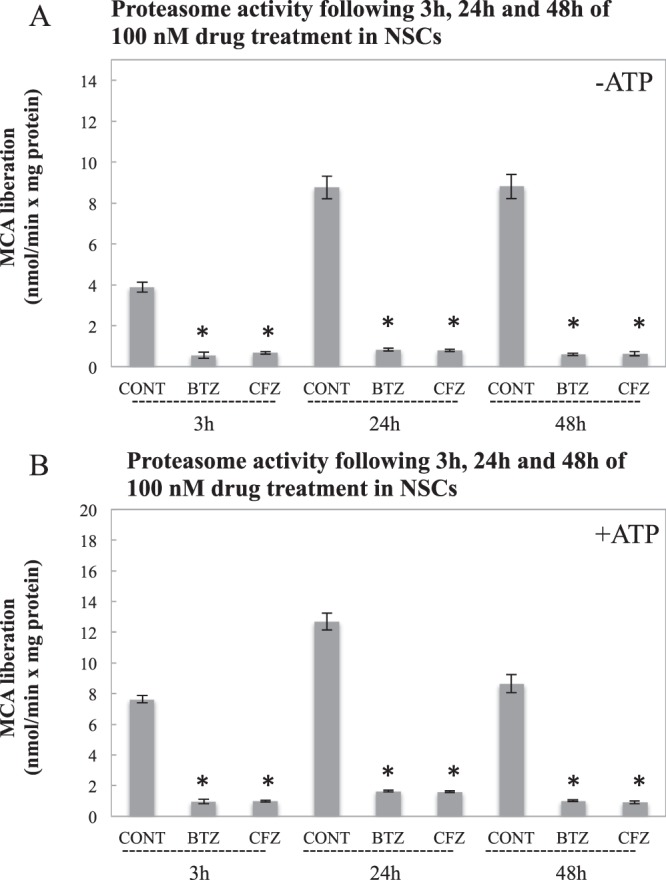
Effects on proteasome activity in mouse NSCs following 3 h, 24 h and 48 h of 100 nM BTZ and CFZ treatments. Data denote mean ± S.D. *p < 0.05 vs. CONT (n = 3). Results were evaluated with ANOVA test followed by multiple comparison analysis. (**A**) Represents proteasome activity without ATP addition; (**B**) represents proteasome activity with ATP addition into the reaction mixtures.

Our results showed that both BTZ and CFZ significantly inhibited proteasome activity following 3 h, 24 h and 48 h at 100 nM concentrations in NSCs (p < 0.05) (Fig. 1). This inhibition was comparable to H9c2 myofibroblasts (p < 0.05) while embryo fibroblasts were more resistant to the inhibitors (p > 0.05 in 3 h and 48 h time points) at 100 nM concentrations (Supplementary Figure 6).

### Functional classification of differentially expressed proteins according to NanoLC-MS/MS analysis

We have used nanoLC-MS/MS method to investigate the effect of BTZ or CFZ treatment on global protein pool 3 h, 24 h and 48 h following each treatment. Since NSCs start to differentiate after 48–72 hours in the absence of growth factors^15^, we mostly focused on the 24 h treatment groups for further analyses. The proteomic data for 24 h treatments of BTZ and CFZ are given in Supplementary Tables and also in Supplementary Figure 1 and 2. In BTZ group, 92 proteins were differentially expressed compared to CONT and CFZ groups. There were 45 differentially expressed proteins in CFZ compared to CONT and BTZ groups (Supplementary Figure 2). To identify biological processes and cellular pathways dependent on BTZ or CFZ treatment, the obtained data was analyzed with STRING database and software (v.10.0) in terms of gene ontology (GO) and KEGG pathways^16^. Following 24 h treatment, observed biological processes were basically RNA production and processing, translation and proteasomal system related protein turnover in CONT group. BTZ treatment shifted the cellular processes toward protein processing in endoplasmic reticulum (ER), cellular component assembly and macromolecular/protein complex organization. On the other hand, KEGG analysis showed that only CFZ treatment affected oxidative phosphorylation related proteins. Both BTZ and CFZ treatments affected molecules related to myelin sheath formation. Notably, according to KEGG pathway analysis, the common affected molecules were related with neurodegenerative diseases. Table 1 shows GO and KEGG pathway classification.

**Table 1:** Proteins detected in NanoLC-MS/MS experiments following 24 h treatment of BTZ and CFZ and analyzed by STRING database and gene ontology (GO) and KEGG pathways software (v.10.0).

Groups	ID and Description (GO)	p value	ID and Description (KEGG)	p value
Only CONT Group	GO:0006412-Translation	3.4e-08		
	GO:0006413-Translational Initiation	0.000282		
	GO:0006396-RNA Processing	2.79e-11		
	GO:0008380-RNA splicing	3.71e-10		
	GO:0010498-Proteasomal protein catabolic process	0.000204		
	GO:0043161 proteasome-mediated ubiquitin-dependent protein catabolic process	0.00058		
Only BTZ Group	GO:0016043-Cellular component organization	0.0187	04141-Protein processing in endoplasmic reticulum	0.0136
	GO:0043933-Macromolecular complex subunit organization	0.0208		
	GO:0071822-Protein complex subunit organization	0.0208		
	GO:0022607-Cellular component assembly	0.0218		
Only CFZ Group	GO:0008152-Metabolic process	0.00497	00190-Oxidative phosphorylation	0.00143
Both BTZ and CFZ Groups	GO:0043209-Myelin sheath	0.000162	05012-Parkinson’s disease	0.00068
			05010-Alzheimer’s disease	9.52e-05
			05016-Huntington’s disease	0.00105

There are many published research articles and reviews regarding the toxic effect of BTZ and they mainly focus on cytoskeletal damage and mitotoxicity^12,17,18^. On the other hand, the proteasomal system is closely related with the chaperone system and oxidative protein modifications. Based on these relations, we selected the proteins and showed their time- and exposure-dependent changes in Table 2. Proteins related to correct folding process HSP60, HSP70, HSP90, and protein disulfide-isomerase A3/A6 were differently expressed following BTZ and CFZ treatments. Secondly, antioxidant system members such as superoxide dismutase and catalase were affected differently following 24 h of BTZ and 3 h of CFZ treatment. In addition to these proteins, ubiquitin-like modifier-activating enzyme 1 and microtubule-associated protein were regulated following 24 h treatment; caprin-1, lamin B1 and 14-3-3 protein epsilon were regulated following 48 h treatment of BTZ and CFZ (Table 2).

**Table 2:** Regulated proteins in BTZ and CFZ groups at different time points (n.s. nonsignificant).

Protein Name	Regulation in BTZ group compared to CONT group	p value (BTZ vs CONT)	Regulation in CFZ group compared to CONT group	p value (CFZ vs CONT)
**Regulated Proteins Following 24 h**				
Stress-70 protein	Up regulated	0.003	n.s.	—
Superoxide dismutase [Mn]	Up regulated	0.017	n.s.	—
Protein disulfide-isomerase A3	Up regulated	0.045	n.s.	—
Heat shock protein HSP 90-alpha	Up regulated	0.045	Up regulated	0.001
Ubiquitin-like modifier-activating enzyme 1	Down regulated	0.014	Down regulated	0.013
Microtubule-associated protein	Down regulated	0.012	n.s.	—
**Regulated Proteins Following 3 h**				
Catalase	n.s.	—	Up regulated	0.031
**Regulated Proteins Following 48 h**				
Caprin-1	Down regulated	0.034	n.s.	—
60 kDa heat shock protein (mt)	n.s.	—	Up regulated	0.031
Lamin B1	n.s.	—	Down regulated	0.0001
14-3-3 protein epsilon	Up regulated	0.022	n.s.	—
Protein disulfide-isomerase A6	Up regulated	0.043	n.s.	—

Analyzing the data obtained in NanoLC-MS/MS, we have decided to focus on the expressions of cytoskeletal proteins, and chaperone system proteins in detail. Also protein carbonylation and ubiquitination were tested as a marker of protein oxidation and degradation. Results of these mentioned detailed analyses are summarized in Table 3. On the other hand, mitotoxicity related data is being tested in the context of another study carried by our group.

**Table 3:** Functional classification and tested proteins according to data of NanoLC-MS/MS analysis following BTZ and CFZ treatments.

Classification	Tested Proteins	Time Point	Result (BTZ vs CONT)	Result (CFZ vs CONT)
Cytoskeletal proteins	Nestin	24 h	Decreased	Decreased
	Vimentin	24 h	Increased	Decreased
	Actin Related Protein-2	3 h	Increased	Increased
	Transgelin-2	3 h	Not changed	Not changed
	Coronin 1 C	3 h	Not changed	Not changed
	β-actin	3 h	Decreased	Decreased
	β-tubulin	24 h	Increased	Increased
Chaperone System	HSP32	24 h	Increased	Not changed
	HSP47	24 h	Increased	Not changed
	HSP70	24 h	Increased	Not changed
	GRP78	24 h	Increased	Increased
	GRP94	24 h	Increased	Increased
Protein Oxidation	Ubiquitinated proteins	24 h	Increased	Increased
	Carbonylated proteins	24 h	Increased	Increased

### BTZ and CFZ treatments affect the expressions of cytoskeletal proteins

**Figure 2:**
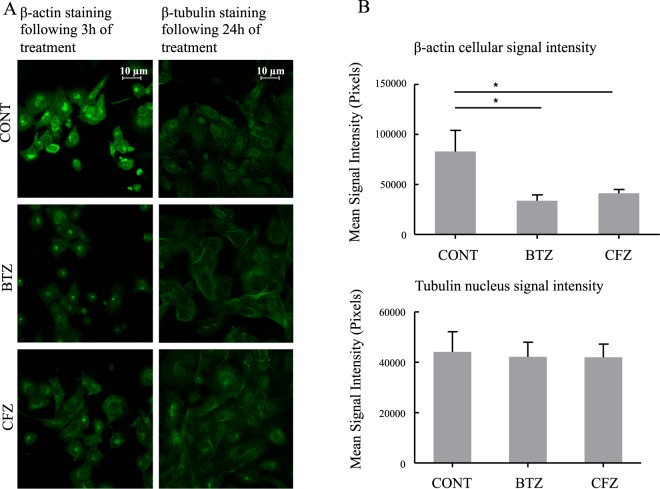
Effects on β-actin- and β-tubulin distribution in mouse NSCs following 3 h of BTZ and CFZ treatments for β-actin and 24 of BTZ and CFZ treatments for β-tubulin. Cells were treated and prepared for confocal microscopy as described in the materials and methods. (**A**) Selected images of confocal microscopy. (**B**) Mean signal intensities calculated by using Image-J software. Data denote mean ± % S.D. *p < 0.05 vs. CONT group (n = 3).

Actin filaments following 3 h of BTZ and CFZ treatments and microtubule stability following 24 h of BTZ and CFZ treatments were visualized by confocal microscopy. As shown in Fig. 2, BTZ treatment caused decreased actin filament signal intensity. In addition, cells changed their shapes and cells exhibited shrunken morphology. Following treatment of BTZ and CFZ, β-tubulins around the nucleus were no longer visible (Fig. 2).

**Figure 3:**
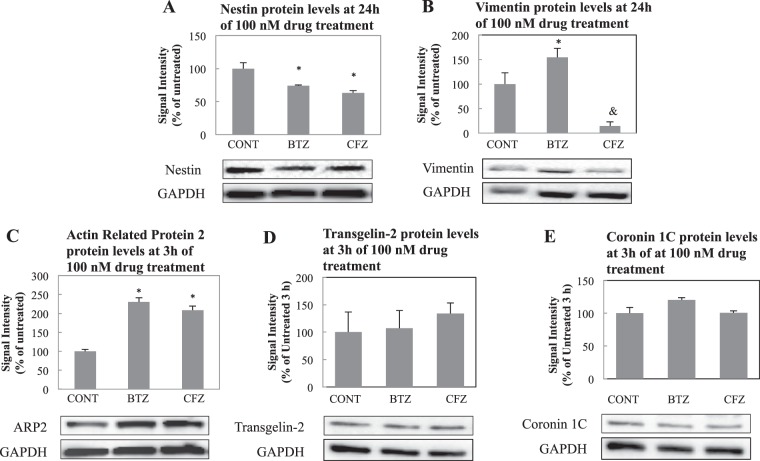
Effects on cytoskeletal proteins of mouse NSCs following different time points of 100 nM BTZ and CFZ treatments. (**A**,**B**) Nestin and Vimentin expressions following 24 h of treatments. (**C**–**E**). Actin related protein-2, transgelin-2, and coronin 1 C expressions following 3 h of treatment. CONT was set to 100%. Percentage of all lanes was calculated as proportioning the lane density to CONT. Blot is cropped to improve the clarity. Full-length blots are included in the Supplementary Data. Data denote mean ± % S.D. *p < 0.05 vs. CONT group, ^&^p < 0.05 vs. BTZ group (n = 3).

In view of the NanoLC-MS/MS data, cytoskeletal proteins nestin and vimentin were analyzed by immunoblotting following 24 h, actin related protein-2, and transgelin-2 and coronin 1 C were analyzed following 3 h of treatments. These proteins were also tested in H9c2 cardiomyoblast cells and BALB/3T3 embryo fibroblasts. Nestin is a class VI and vimentin is a class III intermediate filament protein. They are also described as NSC markers. Nestin expression decreased following both BTZ and CFZ treatments compared to CONT in NSCs (Fig. 3A) and decreased only following BTZ treatment in only BALB/3T3 cells (Supplementary Figure 7). Vimentin expression increased following BTZ treatment and decreased following CFZ treatment compared to CONT in NSCs (Fig. 3B) and decreased following both BTZ and CFZ treatments compared to CONT in H9c2 cardiomyoblasts (Supplementary Figure 7). Results for nestin expression following 3 h and 48 h of BTZ and CFZ treatments were also similar to 24 h in NSCs (data shown in Supplementary Figure 3). Actin related protein-2 increased in both BTZ and CFZ treated groups in NSCs and only in H9c2 cardiomyoblasts and transgelin-2 increased nonsignificantly only in CFZ treated group compared to CONT (Fig. 3C,D) (Supplementary Figure 8). There was no significant change in another F-actin cross-linking protein, coronin 1 C (Coronin 3) (Fig. 3E). Coronin 1 C changed in both H9c2 and BALB/3T3 cells (Supplementary Figure 8). Results for actin related protein-2, transgelin-2 and coronin-1C following 24 h and 48 h of BTZ and CFZ treatments were different than those of 3 h results in NSCs (Supplementary Figure 4).

### Heat shock protein expressions change differently following BTZ and CFZ treatments

**Figure 4:**
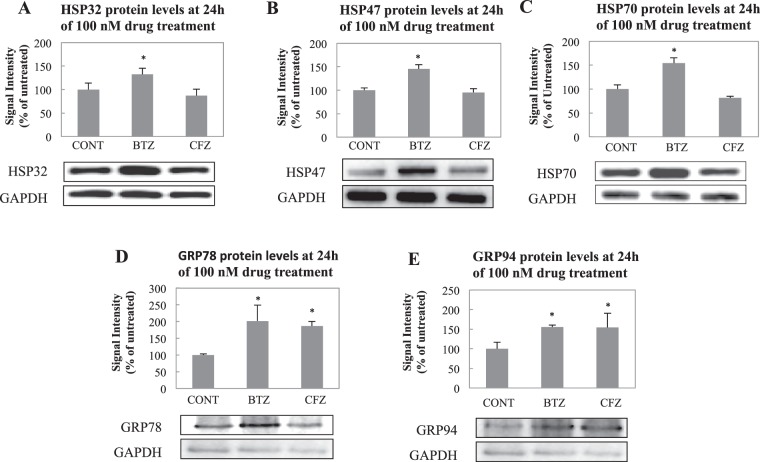
Effect on chaperone system in mouse NSCs following 24 h of 100 nM BTZ and CFZ treatments. Representative immunoblots of (**A**). HSP32, (**B**) HSP47, (**C**) HSP70, (**D**) GRP78 and (**E**) GRP94 expression levels. CONT 24 h was set as 100%. Percentage of all lanes was calculated by proportioning the lane density to CONT. Blot is cropped to improve the clarity. Full-length blots are included in the Supplementary Data. Data denote mean ± % S.D. *p < 0.05 vs. CONT group (n = 3).

According to the results following 24 h of treatment, BTZ caused increase in all 5 proteins (Fig. 4), whereas CFZ did not cause any significant change in HSP32, HSP47 and HSP70 (Fig. 4A–C) but caused an increase in GRP78 and GRP94 (Fig. 4D,E, p < 0.05). All 5 proteins showed significant change in H2c9 cardiomyoblasts (Supplementary Figure 9 and 10, p < 0.05) and only GRP94 showed significant change in BALB/3T3 fibroblasts (Supplementary Figure 9 and 10, p < 0.05).

CFZ treatment caused elevation in the expression of HSP32, HSP40, HSP47, HSP60 and HSP70 following 3 h treatment in NSCs. It seems that the effect of CFZ on HSPs was transient because following 24 h and 48 h of treatment, expressions of HSPs were down-regulated and reached to expression levels of controls or even lower. Both BTZ and CFZ caused approximately 2.5–3.0 fold increase in HSP60 expression and 2-fold decrease in HSP90 expression following 48 h of treatments. Expressions of HSP27 decreased following 3 h treatments of both BTZ and CFZ and increased following 48 h treatments of both BTZ and CFZ (Supplementary Figure 5).

### Both BTZ and CFZ trigger the interaction of HSP70 and β-actin

**Figure 5:**
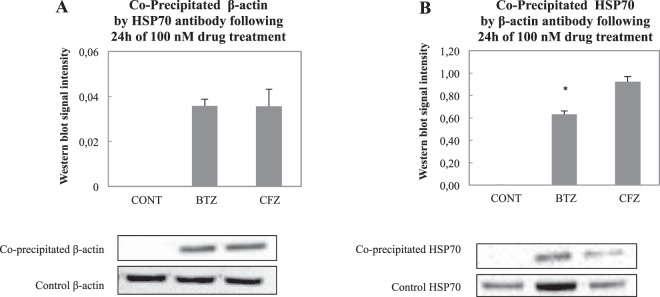
β-actin and HSP70 interaction analysis following 100 nM BTZ and CFZ treatments for 24 h. The β-actin and HSP70 interaction analysis was performed with co-immunoprecipitation methods as described. The precipitated band density was divided by cell lysate band density without precipitation, for standardization. Blot is cropped to improve the clarity. Full-length blots are included in the Supplementary Data. Data denote mean ± % S.D. *p = 0.037 vs. CONT group (n = 3).

HSP70 and β-actin interaction following BTZ and CFZ treatments were analyzed via co-immunoprecipitation following 24 h of treatment. To standardize our results, we also performed immunoblotting for the same volume of control samples after 24 h treatment. Densities of co-precipitation lanes were divided by the density of control lanes. The precipitated β-actin and HSP70 proteins were shown following precipitation in both directions at around 100 kDa. Bands and corresponding graphs are shown in Fig. 5.

According to our results, the ratio of precipitated β-actin to control was same in BTZ and CFZ treated groups after 24 h. But, the ratio of precipitated HSP70 to control was smaller in BTZ treated group (Fig. 5B). Since HSP70 expression increases dramatically following 24 h treatment of BTZ, the density of precipitated band was lower than CFZ treated group.

### BTZ leads to higher protein carbonylation and ubiquitinated-protein accumulation than CFZ

**Figure 6:**
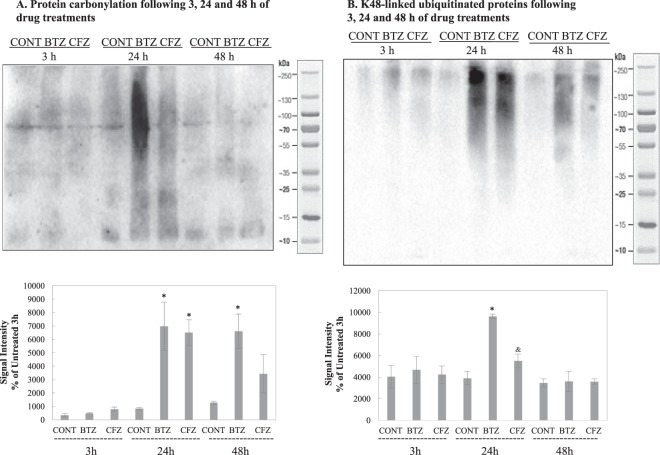
Impact of BTZ and CFZ on protein carbonylation and accumulation of K48-linked ubiquitinated proteins. Cells were treated with BTZ and CFZ; cell lysates were prepared and analyzed via immunoblotting as described in methods. Blots represent one from three replicates (n = 3). (**A**) shows protein carbonyls following 3 h, 24 h and 48 h treatment; (**B**) shows K48-linked ubiquitinated-proteins following 3 h, 24 h and 48 h treatment. Blot is cropped to improve the clarity. Full-length blots are included in the Supplementary Data. Data denote mean ± % S.D. *p < 0.05 vs. CONT group, ^&^p < 0.05 vs. BTZ group.

Protein carbonylation was detected following DNPH treatment via immunoblotting using anti-DNPH antibody. K48 linked ubiquitinated proteins were also detected by immunoblotting using anti-ubiquitin antibody. As shown in Fig. 6A,B, BTZ caused higher protein carbonylation and ubiquitinated-protein accumulation than CFZ following 24 h treatments. In both groups, protein carbonylation decreased after 48 h treatment, and no significant increase in protein carbonyls following 3 h of treatment was detected. BALB/3T3 cells showed same profile with NSCs and BTZ caused higher protein carbonylation and ubiquitinated-protein accumulation than CFZ following 24 h treatments. Also ubiquitinated proteins were still high in BTZ treated group following 48 h (Supplementary Figure 11). H9c2 cells showed high protein carbonylation and ubiquitinated-protein accumulation following 24 h treatments of both BTZ and CFZ (Supplementary Figure 12). This data is supporting the reported cardiotoxic side effects of CFZ in the clinics.

## Discussion

In this study, we aimed to provide a mechanistic highlight to a very critical problem in the clinic regarding proteasome inhibitors. Our aim was to compare the side effects of the first generation proteasome inhibitor BTZ with the second-generation proteasome inhibitor CFZ at the proteome level. BTZ is actively used in the clinic especially in the treatment of hematologic cancers. It was found to be very effective for many years and in millions of patients. But in recent years, a big discussion arose among clinicians about dose reduction and also abandoning it because of its side effects. As a solution to this crucial problem, second-generation proteasome inhibitors have been developed. Among others CFZ is the first clinically approved one^13^. CFZ is epoxy ketone while BFZ is in boronic acid form. This structural difference is believed to decrease the side effects by increasing the specificity and decreasing the damage on non-target proteins^19^.

Since the major side effect of BTZ is neuropathy, we used E14 mouse embryo cortex-derived NSCs. Following the significant inhibition of proteasome activity by both BTZ and CFZ at 3 h, 24 h and 48 h time points, we performed proteomics analyses to categorize the differentially expressed proteins. According to obtained data, we made a functional classification such as cytoskeletal proteins, chaperone system and protein oxidation as the main differentially affected processes (Table 3). Cardiomyoblasts and embryo fibroblasts were used as nonneuronal cells to compare if the effects of BTZ and CFZ are distinguishable on the neuronal and non-neuronal cell lines.

The shapes of the neural cells, performing transport, inter- and intra-cellular communication are key elements for neural cell survival and function. Microtubules are very important in achieving proper transport and communication. Microtubules are strongly related to neuropathies and there are drugs targeting microtubules^20^. Staff *et al*.^21^ indicated that microtubule damage is related to BIPN. According to their dorsal root ganglion (DRG) *in vitro* cell culture system, BTZ caused increased tubulin polymerization that caused axonopathy. Poruchynsky *et al*.^22^ applied different proteasome inhibitors as lactacystin, BTZ and CFZ and showed that proteasomal inhibition caused increased tubulin stabilization and elevated microtubule associated protein expression. They did not see any increase in microtubule polymerization following application of BTZ and CFZ to isolated microtubules^22^. Similar to reported data^21,22^, signal intensity was high following β-tubulin staining in our drug treated NSCs (Fig. 2). This may be caused by increased microtubule polymerization and stabilization. Interestingly, drug treatment caused withered microtubule signal around cell nucleus (Fig. 2B). Proteasomal inhibition caused not only increased microtubule polymerization but also changed cellular arrangement of microtubules. In addition to microtubule stabilization and polymerization, actin filaments are crucial structures for cell integrity and BTZ and CFZ decreased the actin density in the cells, which caused the cells to shrink. BTZ related cell shrinkage was higher compared to CFZ suggesting higher toxicity (Fig. 2).

Lamin B1, one of the major constituents of nuclear lamina^23^, was down regulated following CFZ treatment (Table 2) whereas BTZ did not affect this protein at all. This result is in agreement with the study of Yoon *et al*.^24^, which showed that lamin B plays a role in axon maintenance via its role in mitochondrial function. CFZ also affected mitochondrial oxidative phosphorylation related proteins according to the STRING database, gene ontology and KEGG pathways analysis (Table 1), thus lamin B1 decrease may be related with these mitochondrial changes.

Nestin is a class VI intermediate filament that is highly expressed in precursor neural cells^15^ and crucial in cell survival and proliferation^25^. In our case, both BTZ and CFZ reduced the expression of nestin following 24 h treatment (Fig. 3A). On the other hand, vimentin as a class III intermediate filament is related to cytoplasmic stiffness^26^, was affected differently by BTZ and CFZ (Fig. 3B). Vimentin has different roles in composing cellular shape and adhesion, and cellular localization of organelles and vacuoles^27^. Increased accumulation of vimentin was reported in giant axonal neuropathy (GAN), which is caused by mutations of GAN gene encoding for a member of E3 ubiquitin ligases. Vimentin accumulated following the mutation of GAN because of not being degraded by proteasomal system^28^. Similar to GAN mutation, proteasome inhibition may also cause vimentin accumulation in NSCs, but higher vimentin accumulation in BTZ treated group compared to CFZ treated group (Fig. 3B) needs further explanation. In a very recent study, vimentin increase was shown as a marker of neuronal degeneration^29^. On the other hand, overexpression of vimentin is accepted to be an inflammation marker^30,31^. Since BTZ is accepted to be more toxic than CFZ, the increase in vimentin expression may be an indicator for higher neuronal damage. In addition, immunoprecipitation experiments showed that 14-3-3 proteins interact with vimentin^30,31^. In our study, 14-3-3 epsilon expression was also detected in BTZ treated group following 48 h treatment (Table 2).

After showing actin filament changes following BTZ and CFZ treatments, western blotting was performed for candidate actin-filament related proteins. Actin related protein 2 (Arp2) expression was found to be almost 2-fold increased following BTZ and CFZ treatment for 3 h (Fig. 3C). Contrary to its acute increase following 3 h drug treatment, Arp2 expression decreased following 24 h and 48 h of BTZ treatment. Surprisingly a similar decrease was not detected following 24 h and 48 h of CFZ treatment (Supplementary Figure 4). Arp2/3 complex has a crucial role in actin filament polymerization that forms a nucleation site for starting actin filament polymerization and elongation^32^. Also, Arp2 of Arp2/3 complex performs ATP hydrolysis, which is very crucial in F-actin formation^33^. Overexpression of Arp2/3 complex was reported to be related to cancer and metastasis^34^. After BTZ and CFZ treatment, Arp2 expression elevation would be an acute response against destabilization of actin filaments instead of assisting motility. The decrease following 24 h and 48 h treatments of BTZ (Supplementary Figure 4) may be because of its strong damaging effect on actin structure (Fig. 2). Trangelin-2, a homolog of smooth muscle cell marker (SM22α), is an actin crosslinking protein and is involved in gel-sol transition of cytoplasm. Down regulation of trangelin-2 is related to elevated cell migration and cancer cell motility. Trangelin-2 was also shown to be involved in tube formation and actin stability^35^. Transgelin-2 expression was slightly increased following 3 h of CFZ treatment (Fig. 3D). This increase was seen only in CFZ and it can be explained with the CFZ related mild damage on actin structures, which could be repaired by transgelin-2 expression. Trangelin-2 was down and up regulated, respectively in both BTZ and CFZ treated groups following 24 h and 48 h treatments (Supplementary Figure 4B). The expression pattern of trangelin-2 was unsteady. This fluctuation is expected since transition of F-actin to G-actin is a dynamic process. In addition to these cytoskeletal proteins, there was no significant elevation in the expression of another F-actin cross-linking protein, coronin 1 C (or coronin 3) following 3 h of BTZ and CFZ treatments (Fig. 3E). However 24 h of CFZ treatment and 48 h of BTZ treatment decreased the amount of coronin 1 C (Supplementary Figure 4C), which is again confirming the dynamic process of actin structures.

Molecular assisting proteins are very important in correct folding of proteins. Basically there are three types of assisting proteins; molecular chaperones (broadly heat shock proteins), chaperonins and protein disulfide isomerases (PDI) & peptide prolyl cis-trans isomerase (PPI). While molecular chaperones are assisting and favoring folding of native or misfolded proteins, chaperonins are necessary for folding of their substrates. PDI and PPI perform shuffling of disulfide bonds and transition of cis-trans isomers, respectively^36^. PDIs have been used as anti-cancer drug targets in multiple myeloma cases^37^. Since PDIs are activated via unfolded protein accumulation^38^, proteasome inhibition is expected to increase PDI expression. An interesting point is that only proteasome inhibition by BTZ triggered elevation in PDI A3 and PDI A6 (Table 2). HSP70 is a crucial molecular chaperone and its expression is activated with protein unfolding and protein aggregation^39^. Our group has shown that HSP70 has a crucial role in stress response by assisting 26S protesome association and dissociation^40^. HSP32 (HO-1), in addition to its role in heme degradation, has protective functions against oxidative agents with its free –SH group^41^. Elevation in both HSP70 and HSP32 (Fig. 4A,C) may point to increased oxidative stress that is caused by oxidized proteins. We have evaluated protein oxidation via protein carbonylation analysis and as shown in Fig. 6A, BTZ lead to higher levels of protein carbonylation when compared to CFZ-treated group. Less protein carbonylation may explain why HSP70 and HSP32 were not increased following CFZ treatment. On the other hand, higher accumulation of ubiquitinated proteins in BTZ treated group (Fig. 6B) confirms the stronger effect of BTZ on protein turnover. Csizmadia *et al*.^17^ has also linked neuropathic pain caused by proteasome inhibitors to the accumulation of ubiquitinated proteins in peripheral nervous system cells. In addition, since the degree of inhibition in the proteasome activities was not different in BTZ- and CFZ-treated groups (Fig. 1), higher accumulation of protein carbonyls and ubiquitinated proteins (Fig. 6) may be due to less specificity and off-target inhibition of BTZ.

Proteasomal system plays an important role in ER-associated degradation (ERAD) of damaged, misfolded or uncorrectly folded proteins. When proteasome activity is inhibited via proteasome inhibitors, an elevation in ER stress is expected. Additionally, ER-stress related apoptosis was reported following proteasome inhibition by BTZ^42^. Since BTZ and CFZ affected protein oxidation and PDI expressions differently, ER-stress markers GRP78 and GRP94 levels were also detected in this study. Also KEGG pathway analysis showed that only BTZ treated group affected the protein processing in ER. According to our data, proteasome inhibition by both BTZ and CFZ caused an elevation in ER stress markers but no significant differences between BTZ and CFZ were detected (Fig. 4D,E).

The last experiment of this study showed the interaction of HSP70 with actin. Previously, HSP70 was reported to perform capping activity for F-actin and assist F-actin stabilization in heat stress conditions^43^. Recently, it was shown that HSP70 interacts with oxidized β-actin monomers and increases their interaction with proteasome for degradation^44^. Since actin filament stabilization was impaired following BTZ and CFZ treatment, immunoprecipitation was performed to show HSP70 and β-actin interaction. It can be concluded that proteasome inhibition increases oxidation in neural cells and these oxidized components cause destabilization of F-actin and oxidation of β-actin monomers. With this data, we can hypothesize that HSP70 interacts with β-actin for transporting it to the proteasome but since the proteasomal system is inhibited, interacted HSP70 - β-actin remains in the cytosol. On the other hand, protein carbonylation and accumulated ubiquitinated protein levels were much higher in the BTZ-treated group. But HSP70 interaction with β-actin was not specific for BTZ group and HSP70 - β-actin interaction was similar in both groups. Ratio of precipitated HSP70 to control was lower in BTZ group (Fig. 5B) because HSP70 expression level was much higher in BTZ treated group. For this reason, we thought that the ratio of precipitated β-actin to control β-actin (Figure 5A) would be more reliable than the ratio of precipitated HSP70 to control HSP70 (Fig. 5B).

As conclusion, like many other chemotherapeutic agents peripheral neuropathy is a problematic side effect of BTZ and second-generation inhibitors have been developed to decrease its side effects. In this study we compared the proteomic data of BTZ- and CFZ-treated cells to highlight the lower toxic effects of CFZ on NSCs. According to our data, the most important affected proteins are related to cytoskeletal proteins, molecular chaperones, sulfhydryl isomerases and the cellular antioxidant system. At the same levels of proteasome inhibition, BTZ caused stronger damage on actin structure, and increased vimentin, HSP32, HSP47 and HSP70 expressions higher than CFZ when compared to CONT. BTZ also caused significantly higher protein carbonylation and accumulation of ubiquitinated proteins. To conclude, higher increase in cellular oxidation level seems to be the most significant result of BTZ treatment. Since vimentin is related to mitochondrial movement and also oxidative phosphorylation pathways were activated following CFZ treatment, energetic pathways and mitochondrial proteins will be analyzed as the next step of our research.

## Methods

### Isolation and culture of mouse neural stem cells (NSCs)

In accordance with the Swedish legislation, Uppsala University Ethics Committee for Laboratory Animals approved the animal protocol (Protocol No: C11/12). Additionally, animal protocol was approved by Ethics Committee of Marmara University, Istanbul (Protocol No: 81.2015mar). All experiments were performed in accordance with relevant guidelines and regulations.

Primary NSCs were used for *in vitro* analysis to minimize the cell specific phenotype and response differences, and also to avoid cell-signaling variability of immortalized cells. C57Bl/6J mice were used in this study, and embryonic stage (E) was based on plug date. Cells were isolated from E14 embryos according to the described procedures^45,46^.

In brief, E14 embryos were removed from the uterus and the cortex separated from brain regions was placed in cold Leibovitz’s L-15 medium. Dissociation was done mechanically through pipetting until all large fragments disappeared. The suspension was filtered through a 70μm cell strainer into a 50 ml falcon tube. Following centrifugation, supernatant was removed and the pellet was re-suspended in growing media containing DMEM/F12 GlutaMax medium with N2 supplement and 1% Pen/Strep. The cells were counted in a Bürker chamber and were plated on coated 10 cm petri dishes at an approximate density of 1 × 10^6^ cells/dish in growing media supplemented with 10 ng/mL fibroblast growth factor 2 (FGF-2, Peprotech), 10 ng/ml epithelial growth factor (EGF, Peprotech). The cells were incubated at 37 °C, with 5% CO_2_ and 92% humidity for 7 days. FGF-2 and EGF were re-added after 3–4 days. After 2–3 days, unattached cell aggregates (neurospheres) were formed and NSCs were cultured as non-adherent cells. NSCs are functionally characterized as cells with the capacity to proliferate, self-renew, and produce a large number of progeny that can differentiate into neurons, astrocytes and oligodendrocytes^47^. Characterization of NSCs was done by staining of Nestin and Sox-2 (Supplementary Figure 13).

### Culture of H2c9 cardiomyoblasts and BALB/3T3 embryo fibroblasts

H2c9 cardiomyoblasts (ATCC CRL-1446) and BALB/3T3 embryo fibroblasts (ATCC CCL-163) were cultured in Dulbecco’s modified Eagle’s medium supplemented with penicillin (100 units/ml), streptomycin (100 ug/ml), and 10% fetal calf serum in a humidified atmosphere of 5%CO2 and 95% air at 37 °C. The reason of chosing these cells was to distinguish the effects of inhibitors in non-neuronal cells. Expecially H2c9 cardiomyoblasts were used since these inhibitors are known to be cardiotoxic and BALB/3T3 embryo fibroblasts were used as a counterpart mouse cells to NSCs.

### BTZ and CFZ treatments

Fresh growing medium without FGF-2 and EGF were replaced before drug treatment procedure and NSCs were incubated for 24 hours without any treatment.

BTZ (ApexBio A2614) and CFZ (ApexBio A1933) were dissolved in dimethyl sulfoxide (DMSO) and then diluted in 1x PBS. For each treatment step, 1 mM stock solution was diluted to a final concentration of 100 nM. Total DMSO concentration did not exceed 0.1% and control groups (CONT) were also treated with the same concentration of DMSO. The cells were treated for 3 h, 24 h and 48 h according to experimental procedures.

### Analysis of proteasome activity by fluorometry

Proteasome activity was analyzed in protein extracts following the lysis of cells with a lysis buffer of 8.56 g sucrose, 0.6 g HEPES, 0.2 g MgCl_2_, 0.037 g EDTA prepared in 100 ml H_2_O. Supernatant was used to measure the activity and our reaction mixture included 225 mM Tris buffer (pH 7.8) containing 7.5 mM MgOAc, 7.5 mM MgCl2, 45 mM KCl, and 1 mM dithiothreitol. The fluorogenic peptide succinyl-LLVY-methyl coumarin was used as substrate at a concentration of 200 μM to measure chymotrypsin-like activity of the proteasome. ATP was added to the reaction mixture to measure 20 S + 26 S proteasome activities. The samples were incubated with the substrate at 37 °C for 30 min, and methyl coumarin (MCA) liberation was measured with a fluorescence reader at 360 nm excitation/485 nm emission (Filter Max F5 Multimode Microplate Reader). Results were calculated according to the standard curve that was prepared by using free MCA. The proteasome inhibitor lactacystin was used to exclude other protease activities with the final concentration of 20 µM in the reaction mixture. Both proteasome activities with or without ATP were calculated as the difference between the total activity and the remaining activity in the presence of lactacystin. Inhibition by lactacystin was found to be around 15%.

### NanoLC-MS/MS for protein identification

CONT, BTZ and CFZ groups were treated for 3 h, 24 h, 48 h and cells were lysed with lysis buffer, containing 10 mg Octyl β-D-glucopyranoside, 8.775 mg NaCl, 0.375 mg EDTA and 0.05 M 200 μl Tris-HCl in 1 ml PBS. 10 μl protease inhibitor cocktail and 1 ml lysis buffer were added per 10^6^ cells and lysates were sonicated for 30 minutes and shaked for 1 hour at 4 °C. The cell lysates were clarified by centrifugation for 30 minutes (10,000 × g, 4 °C). Total extracted proteins were quantified with DC Protein Assay Kit (BioRad) according to supplier’s instructions.

50 µg of extracted protein was mixed with 50 µl of 50% acetonitrile containing 8 M urea, and then sonicated for homogenization. 100 µl of 100 mM ammonium bicarbonate and 10 µl of 45 mM DTT were added to the mixture, vortexed shortly, and incubated for 20 minutes at 56 °C. Spin columns were washed with 20% 200 µl acetonitrile, then 300 μl MQ water, and centrifuged after each washing step (20 minutes, 20 °C, 14,000 × g). After incubation of the mixture, 100 mM 10 µL Indole-3-acetic acid (IAA) was added into the mixture and incubated for 30 minutes at the room temperature. The mixture was transferred to the spin column and centrifuged for 20 minutes (14,000 × g, 20 °C). The spin columns were re-washed with 250 µl of 50 mM ammonium bicarbonate dissolved in 20% acetonitrile twice and 150 µL of 50 mM ammonium bicarbonate solution and each washing step was followed by centrifugation for 20 minutes (14,000 × g, 20 °C). 20 µL trypsin (20 µg trypsin dissolved in 200 µL 50 mM ammonium bisulfate solution) and 100 µL 50 mM ammonium bisulfate were added to the spin column and the mixture was incubated overnight at 37 °C. 100 µl of 1% acetic acid in 50% acetonitrile solution was added to filters and centrifuged for 20 min at 14,000 × g. The samples were dried in speedvac at 30 °C.

The nanoLC-MS/MS experiments were performed using a Orbitrap Velos Pro™ Hybrid Ion Trap-Orbitrap Mass Spectrometer and EASY-nLC (ThermoFisher Scientific). The LC setup was connected to an LTQ-Orbitrap mass spectrometer equipped with a nano-flex ion source (Proxeob Biosystems). Peptide mixtures were separated with an EASY-Column C18-A2 (Thermo Scientific, 75 μm inner and 3 μm outer diameters). The injection volumes were 5 μl and corresponded to 1.8 μg of tryptic peptides. The separations were performed at a flow of 200 nl/min with mobile phases A (water with 0.1% FA) and B (99.9% acetonitrile, 0.1% FA). 90-min gradient from 2% B to 100% B was followed by a washing step with 100% B for 3 min and 2%B for 5 min. Mass spectrometric analyses were performed using data-dependent mode in which the mass spectrometer automatically switches between acquiring a high resolution survey mass spectrum in the FTMS and consecutive low-resolution, CID fragmentation of up to ten most abundant ions in the Ion Trap (IT). For the low-resolution CID, full-scan MS spectra (from m/z 400 to 2000) were acquired in the Orbitrap analyzer. A normalized collision energy of 35%, activation time of 10 ms and activation q = 0.25 for MS2 were set and the resulting fragment ions were scanned out in the low pressure IT at the ‘normal scan rate’ and recorded with the secondary electron multipliers.

Acquired data (.RAW-files) were converted to the .mgf format using in-house written program (C++) and subjected to protein identification using Proteome Discoverer MASCOT search engine (version 2.2.2, Matrix Science, UK) against SwissProt database version 51.6. The search parameters were set to Taxonomy: Mus musculus, Enzyme: Trypsin, Fixed modifications: Carbamidomethyl (C), Variable modifications: Oxidation (M) and Deamidated (NQ), Peptide tolerance: ±0.01 Da, MS/MS tolerance: 0.6 Da and maximum two missed cleavage sites.

To identify biological processes changed by BTZ and/or CFZ treatment, STRING:functional protein association networks (v.10.0) analysis was performed for gene ontology (GO) and pathway terms (Szklarczyk *et al*.^16^). P values were calculated with software and values of p < 0.05 were selected as significant.

### Confocal microscopy

The cells were left for 72 h without growth factors to become adherent and 3 h of drug treatment were performed for actin staining and 24 h of drug treatment were performed for tubulin staining. These time points were chosen according to the proteomics data.

The cells were fixed with 1:1 diethyleter and ethanol solution at room temperature for 1 min, and blocked with washing solution (PBS containing 1% FCS) at room temperature for 30 min. Cells were then incubated with primary antibody in washing solution for 3 h. Antibodies detecting β-actin (D6A8, dilution 1:200), and β-tubulin (ab6046, dilution 1:200) were purchased from Cell Signaling Technology and Abcam, respectively. After incubation, the cells were washed three times and further incubated for 3 h with fluorescence-labeled secondary antibody (AlexaFluor 488 anti-rabbit IgG, Molecular Probes) at 1:100 dilution to visualize the target protein. Confocal fluorescence images were recorded with an “LSM 780 Meta” confocal microscope (Carl Zeiss, Jena, Germany). Pictures of random fields were captured for quantification using Image-J software (NIH). In general, randomly selected nuclear fields were evaluated for β-tubulin staining and randomly selected cells were evaluated β-actin staining. Area calculations and pixel densities were later analyzed and illustrations were prepared using GraphPad Prism 7.04 (GraphPad Software, Inc.). P-values less than 0.05 were selected as the level of significance.

For cell characterization, isolated cells were incubated at 37 °C, with 5% CO_2_ and 92% humidity for 7 days in the presence of EGF and FGF-2. Then, the cells were left for 72 h without growth factors to become adherent and confocal microscopy protocol was followed. Cells were labeled with nestin (sc-23927, dilution 1:100) and Sox-2 (CST-3579, dilution 1:400) antibodies. Target proteins were visualized with fluorescence-labeled secondary antibodies (anti-rabbit IgG-FITC, dilution 1:100, sc-2359; or anti-mouse IgG-Alexa Fluor 594, dilution 1:100, CST-8890).

### Immunoblotting analysis

Total protein isolation was performed following the treatment of BTZ and CFZ. Protein concentration of samples was determined with Pierce™ BCA Protein Assay Kit (23225 Thermo Fisher Scientific Inc.). Proteins from cell extracts in reducing Laemmli buffer (40 μg) were applied to SDS-PAGE (7% acrylamide) followed by blotting on nitrocellulose membranes. Bands were visualized by Pierce™ ECL Western Blotting Substrate (32106 Thermo Fisher Scientific Inc.) following the use of HRP-conjugated secondary antibodies (Cell Signaling Technology). Imaging was performed using ChemiDoc™ MP System (Bio-Rad Laboratories, Inc.). Lane band density was calculated with ChemiDoc™ MP System Software Image Lab Software.

Immunodetection of cytoskeleton members was performed using antibodies nestin (Abcam-ab6142, dilution 1:1000), vimentin (CST-3932, dilution 1:1000), actin related protein-2 (CST-ARP2 D85D5, dilution 1:1000), transgelin-2 (Abcam-ab184522, dilution 1:1000), and coronin 1 C (Abcam-ab153954, dilution 1:1000). GAPDH (Abcam-ab8245, dilution 1:1000) was detected as a loading control.

Immunodetection of heat shock proteins was performed using antibodies HSP27 (CST-G31, dilution 1:1000), HSP40 (CST-C64B4, dilution 1:1000), HSP60 (CST-D307, dilution 1:1000), HSP70 (CST-D69, dilution 1:1000), HSP90 (CST-C45G5, dilution 1:1000), HSP32 (Enzo HO-1, dilution 1:1000) and HSP47 (Abcam-ab77609 dilution 1:1000). ER related heat shock proteins, monoclonal rabbit anti-GRP78 (BiP, C50B12, CST-3177) and rabbit anti-GRP94 (CST-2104) antibodies were used in 1:1000 dilutions.

Ubiquitinylated proteins were detected by polyclonal K48-linkage specific antibody (CST-4289) in 1:1000 dilution.

### Co-Immunoprecipitation analysis

Co-immunoprecipitation was performed in both directions for HSP70 and β-actin. Pierce Co-immunoprecipitation kit (Thermo Scientific Inc.) was used and 50 μg of HSP70 antibody (5A5 Thermo Scientific Inc.) and β-actin antibody (ab8227 Abcam) were conjugated with coupling resins according to manufacturer’s suggestions. Cell extracts were added on resin columns following lysis procedure. Cell extract and resin-antibody complex were incubated overnight at 4 °C. After incubation, columns were washed 3 times and then co-precipitated proteins were obtained with 30-μl elution buffer. The eluted material was kept for further analysis. The washing solution of resin-antibody conjugation step was stored for tracing non-bounded antibodies and elution step was repeated to control non-eluted proteins.

For immunoblotting analysis 10 μl of elution was used. Proteins in reducing Laemmli buffer were applied to SDS-PAGE of 7% acrylamide followed by blotting on nitrocellulose membranes. Lanes from HSP70 co-immunoprecipitation were detected with β-actin antibody (ab8227 Abcam), lanes from β-actin co-immunoprecipitation were detected with HSP70 antibody (5A5 Thermo Scientific Inc.). Same antibodies were used to detect β-actin and HSP70 from cell lysates.

### Protein carbonylation as a marker of protein oxidation

Proteins from cell extracts (40 μg per lane) were separated by SDS-PAGE and blotted on nitrocellulose membranes. The membranes were equilibrated in TBS (100 mM Tris, 150 mM NaCl, pH 7.5) containing 20% methanol for 5 min, washed in 2 M HCl for 5 min, incubated with 10 mM DNPH solution for 5 min, washed 3 × 5 min in 2 M HCl and washed 5 × 5 min in 50% methanol. The blocking was performed following DNPH treatment for one hour at room temperature. After blocking, membranes were incubated with rabbit anti-DNP antibody (Sigma-Aldrich, D9656, 1:2500 dilution) and then with HRP-conjugated secondary anti-rabbit antibody (Cell Signaling Technology) in 1:5000 dilution for 1 h at room temperature.

### Statistical analysis

Prism 7.04 (Graph-Pad) software was used for statistical analysis. One-way ANOVA was performed followed by multiple comparisons using Bonferroni’s multiple comparison tests to determine the statistical significances. P-values less than 0.05 were selected as the level of significance.

## Electronic supplementary material

Dataset 1

## References

[1] Jung T, Catalgol B, Grune T (2009). The Proteasomal System. Mol. Aspects Med..

[2] Adams J (2002). Development of the proteasome inhibitor PS-341. Oncologist.

[3] Manasanch EE, Orlowski RZ (2017). Proteasome inhibitors in cancer therapy. Nat. Rev. Clin. Oncol..

[4] Adams J (1999). Proteasome Inhibitors: A Novel Class of Potent and Effective Antitumor Agents. Cancer Res..

[5] Goy A, Gilles F (2004). Update on the proteasome inhibitor bortezomib in hematologic malignancies. Clin. Lymphoma.

[6] O’Connor OA (2005). Phase II clinical experience with the novel proteasome inhibitor bortezomib in patients with indolent non-Hodgkin’s lymphoma and mantle cell lymphoma. J. Clin. Oncol..

[7] Kane RC (2007). Bortezomib for the treatment of mantle cell lymphoma. Clin. Cancer Res..

[8] Robinson CR, Zhang H, Dougherty P (2014). Astrocytes, but not microglia, are activated in oxaliplatin and bortezomib-induced peripheral neuropathy in the rat. Neuroscience.

[9] Pei XY, Dai Y, Grant S (2004). Synergistic induction of oxidative injury and apoptosis in human multiple myeloma cells by the proteasome inhibitor bortezomib and histone deacetylase inhibitors. Clin. Cancer Res..

[10] Landowski TH, Megli CJ, Nullmeyer KD, Lynch RM, Dorr RT (2005). Mitochondrial-mediated disregulation of Ca2 + is a critical determinant of Velcade (PS-341/bortezomib) cytotoxicity in myeloma cell lines. Cancer Res..

[11] Richardson PG, Hideshima T, Anderson KC (2003). Bortezomib (PS-341): A Novel, First-in-Class Proteasome Inhibitor for the Treatment of Multiple Myeloma and Other Cancers. Cancer Control.

[12] Kaplan GS, Torcun CC, Grune T, Ozer NK, Karademir B (2017). Proteasome inhibitors in cancer therapy: Treatment regimen and peripheral neuropathy as a side effect. Free Radic. Biol. Med..

[13] Dimopoulos MA (2017). Carfilzomib or bortezomib in relapsed or refractory multiple myeloma (ENDEAVOR): an interim overall survival analysis of an open-label, randomised, phase 3 trial. Lancet Oncol..

[14] Tsakiri EN (2017). Milder degenerative effects of Carfilzomib vs. Bortezomib in the Drosophila model: a link to clinical adverse events. Sci. Rep..

[15] Cattaneo E, McKay R (1990). Proliferation and differentiation of neural stem cells regulated by nerve growth factor. Nature.

[16] Szklarczyk D (2015). STRINGv10: protein-protein interaction networks, integrated over the tree of life. Nucleic Acids Res..

[17] Csizmadia V (2008). Effect of an experimental proteasome inhibitor on the cytoskeleton, cytosolic protein turnover, and induction in the neuronal cells *in vitro*. Neurotoxicology.

[18] Zheng H, Xiao WH, Bennett GJ (2012). Mitotoxicity and bortezomib-induced chronic painful peripheral neuropathy. Exp. Neurol..

[19] Federspiel JD (2016). Specificity of Protein Covalent Modification by the Electrophilic Proteasome Inhibitor Carfilzomib in Human Cells. Mol. Cell Proteomics.

[20] Lee JJ, Swain SM (2006). Peripheral neuropathy induced by microtubule-stabilizing agents. J Clin. Oncol..

[21] Staff NP (2013). Bortezomib alters microtubule polymerization and axonal transport in rat dorsal root ganglion neurons. Neurotoxicology.

[22] Poruchynsky MS (2008). Proteasome inhibitors increase tubulin polymerization and stabilization in tissue culture cells: A possible mechanism contributing to peripheral neuropathy and cellular toxicity following proteasome inhibition. Cell Cycle.

[23] Dechat T (2008). Nuclear lamins: major factors in the structural organization and function of the nucleus and chromatin. Genes Dev..

[24] Yoon BC (2012). Local translation of extranuclear lamin B promotes axon maintenance. Cell.

[25] Park D (2010). Nestin is required for the proper self-renewal of neural stem cells. Stem Cells.

[26] Liem RKH (1993). Molecular biology of neuronal intermediate filaments. Curr. Opin. Cell Biol..

[27] Guo M (2013). The Role of Vimentin Intermediate Filaments in Cortical and Cytoplasmic Mechanics. Biophys. J..

[28] Lowery J (2016). Abnormal intermediate filament organization alters mitochondrial motility in giant axonal neuropathy fibroblasts. Mol. Biol. Cell.

[29] Ruangjaroon T, Chokchaichamnankit D, Srisomsap C, Svasti J, Paricharttanakul NM (2017). Involvement of vimentin in neurite outgrowth damage induced by fipronil in SH-SY5Y cells. Biochem. Biophys. Res. Commun..

[30] Shimada T, Fournier AE, Yamagata K (2013). Neuroprotective function of 14-3-3 proteins in neurodegeneration. BioMed Res. Int..

[31] Kamphuis W (2015). GFAP and Vimentin Deficiency Alters Gene Expression in Astrocytes and Microglia in Wild-Type Mice and Changes the Transcriptional Response of Reactive Glia in Mouse Model for Alzheimer’s Disease. Glia.

[32] Mullins RD, Kelleher JF, Xu J, Pollard TD (1998). Arp2/3 Complex from Acanthamoeba Binds Profilin and Cross-links Actin Filaments. Mol. Biol. Cell.

[33] le Clainche C, Pantaloni D, Carlier MF (2003). ATP hydrolysis on actin-related protein 2/3 complex causes debranching of dendritic actin arrays. Proc. Natl. Acad. Sci..

[34] Yamazaki D, Kurisu S, Takenawa T (2005). Regulation of cancer cell motility through actin reorganization. Cancer Sci..

[35] Xiao Yuan, Li Yuhua, Han Jing, Pan Yan, Tie Lu, Li Xuejun (2012). Transgelin 2 Participates in Lovastatin-Induced Anti-Angiogenic Effects in Endothelial Cells through a Phosphorylated Myosin Light Chain-Related Mechanism. PLoS ONE.

[36] Bozaykut P, Ozer NK, Karademir B (2014). Regulation of protein turnover by heat shock proteins. Free Radic. Biol. Med..

[37] Vatolin S (2016). Novel Protein Disulfide Isomerase Inhibitor with Anticancer Activity in Multiple Myeloma. Cancer Res..

[38] Noiva R (1999). Protein disulphide isomerase: the multifunctional redox chaperone of the endoplasmic reticulum. Semin. Cell Dev. Biol..

[39] Mayer MP, Bukau B (2005). Hsp70 chaperones: Cellular functions and molecular mechanism. Cell Mol. Life Sci..

[40] Grune T (2011). HSP70 Mediates Dissociation and Reassociation of the 26S Proteasome During Adaptation to Oxidative Stress. Free Radic. Biol. Med..

[41] Takahashi T, Morita K, Akagi R, Sassa S (2004). Heme Oxygenase-1: A Novel Therapeutic Target in Oxidative Tissue Injuries. Curr. Med. Chem..

[42] Fribley A, Wang CY (2006). Proteasome inhibitor induces apoptosis through induction of endoplasmic reticulum stress. Cancer Biol. Ther..

[43] Xiang W, Rensing L (1999). Changes in cell morphology and actin organization during heat shock in Dictyostelium discoideum: does HSP70 play a role in acquired thermotolerance?. FEMS Microbiol. Lett..

[44] Reeg S (2016). The molecular chaperone Hsp70 promotes the proteolytic removal of oxidatively damaged proteins by the proteasome. Free Radic. Biol. Med..

[45] Azari H, Sharififar S, Rahman M, Ansari S, Reynolds BA (2011). Establishing embryonic mouse neural stem cell culture using the neurosphere assay. J Vis. Exp..

[46] Louis SA, Mak CK, Reynolds BA (2013). Methods to culture, differentiate, and characterize neural stem cells from the adult and embryonic mouse central nervous system. Methods Mol. Biol..

[47] Jensen JB, Parmar M (2006). Strengths and Limitations of the Neurosphere Culture System. Mol. Neurobiol..

