# Supplementary material for: Proteomic approach for understanding milder neurotoxicity of Carfilzomib against Bortezomib
Source: Sci Rep. 2018 Nov 5;8:16318. doi: 10.1038/s41598-018-34507-3 (PMC6218500; doi:10.1038/s41598-018-34507-3)

Proteomic approach for understanding milder neurotoxicity of Carfilzomib against Bortezomib

Betul Karademir[1,*,&], Gulce Sari[1,7,&], Ayse Tarbin Jannuzzi[2], Sravani Musunuri[3], Grzegorz Wicher[4], Tilman Grune[5] , Jia Mi[3, 8], Husniye Hacioglu-Bay[6], Karin Forsberg-Nilsson[4], Jonas Bergquist[3] and Tobias Jung[5]

SUPPLEMENTARY TABLES

Supplementary Table 1: List of proteins detected only in CONT group and absent in BTZ and in CFZ groups following 24 h of treatment.

Protein IDs	Protein Names
A0A087WP83	Vigilin
A0A087WR38	NADH dehydrogenase [ubiquinone] 1 alpha subcomplex subunit 10, mitochondrial
A2A599	V-type proton ATPase 116 kDa subunit a isoform 1
A2A9X5	5(3)-deoxyribonucleotidase, cytosolic type
A2AAW9	Eukaryotic translation initiation factor 2 subunit 3, Y-linked;Eukaryotic translation initiation factor 2 subunit 3, X-linked
A2AER9	Polyglutamine-binding protein 1
A2AFS1	Serine--tRNA ligase, cytoplasmic
A2AFW6	Mitochondrial carrier homolog 2
A2AL12	Heterogeneous nuclear ribonucleoprotein A3
A2AL83	Aspartyl/asparaginyl beta-hydroxylase
A2ALV7	Proteasome-associated protein ECM29 homolog
A2AM65	Heterochromatin protein 1-binding protein 3
A2AMY5	Ubiquitin-associated protein 2
A2ATP5	Myelin expression factor 2
A2AUR7	Ras suppressor protein 1
A2AV19	Exosome complex component RRP4
A2BGI8	Peptidyl-prolyl cis-trans isomerase
A2CES4	U2 small nuclear ribonucleoprotein B
B1ARW4	NADH dehydrogenase [ubiquinone] iron-sulfur protein 5;NADH dehydrogenase [ubiquinone] iron-sulfur protein 5, N-terminally processed
B2RY56	RNA-binding protein 25
D3YTN3	Methylsterol monooxygenase 1
D3YV43	40S ribosomal protein S3
D3YVC1	40S ribosomal protein S2
D3YVH4	Splicing factor 1
D3YWT1	Heterogeneous nuclear ribonucleoprotein H3
D3YXK2	Scaffold attachment factor B1
D3YZ19	DNA-directed RNA polymerase II subunit RPB11
D3Z0T5	Protein canopy homolog 4
D3Z1H8	Glycolipid transfer protein
D3Z1U9	NADH dehydrogenase [ubiquinone] flavoprotein 1, mitochondrial
D6RCG1	Trifunctional purine biosynthetic protein adenosine-3;Phosphoribosylamine--glycine ligase;Phosphoribosylformylglycinamidine cyclo-ligase;Phosphoribosylglycinamide formyltransferase
D6RGA5	Pre-mRNA-splicing factor SPF27
D6RJ77	Peptidyl-prolyl cis-trans isomerase-like 4
D9J2V6	Pre-B-cell leukemia transcription factor 1
E0CXB1	Proteasome subunit alpha type-6
E0CXW7	Probable tRNA N6-adenosine threonylcarbamoyltransferase
E0CXZ0	Nucleolar protein 56
E0CZE0	NEDD8-activating enzyme E1 regulatory subunit
E9PVP3	Mannose-P-dolichol utilization defect 1 protein
E9PWA7	Actin-related protein 2/3 complex subunit 4
E9PXX7	Thioredoxin domain-containing protein 5
E9Q055	Glyoxalase domain-containing protein 4
E9Q1J7	Propionyl-CoA carboxylase beta chain, mitochondrial
E9Q2H8	Hydroxyacylglutathione hydrolase, mitochondrial

E9Q2Z4	Non-histone chromosomal protein HMG-17
E9Q3T7	Adenylosuccinate lyase
E9Q3V6	Septin-2
E9Q5I9	26S proteasome non-ATPase regulatory subunit 13
E9Q6F4	Puromycin-sensitive aminopeptidase
E9Q6Z0	Cullin-5
E9Q7L0	2-oxoglutarate dehydrogenase, mitochondrial
E9QAK5	Kinesin heavy chain isoform 5C;Kinesin-1 heavy chain;Kinesin heavy chain isoform 5A
F6QA74	DNA-(apurinic or apyrimidinic site) lyase;DNA-(apurinic or apyrimidinic site) lyase, mitochondrial
F6U775	Dynamin-like 120 kDa protein, mitochondrial;Dynamin-like 120 kDa protein, form S1
F6USD5	Mesencephalic astrocyte-derived neurotrophic factor
F6VF36	Signal recognition particle receptor subunit alpha
F6VVY4	Tricarboxylate transport protein, mitochondrial
F6XIG5	Coatomer subunit epsilon
F6ZFT1	Acyl carrier protein;Acyl carrier protein, mitochondrial
F7ALS6	Aspartate aminotransferase, cytoplasmic
G3UXJ6	Heterogeneous nuclear ribonucleoprotein Q
G3UYD0	General transcription factor II-I
G3UZJ5	Casein kinase II subunit beta
G3UZL6	SUMO-conjugating enzyme UBC9
G5E8G0	Heterogeneous nuclear ribonucleoprotein D, isoform CRA_b
G5E919	NEDD8-conjugating enzyme Ubc12
H3BJD4	Disks large-associated protein 4
H3BJP2	S-formylglutathione hydrolase
H3BKW0	Cleavage and polyadenylation specificity factor subunit 6
H3BL56	Rho-related GTP-binding protein RhoC
H7BWX9	Small ubiquitin-related modifier 2
H7BWZ3	Actin-related protein 2/3 complex subunit 3
M0QWF0	Zinc finger protein 207
M0QWX0	Methionine aminopeptidase;Methionine aminopeptidase 2
O35344	Importin subunit alpha-4
O35465	Peptidyl-prolyl cis-trans isomerase FKBP8
O35685	Nuclear migration protein nudC
O35737	Heterogeneous nuclear ribonucleoprotein H;Heterogeneous nuclear ribonucleoprotein H, N-terminally processed
O35841	Apoptosis inhibitor 5
O54984	ATPase Asna1
O55135	Eukaryotic translation initiation factor 6
O55143-2	Sarcoplasmic/endoplasmic reticulum calcium ATPase 2
O70274	Protein tyrosine phosphatase type IVA 2
O88696	Putative ATP-dependent Clp protease proteolytic subunit, mitochondrial
P02802	Metallothionein-1
P08226	Apolipoprotein E
P10649	Glutathione S-transferase Mu 1
P12970	60S ribosomal protein L7a
P14115	60S ribosomal protein L27a
P14131	40S ribosomal protein S16
P15105	Glutamine synthetase
P20108	Thioredoxin-dependent peroxide reductase, mitochondrial
P21956-2	Lactadherin
P23780	Beta-galactosidase
P24668	Cation-dependent mannose-6-phosphate receptor
P25206	DNA replication licensing factor MCM3
P27641	X-ray repair cross-complementing protein 5
P27661	Histone H2A.x

P28474	Alcohol dehydrogenase class-3
P28659-2	CUGBP Elav-like family member 1
P28667	MARCKS-related protein
P31324	cAMP-dependent protein kinase type II-beta regulatory subunit;cAMP-dependent protein kinase type II-alpha regulatory subunit
P32067	Lupus La protein homolog
P35762	CD81 antigen
P37040	NADPH--cytochrome P450 reductase
P41105	60S ribosomal protein L28
P42669	Transcriptional activator protein Pur-alpha
P43277	Histone H1.3; Histone H1.4; Histone H1.5
P45376	Aldose reductase
P45878	Peptidyl-prolyl cis-trans isomerase FKBP2
P46061	Ran GTPase-activating protein 1
P46978	Dolichyl-diphosphooligosaccharide--protein glycosyltransferase subunit STT3A
P47856-2	Glutamine--fructose-6-phosphate aminotransferase [isomerizing] 1;Glutamine--fructose-6-phosphate aminotransferase [isomerizing] 2
P47941	Crk-like protein
P47962	60S ribosomal protein L5
P48771	Cytochrome c oxidase subunit 7A2, mitochondrial
P49722	Proteasome subunit alpha type-2
P50543	Protein S100-A11
P50580	Proliferation-associated protein 2G4
P51807	Dynein light chain Tctex-type 1
P51881	ADP/ATP translocase 2;ADP/ATP translocase 2, N-terminally processed
P53986	Monocarboxylate transporter 1
P54775	26S protease regulatory subunit 6B
P54823	Probable ATP-dependent RNA helicase DDX6
P56183-2	Ribosomal RNA processing protein 1 homolog A
P56812	Programmed cell death protein 5
P57759	Endoplasmic reticulum resident protein 29
P57780	Alpha-actinin-4; Alpha-actinin-1
P58044	Isopentenyl-diphosphate Delta-isomerase 1
P60843	Eukaryotic initiation factor 4A-I
P61087	Ubiquitin-conjugating enzyme E2 K
P61971	Nuclear transport factor 2
P62141	Serine/threonine-protein phosphatase PP1-beta catalytic subunit;Serine/threonine-protein phosphatase PP1-gamma catalytic subunit;Serine/threonine-protein phosphatase PP1-alpha catalytic subunit
P62267	40S ribosomal protein S23
P62301	40S ribosomal protein S13
P62334	26S protease regulatory subunit 10B
P62717	60S ribosomal protein L18a
P62814	V-type proton ATPase subunit B, brain isoform
P62827	GTP-binding nuclear protein Ran;GTP-binding nuclear protein Ran, testis-specific isoform
P63328-2	Serine/threonine-protein phosphatase 2B catalytic subunit alpha isoform
P84104-2	Serine/arginine-rich splicing factor 3
P97447	Four and a half LIM domains protein 1
P97822-2	Acidic leucine-rich nuclear phosphoprotein 32 family member E
Q00PI9	Heterogeneous nuclear ribonucleoprotein U-like protein 2
Q02819	Nucleobindin-1
Q05186	Reticulocalbin-1
Q05816	Fatty acid-binding protein, epidermal
Q05D44	Eukaryotic translation initiation factor 5B
Q06335-2	Amyloid-like protein 2
Q3THS6	S-adenosylmethionine synthase isoform type-2
Q3THW5	Histone H2A.V; Histone H2A. Z; Histone H2A

Q3TIX9	U4/U6.U5 tri-snRNP-associated protein 2
Q3TLQ0	Microtubule-associated protein
Q3TUE1	Far upstream element-binding protein 1
Q3U0V1	Far upstream element-binding protein 2
Q3U1J4	DNA damage-binding protein 1
Q3UJB0	Splicing factor 3b, subunit 2
Q3UKJ7-2	WD40 repeat-containing protein SMU1
Q3ULG5	DNA replication licensing factor MCM6
Q3V3R1	Monofunctional C1-tetrahydrofolate synthase, mitochondrial
Q501J6-2	Probable ATP-dependent RNA helicase DDX17
Q52KR6	Apoptotic chromatin condensation inducer in the nucleus
Q5SSW2-2	Proteasome activator complex subunit 4
Q5SUR0	Phosphoribosylformylglycinamidine synthase
Q60631-2	Growth factor receptor-bound protein 2
Q60692	Proteasome subunit beta type-6
Q60715-2	Prolyl 4-hydroxylase subunit alpha-1
Q60817	Nascent polypeptide-associated complex subunit alpha;Nascent polypeptide-associated complex subunit alpha, muscle-specific form
Q60870	Receptor expression-enhancing protein 5
Q60967	Bifunctional 3-phosphoadenosine 5-phosphosulfate synthase 1;Sulfate adenylyltransferase;Adenylyl-sulfate kinase
Q61035	Histidine--tRNA ligase, cytoplasmic
Q61036-2	Serine/threonine-protein kinase PAK 3;Serine/threonine-protein kinase PAK 2;PAK-2p27; PAK-2p34; Serine/threonine-protein kinase PAK 1
Q61074	Protein phosphatase 1G
Q61164	Transcriptional repressor CTCF
Q61166	Microtubule-associated protein RP/EB family member 1
Q61249	Immunoglobulin-binding protein 1;Immunoglobulin-binding protein 1b
Q61335	B-cell receptor-associated protein 31
Q61656	Probable ATP-dependent RNA helicase DDX5
Q61686	Chromobox protein homolog 5
Q62241	U1 small nuclear ribonucleoprotein C
Q62261-2	Spectrin beta chain, non-erythrocytic 1
Q62418-3	Drebrin-like protein
Q62425	NADH dehydrogenase [ubiquinone] 1 alpha subcomplex subunit 4
Q64445	Cytochrome c oxidase subunit 8A, mitochondrial
Q68FL6	Methionine--tRNA ligase, cytoplasmic
Q6A4J8-3	Ubiquitin carboxyl-terminal hydrolase 7;Ubiquitin carboxyl-terminal hydrolase
Q6P1B1	Xaa-Pro aminopeptidase 1
Q6P5F9	Exportin-1
Q6PBC0	Phosphatidate cytidylyltransferase 2
Q6TEK5	Vitamin K epoxide reductase complex subunit 1-like protein 1
Q6ZWY8	Thymosin beta-10
Q71LX4	Talin-2
Q7TMR0	Lysosomal Pro-X carboxypeptidase
Q7TPM0	Chromobox protein homolog 1
Q7TT37	Elongator complex protein 1
Q80SZ7	Guanine nucleotide-binding protein G(I)/G(S)/G(O) subunit gamma-5
Q80VD1	Protein FAM98B
Q80W54	CAAX prenyl protease 1 homolog
Q80X50-2	Ubiquitin-associated protein 2-like
Q80ZM5	H1 histone family, member X
Q8BFR4	N-acetylglucosamine-6-sulfatase
Q8BFZ3	Beta-actin-like protein 2
Q8BG30	Negative elongation factor A
Q8BGB7	Enolase-phosphatase E1
Q8BGC4	Zinc-binding alcohol dehydrogenase domain-containing protein 2
Q8BHC9	Alpha-(1,3)-fucosyltransferase 11

Q8BHZ0	Protein FAM49A
Q8BJ71	Nuclear pore complex protein Nup93
Q8BMJ3	Eukaryotic translation initiation factor 1A, X-chromosomal;Eukaryotic translation initiation factor 1A
Q8BTU6	Eukaryotic initiation factor 4A-II
Q8BWF0	Succinate-semialdehyde dehydrogenase, mitochondrial
Q8BWY3	Eukaryotic peptide chain release factor subunit 1
Q8BXA1	Golgi integral membrane protein 4
Q8BZN7	Thyroid hormone receptor-associated protein 3
Q8C1B7-3	Septin-11
Q8C7K6	Prenylcysteine oxidase-like
Q8C878-2	NEDD8-activating enzyme E1 catalytic subunit
Q8CDN6	Thioredoxin-like protein 1
Q8CGK3	Lon protease homolog, mitochondrial
Q8K1K2	26S protease regulatory subunit 8
Q8K2T1-2	NmrA-like family domain-containing protein 1
Q8K310	Matrin-3
Q8K3J1	NADH dehydrogenase [ubiquinone] iron-sulfur protein 8, mitochondrial
Q8K4F5	Alpha/beta hydrolase domain-containing protein 11
Q8QZY9	Splicing factor 3B subunit 4
Q8R0F6	Integrin-linked kinase-associated serine/threonine phosphatase 2C
Q8R0H9	ADP-ribosylation factor-binding protein GGA1
Q8R0Y6	Cytosolic 10-formyltetrahydrofolate dehydrogenase
Q8R127	Saccharopine dehydrogenase-like oxidoreductase
Q8R180	ERO1-like protein alpha
Q8VDM6-2	Heterogeneous nuclear ribonucleoprotein U-like protein 1
Q8VE97	Serine/arginine-rich splicing factor 4
Q8VH51-3	RNA-binding protein 39
Q8VHM5	Heterogeneous nuclear ribonucleoprotein R
Q8VIM9	Immunity-related GTPase family Q protein
Q91WQ3	Tyrosine--tRNA ligase, cytoplasmic;Tyrosine--tRNA ligase, cytoplasmic, N-terminally processed
Q91YM2	Rho GTPase-activating protein 35
Q91YQ5	Dolichyl-diphosphooligosaccharide--protein glycosyltransferase subunit 1
Q91YZ2	C-terminal-binding protein 2
Q91Z67	SLIT-ROBO Rho GTPase-activating protein 2
Q921L3	Transmembrane and coiled-coil domain-containing protein 1
Q922D8	C-1-tetrahydrofolate synthase, cytoplasmic;Methylenetetrahydrofolate dehydrogenase;Methenyltetrahydrofolate cyclohydrolase;Formyltetrahydrofolate synthetase;C-1-tetrahydrofolate synthase, cytoplasmic, N-terminally processed
Q922Q8	Leucine-rich repeat-containing protein 59
Q922Y1	UBX domain-containing protein 1
Q99JF5	Diphosphomevalonate decarboxylase
Q99JI4	26S proteasome non-ATPase regulatory subunit 6
Q99JY8	Lipid phosphate phosphohydrolase 3
Q99KF1	Transmembrane emp24 domain-containing protein 9
Q99L27	GMP reductase 2
Q99L45	Eukaryotic translation initiation factor 2 subunit 2
Q99LC5	Electron transfer flavoprotein subunit alpha, mitochondrial
Q99LF4	tRNA-splicing ligase RtcB homolog
Q99LI7	Cleavage stimulation factor subunit 3
Q99LP6	GrpE protein homolog 1, mitochondrial
Q99M87-3	DnaJ homolog subfamily A member 3, mitochondrial
Q99MN1	Lysine--tRNA ligase
Q99MR6-3	Serrate RNA effector molecule homolog
Q99NB1	Acetyl-coenzyme A synthetase 2-like, mitochondrial
Q99P72-3	Reticulon-4
Q99PT1	Rho GDP-dissociation inhibitor 1

Q9CPU0	Lactoylglutathione lyase
Q9CQ65	S-methyl-5-thioadenosine phosphorylase
Q9CQC6	Basic leucine zipper and W2 domain-containing protein 1
Q9CQC9	GTP-binding protein SAR1b
Q9CQE8	UPF0568 protein C14orf166 homolog
Q9CQF3	Cleavage and polyadenylation specificity factor subunit 5
Q9CQH7	Transcription factor BTF3 homolog 4
Q9CQK7	RWD domain-containing protein 1
Q9CQL7	MORF4 family-associated protein 1
Q9CQM2	ER lumen protein retaining receptor 2
Q9CQN1	Heat shock protein 75 kDa, mitochondrial
Q9CQR6	Serine/threonine-protein phosphatase 6 catalytic subunit
Q9CQT1	Methylthioribose-1-phosphate isomerase
Q9CQZ1	Heat shock factor-binding protein 1
Q9CR20	Immediate early response 3-interacting protein 1
Q9CR51	V-type proton ATPase subunit G 1
Q9CT10	Ran-binding protein 3
Q9CWJ9	Bifunctional purine biosynthesis protein PURH;Phosphoribosylaminoimidazolecarboxamide formyltransferase;IMP cyclohydrolase
Q9CWZ3-2	RNA-binding protein 8A
Q9CXS4	Centromere protein V
Q9CXU9	Eukaryotic translation initiation factor 1b
Q9CZY3-2	Ubiquitin-conjugating enzyme E2 variant 1
Q9D0T1	NHP2-like protein 1;NHP2-like protein 1, N-terminally processed
Q9D173	Mitochondrial import receptor subunit TOM7 homolog
Q9D6V8	Polyadenylate-binding protein-interacting protein 2
Q9D7A8	Armadillo repeat-containing protein 1
Q9D883	Splicing factor U2AF 35 kDa subunit
Q9D892	Inosine triphosphate pyrophosphatase
Q9D8B3	Charged multivesicular body protein 4b
Q9D964	Glycine amidinotransferase, mitochondrial
Q9DAK9	14 kDa phosphohistidine phosphatase
Q9DAR7	m7GpppX diphosphatase
Q9DAW9	Calponin-3
Q9DBE0	Cysteine sulfinic acid decarboxylase
Q9DBH5	Vesicular integral-membrane protein VIP36
Q9DBR1-2	5-3 exoribonuclease 2
Q9DC70	NADH dehydrogenase [ubiquinone] iron-sulfur protein 7, mitochondrial
Q9DCD0	6-phosphogluconate dehydrogenase, decarboxylating
Q9DCN2-2	NADH-cytochrome b5 reductase 3;NADH-cytochrome b5 reductase 3 membrane-bound form;NADH-cytochrome b5 reductase 3 soluble form
Q9EP69	Phosphatidylinositide phosphatase SAC1
Q9EQH3	Vacuolar protein sorting-associated protein 35
Q9ERT9	Protein phosphatase 1 regulatory subunit 1A
Q9EST5	Acidic leucine-rich nuclear phosphoprotein 32 family member B
Q9ESW8	Pyroglutamyl-peptidase 1
Q9ET30	Transmembrane 9 superfamily member 3
Q9JHI5	Isovaleryl-CoA dehydrogenase, mitochondrial
Q9JIG8	PRA1 family protein 2
Q9JJ61	Polypeptide N-acetylgalactosaminyltransferase 16
Q9JJU8	SH3 domain-binding glutamic acid-rich-like protein
Q9JKC6	Cell cycle exit and neuronal differentiation protein 1
Q9JKV1	Proteasomal ubiquitin receptor ADRM1
Q9JL35	High mobility group nucleosome-binding domain-containing protein 5
Q9QUR6	Prolyl endopeptidase
Q9QXT0	Protein canopy homolog 2
Q9QYB1	Chloride intracellular channel protein 4

Q9QYG0-2	Protein NDRG2
Q9R062	Glycogenin-1
Q9R0H0-2	Peroxisomal acyl-coenzyme A oxidase 1
Q9R0N0	Galactokinase
Q9R0P5	Destrin
Q9R0P9	Ubiquitin carboxyl-terminal hydrolase isozyme L1
Q9R0Q7	Prostaglandin E synthase 3
Q9R1P0	Proteasome subunit alpha type-4
Q9R1P3	Proteasome subunit beta type-2
Q9R1T2-2	SUMO-activating enzyme subunit 1;SUMO-activating enzyme subunit 1, N-terminally processed
Q9R1T4-2	Septin-6
Q9WTX5	S-phase kinase-associated protein 1
Q9WU78	Programmed cell death 6-interacting protein
Q9WVF5	Epidermal growth factor receptor
Q9Z0H3-2	SWI/SNF-related matrix-associated actin-dependent regulator of chromatin subfamily B member 1
Q9Z130	Heterogeneous nuclear ribonucleoprotein D-like
Q9Z1N5	Spliceosome RNA helicase Ddx39b
Q9Z1X4	Interleukin enhancer-binding factor 3
Q9Z275	Retinaldehyde-binding protein 1
Q9Z2Q6	Septin-5
S4R2G9	Aldo-keto reductase family 1, member B10 (aldose reductase)

Supplementary Table 2: List of proteins detected only in BTZ group and absent in CONT and in CFZ groups following 24 h of treatment.

Protein IDs	Protein Names
A0A087WP63	Predicted gene 42715
A2AAN2	Signal recognition particle subunit SRP68
A2AFI9	Histone-binding protein RBBP7
A2AVJ7	Ribosome-binding protein 1
A6ZI44	Fructose-bisphosphate aldolase;Fructose-bisphosphate aldolase A
C0H5X4	GTPase HRas;GTPase HRas, N-terminally processed;GTPase KRas;GTPase KRas, N-terminally processed;GTPase NRas
D3YV37	Myosin, light chain 12A, regulatory, non-sarcomeric
D3YW48	Calpain small subunit 1
D3Z3Y3	Single-stranded DNA-binding protein; Single-stranded DNA-binding protein, mitochondrial
E9PVC6	Eukaryotic translation initiation factor 4 gamma 1
E9Q0W5	Protein FAM3C
E9Q5C9	Nucleolar and coiled-body phosphoprotein 1
E9QAS4	Chromodomain-helicase-DNA-binding protein 4
F6VQ81	Tumor protein D54
F6VSK8	Integrin alpha-6;Integrin alpha-6 heavy chain;Integrin alpha-6 light chain
F6WZH1	Methyl-CpG-binding domain protein 3
F7C265	Coiled-coil domain-containing protein 47
O08585	Clathrin light chain A
O35343	Importin subunit alpha-3
O55028	[3-methyl-2-oxobutanoate dehydrogenase [lipoamide]] kinase, mitochondrial
O88325	Alpha-N-acetylglucosaminidase
O88441	Metaxin-2
P02468	Laminin subunit gamma-1
P10637-4	Microtubule-associated protein tau
P16125	L-lactate dehydrogenase B chain;L-lactate dehydrogenase
P26369	Splicing factor U2AF 65 kDa subunit
P27546-2	Microtubule-associated protein 4;Microtubule-associated protein
P35802	Neuronal membrane glycoprotein M6-a
P46425	Glutathione S-transferase P 2;Glutathione S-transferase P 1
P52293	Importin subunit alpha-1
P60487	Pyridoxal phosphate phosphatase
P62311	U6 snRNA-associated Sm-like protein LSm3
P63073	Eukaryotic translation initiation factor 4E
P85094	Isochorismatase domain-containing protein 2A, mitochondrial
P97429	Annexin A4; Annexin
P97478	Ubiquinone biosynthesis protein COQ7 homolog
P99029-2	Peroxiredoxin-5, mitochondrial
Q60875-5	Rho guanine nucleotide exchange factor 2
Q61879	Myosin-10
Q62186	Translocon-associated protein subunit delta
Q62376-2	U1 small nuclear ribonucleoprotein 70 kDa
Q63810-2	Calcineurin subunit B type 1
Q64152-2	Transcription factor BTF3
Q68FL0	Sodium/calcium exchanger 1
Q6A028	Switch-associated protein 70
Q6IRU2	Tropomyosin alpha-4 chain
Q6P5F7-2	Protein tweety homolog 3
Q6PD26	GPI transamidase component PIG-S
Q6PIC6	Sodium/potassium-transporting ATPase subunit alpha-3
Q78ZA7	Nucleosome assembly protein 1-like 4

Q80V26	Inositol monophosphatase 3
Q80YX1-5	Tenascin
Q8BNY6	Neuronal calcium sensor 1
Q8BP27-2	Swi5-dependent recombination DNA repair protein 1 homolog
Q8BT60	Copine-3
Q8BUN5	Mothers against decapentaplegic homolog 3;Mothers against decapentaplegic homolog 2
Q8C129	Leucyl-cystinyl aminopeptidase
Q8C3Y4	Kinetochore-associated protein 1
Q8C7X2-2	ER membrane protein complex subunit 1
Q8JZK9	Hydroxymethylglutaryl-CoA synthase, cytoplasmic
Q8K1M6-5	Dynamin-1-like protein
Q8R1N4-3	NudC domain-containing protein 3
Q8R317	Ubiquilin-1
Q8VBV7	COP9 signalosome complex subunit 8
Q91WT9-2	Cystathionine beta-synthase
Q923D4	Splicing factor 3B subunit 5
Q99KI3	ER membrane protein complex subunit 3
Q99KV1	DnaJ homolog subfamily B member 11
Q99LB2	Dehydrogenase/reductase SDR family member 4
Q9CQN3	Mitochondrial import receptor subunit TOM6 homolog
Q9CQU0	Thioredoxin domain-containing protein 12
Q9CXZ1	NADH dehydrogenase [ubiquinone] iron-sulfur protein 4, mitochondrial
Q9CZW5	Mitochondrial import receptor subunit TOM70
Q9D6F9	Tubulin beta-4A chain
Q9D6J6-2	NADH dehydrogenase [ubiquinone] flavoprotein 2, mitochondrial
Q9D7M1	Glucose-induced degradation protein 8 homolog
Q9ER72-2	Cysteine--tRNA ligase, cytoplasmic
Q9ERY9	Probable ergosterol biosynthetic protein 28
Q9JIQ3	Diablo homolog, mitochondrial
Q9JKR6	Hypoxia up-regulated protein 1
Q9JKW0	ADP-ribosylation factor-like protein 6-interacting protein 1
Q9QUH0	Glutaredoxin-1
Q9QXS6-2	Drebrin
Q9QYC0-2	Alpha-adducin
Q9R0A0	Peroxisomal membrane protein PEX14
Q9WTM5	RuvB-like 2
Q9WTR5	Cadherin-13
Q9WV55	Vesicle-associated membrane protein-associated protein A
Q9Z0H4-3	CUGBP Elav-like family member 2
Q9Z0Y1	Dynactin subunit 3
Q9Z1Z2	Serine-threonine kinase receptor-associated protein
Q9Z2G6-2	Protein sel-1 homolog 1

Supplementary Table 3: List of proteins detected only in CFZ group and absent in CONT and in BTZ groups following 24 h of treatment.

**Protein IDs**	**Protein Names**
A0A087WQ34	Mortality factor 4-like protein 1
A0A087WQR6	Tripeptidyl-peptidase 2
A2AE03	Eukaryotic translation initiation factor 3 subunit I
A2AIW9	Mitochondrial-processing peptidase subunit alpha
B2RXS4	Plexin-B2
D3YU80	Cold-inducible RNA-binding protein
D3YZ18	High mobility group protein B1
E9PV44	ATPase inhibitor, mitochondrial
E9Q3G4	Sterile alpha motif domain-containing protein 3
E9Q7G1	Transmembrane p24-trafficking protein 7
F6RJ83	NADH dehydrogenase [ubiquinone] iron-sulfur protein 2, mitochondrial
F6YUH3	Branched-chain-amino-acid aminotransferase, cytosolic;Branched-chain-amino-acid aminotransferase
O70310	Glycylpeptide N-tetradecanoyltransferase 1
P12815-2	Programmed cell death protein 6
P42125	Enoyl-CoA delta isomerase 1, mitochondrial
P47963	60S ribosomal protein L13
P52503	NADH dehydrogenase [ubiquinone] iron-sulfur protein 6, mitochondrial
P62869	Transcription elongation factor B polypeptide 2
P62900	60S ribosomal protein L31
P62960	Nuclease-sensitive element-binding protein 1
P63001	Ras-related C3 botulinum toxin substrate 1;Ras-related C3 botulinum toxin substrate 3;Ras-related C3 botulinum toxin substrate 2
P63330	Serine/threonine-protein phosphatase 2A catalytic subunit alpha isoform;Serine/threonine-protein phosphatase 2A catalytic subunit beta isoform
P97315	Cysteine and glycine-rich protein 1
Q02780-2	Nuclear factor 1 A-type
Q3TDD9	Protein phosphatase 1 regulatory subunit 21
Q3UW83	40S ribosomal protein S10
Q5SX56	Sperm-associated antigen 7
Q60972	Histone-binding protein RBBP4
Q62351	Transferrin receptor protein 1
Q6P8I4	PEST proteolytic signal-containing nuclear protein
Q7M739	Nucleoprotein TPR
Q8K3C3	Protein LZIC
Q8R3V2	Aminoacyl tRNA synthase complex-interacting multifunctional protein 2
Q920A5	Retinoid-inducible serine carboxypeptidase
Q9CPS5	26S proteasome non-ATPase regulatory subunit 8
Q9CQ69	Cytochrome b-c1 complex subunit 8
Q9CQN6	Transmembrane protein 14C
Q9D0F9	Phosphoglucomutase-1
Q9D1E6	Tubulin-folding cofactor B
Q9D1K2	V-type proton ATPase subunit F
Q9D554	Splicing factor 3A subunit 3
Q9D6U8	Protein FAM162A
Q9DB20	ATP synthase subunit O, mitochondrial
Q9R0B9	Procollagen-lysine,2-oxoglutarate 5-dioxygenase 2
Q9WUK2-2	Eukaryotic translation initiation factor 4H

Supplementary Table 4: List of proteins detected in both CFZ and CONT groups, missing in BTZ group following 24 h of treatment.

Protein IDs	Protein Names
**A2AFK7**	Eukaryotic initiation factor 4A-III;Eukaryotic initiation factor 4A-III, N-terminally processed
**D3Z375**	Astrocytic phosphoprotein PEA-15
**E9PWE8**	Dihydropyrimidinase-related protein 3
**G3UYV7**	40S ribosomal protein S28
**O08795**	Glucosidase 2 subunit beta
**O55137**	Acyl-coenzyme A thioesterase 1;Acyl-coenzyme A thioesterase 3;Acyl-coenzyme A thioesterase 2, mitochondrial
**P05063**	Fructose-bisphosphate aldolase C
**P24527**	Leukotriene A-4 hydrolase
**P30416**	Peptidyl-prolyl cis-trans isomerase FKBP4;Peptidyl-prolyl cis-trans isomerase FKBP4, N-terminally processed
**P30681**	High mobility group protein B2
**P50518**	V-type proton ATPase subunit E 1
**P51174**	Long-chain specific acyl-CoA dehydrogenase, mitochondrial
**P59017**	Bcl-2-like protein 13
**P61924**	Coatomer subunit zeta-1
**P62322**	U6 snRNA-associated Sm-like protein LSm5
**P62774**	Myotrophin
**P62843**	40S ribosomal protein S15
**P68037**	Ubiquitin-conjugating enzyme E2 L3
**P99027**	60S acidic ribosomal protein P2
**Q3U6K9**	Phosphoserine aminotransferase
**Q5SW88**	Ras-related protein Rab-1A
**Q60931**	Voltage-dependent anion-selective channel protein 3
**Q61753**	D-3-phosphoglycerate dehydrogenase
**Q62084**	Protein phosphatase 1 regulatory subunit 14B
**Q62093**	Serine/arginine-rich splicing factor 2
**Q62318**	Transcription intermediary factor 1-beta
**Q6P5H2-2**	Nestin
**Q8BH59**	Calcium-binding mitochondrial carrier protein Aralar1
**Q8CHH9**	Septin-8
**Q8CIG8**	Protein arginine N-methyltransferase 5
**Q8JZQ9**	Eukaryotic translation initiation factor 3 subunit B
**Q8K4Z5**	Splicing factor 3A subunit 1
**Q91VM5**	RNA binding motif protein, X-linked-like-1
**Q91WK5**	Glycine cleavage system H protein, mitochondrial
**Q9CXY6**	Interleukin enhancer-binding factor 2
**Q9CY58-3**	Plasminogen activator inhibitor 1 RNA-binding protein
**Q9CYN2**	Signal peptidase complex subunit 2
**Q9CZD3**	Glycine--tRNA ligase
**Q9CZN7**	Serine hydroxymethyltransferase
**Q9D051**	Pyruvate dehydrogenase E1 component subunit beta, mitochondrial
**Q9D1D4**	Transmembrane emp24 domain-containing protein 10
**Q9WTP6-2**	Adenylate kinase 2, mitochondrial;Adenylate kinase 2, mitochondrial, N-terminally processed
**Q9WUM4**	Coronin-1C
**Q9Z1F9**	SUMO-activating enzyme subunit 2

Supplementary Table 5: List of proteins detected in both BTZ and CONT groups, missing in CFZ group following 24 h of treatment.

**Protein IDS**	**Protein Names**
E0CXB2	Importin-9
E9QA63	Microtubule-actin cross-linking factor 1
H3BL49	T-complex protein 1 subunit theta
O08583-2	THO complex subunit 4;Aly/REF export factor 2
O35286	Putative pre-mRNA-splicing factor ATP-dependent RNA helicase DHX15
O35593	26S proteasome non-ATPase regulatory subunit 14
O35604	Niemann-Pick C1 protein
O70503	Estradiol 17-beta-dehydrogenase 12
P08003	Protein disulfide-isomerase A4
P13439	Uridine 5-monophosphate synthase;Orotate phosphoribosyltransferase;Orotidine 5-phosphate decarboxylase
P14148	60S ribosomal protein L7
P20065-2	Thymosin beta-4;Hematopoietic system regulatory peptide
P24547	Inosine-5-monophosphate dehydrogenase 2
P26645	Myristoylated alanine-rich C-kinase substrate
P50247	Adenosylhomocysteinase
P51410	60S ribosomal protein L9
P61620	Protein transport protein Sec61 subunit alpha isoform 1;Protein transport protein Sec61 subunit alpha isoform 2
P61759	Prefoldin subunit 3
P63085	Mitogen-activated protein kinase 1;Mitogen-activated protein kinase 3
P70296	Phosphatidylethanolamine-binding protein 1;Hippocampal cholinergic neurostimulating peptide
Q3TVK3	Aspartyl aminopeptidase
Q4KML4	Costars family protein ABRACL
Q61990-2	Poly(rC)-binding protein 2
Q64522	Histone H2A type 2-B
Q6PDM2	Serine/arginine-rich splicing factor 1
Q6ZQ38	Cullin-associated NEDD8-dissociated protein 1
Q8BIJ6	Isoleucine--tRNA ligase, mitochondrial
Q8BMS1	Trifunctional enzyme subunit alpha, mitochondrial;Long-chain enoyl-CoA hydratase;Long chain 3-hydroxyacyl-CoA dehydrogenase
Q91Z31	Polypyrimidine tract-binding protein 2
Q921H8	3-ketoacyl-CoA thiolase A, peroxisomal;3-ketoacyl-CoA thiolase B, peroxisomal
Q99JR1	Sideroflexin-1
Q99KI0	Aconitate hydratase, mitochondrial
Q9CX86	Heterogeneous nuclear ribonucleoprotein A0
Q9DAS9	Guanine nucleotide-binding protein G(I)/G(S)/G(O) subunit gamma-12
Q9ERD7	Tubulin beta-3 chain
Q9JKK7	Tropomodulin-2
Q9Z110-2	Delta-1-pyrroline-5-carboxylate synthase;Glutamate 5-kinase;Gamma-glutamyl phosphate reductase

Supplementary Table 6: List of proteins detected in both BTZ and CFZ groups, missing in CONT group following 24 h of treatment.

Protein IDs	Protein Names
D3YUK4	NADH dehydrogenase [ubiquinone] 1 beta subcomplex subunit 10
P07356	Annexin A2
P09055	Integrin beta-1
P12787	Cytochrome c oxidase subunit 5A, mitochondrial
P14206	40S ribosomal protein SA
P17710-3	Hexokinase-1
P17918	Proliferating cell nuclear antigen
P31650	Sodium- and chloride-dependent GABA transporter 3
P43024	Cytochrome c oxidase subunit 6A1, mitochondrial;Cytochrome c oxidase subunit 6A, mitochondrial
P63037	DnaJ homolog subfamily A member 1
P68510	14-3-3 protein eta
Q61696	Heat shock 70 kDa protein 1A;Heat shock 70 kDa protein 1B
Q64433	10 kDa heat shock protein, mitochondrial
Q80X19-2	Collagen alpha-1(XIV) chain
Q91WK2	Eukaryotic translation initiation factor 3 subunit H
Q91ZX7	Prolow-density lipoprotein receptor-related protein 1;Low-density lipoprotein receptor-related protein 1 85 kDa subunit;Low-density lipoprotein receptor-related protein 1 515 kDa subunit;Low-density lipoprotein receptor-related protein 1 intracellular domain
Q9CQF9	Prenylcysteine oxidase
Q9D0M3-2	Cytochrome c1, heme protein, mitochondrial
Q9D1Q6	Endoplasmic reticulum resident protein 44
Q9D6R2-2	Isocitrate dehydrogenase [NAD] subunit alpha, mitochondrial
Q9D7N9	Adipocyte plasma membrane-associated protein

A

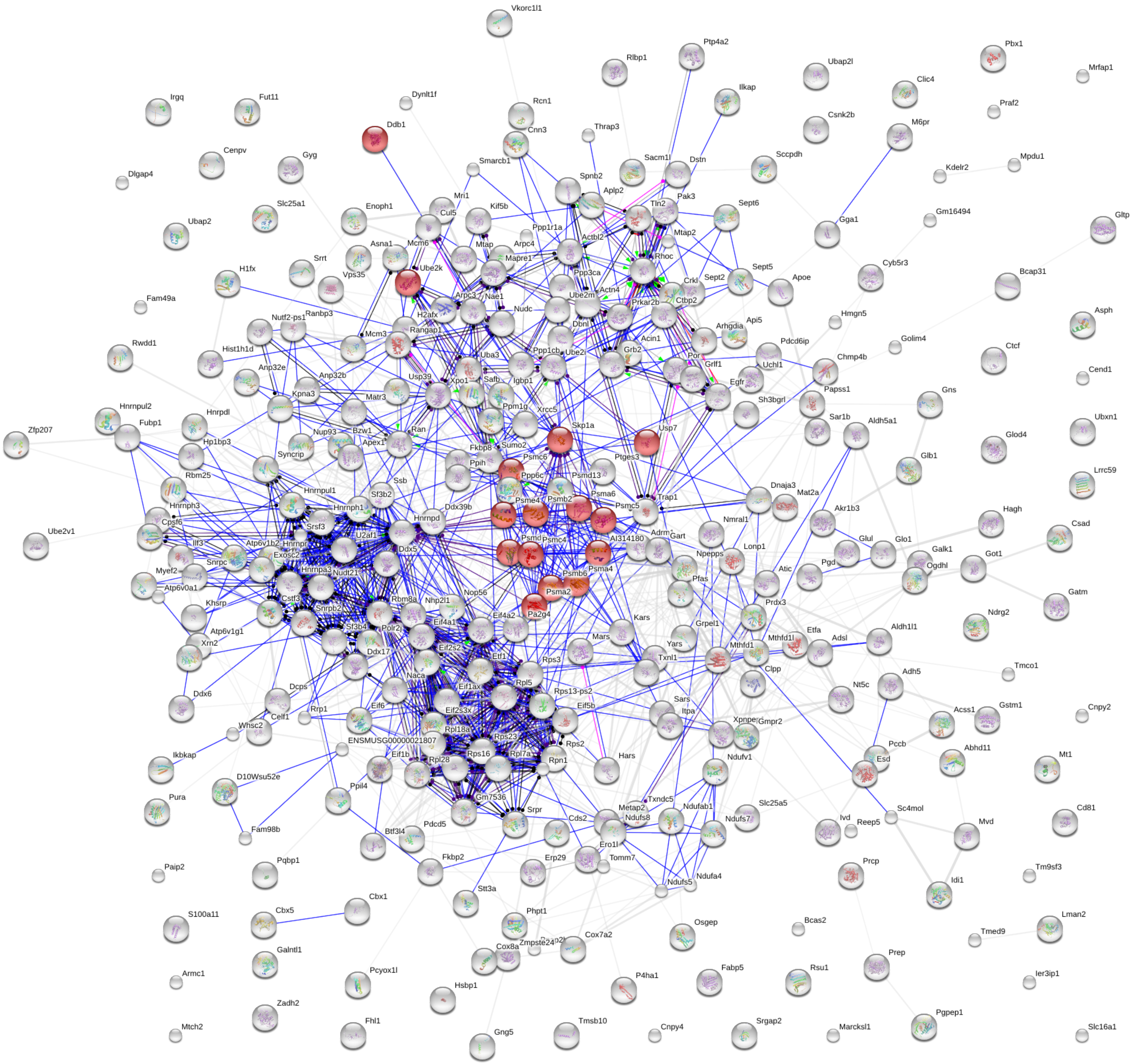
Vkorc1l1
Ptp4a2
Pbx1
Rlbp1
Ubap2l
Mrfap1
Ilkap
Clic4
Irgq
Fut11
Dynlt1f
Rcn1
Praf2
Csnk2b
Ddb1
Cnn3
Thrap3
M6pr
Cenpv
Gyg
Smarcb1
Sacm1l
Dstn
Sccpdh
Mpdu1
Dlgap4
Kdelr2
Spnb2
Aplp2
Pak3
Slc25a1
Mri1
Tln2
Sept6
Gm16494
Gltp
Ubap2
Enoph1
Cul5
Kif5b
Ppp1r1a
Mtap2
Gga1
Actbl2
Asna1
Mcm6
Mtap
Arpc4
Rhoc
Srrt
Mapre1
Cyb5r3
H1fx
Vps35
Ube2k
Ppp3ca
Sept5
Bcap31
Crkl
Sept2
Apoe
Fam49a
Actn4
Arpc3
Nae1
Ube2m
Ctbp2
Prkar2b
H2afx
Nudc
Hmgn5
Asph
Ranbp3
Nutf2-ps1
Api5
Rangap1
Dbnl
Arhgdia
Mcm3
Golim4
Acin1
Pdcd6ip
Ctcf
Rwdd1
Hist1h1d
Uba3
Ppp1cb
Ube2l
Grb2
Chmp4b
Anp32e
Por
Grlf1
Uchl1
Cend1
Usp39
Xpo1
Safb
Igbp1
Egfr
Anp32b
Papss1
Gns
Kpna3
Sh3bgrl
Matr3
Ppm1g
Hnrnpul2
Xrcc5
Hnrpdl
Skp1a
Ubxn1
Zfp207
Fubp1
Sar1b
Aldh5a1
Ran
Usp7
Nup93
Bzw1
Glod4
Hp1bp3
Apex1
Fkbp8
Sumo2
Syncrip
Glb1
Ptges3
Psmc6
Ppih
Lrrc59
Rbm25
Ppp6c
Psmd13
Ssb
Dnaja3
Mat2a
Hnrnpul1
Psme4
Psma6
Sf3b2
Psmb2
Trap1
Akr1b3
Hnrnph3
Hnrnph1
Ddx39b
Psmc5
Hagh
Srsf3
Hnrnpd
Nmral1
Cpsf6
Csad
Ube2v1
U2af1
Adrm
Gart
Ilf3
Atp6v1b2
Hnrnpr
Psmd
Psmc4
Ai314180
Glul
Glo1
Ddx5
Exosc2
Lonp1
Npepps
Galk1
Snrpc
Got1
Pgd
Ogdhl
Myef2
Hnrnpa3
Nudt21
Psmb6
Psma4
Atic
Atp6v0a1
Pfas
Nop56
Gatm
Cstf3
Rbm8a
Nhp2l1
Psma2
Prdx3
Khsrp
Snrpb2
Pa2g4
Ndrg2
Eif4a2
Kars
Eif4a1
Sf3b4
Polr2j
Grpel1
Atp6v1g1
Mars
Etfa
Aldh1l1
Mthfd1l
Mthfd1
Xrn2
Eif2s2
Yars
Adsl
Ddx17
Etf1
Rps3
Txnl1
Ddx6
Naca
Clpp
Tmco1
Adh5
Eif1ax
Rpl5
Nt5c
Dcps
Rps13-ps2
Sars
Acss1
Gstm1
Cnpy2
Eif6
Eif5b
Cell1
Rrp1
Eif2s3x
Itpa
Xpnpep
Gmpr2
Whsc2
Rpl18a
ENSMUSG00000021807
Rps23
Rps2
Hars
Ndufv1
Pccb
Abhd11
Mt1
Ikbkap
Eif1b
Rps16
Rpl7a
Esd
Rpl28
Rpn1
D10Wsu52e
Ppil4
Txndc5
Slc25a5
Sc4mol
Cd81
Pura
Gm7536
Srpr
Metap2
Ndufab1
Ndufs8
Ndufs7
Ivd
Reep5
Mvd
Cds2
Fam98b
Btf3l4
Pdcd5
Ero1l
Fkbp2
Erp29
Tomm7
Prcp
Tm9sf3
Paip2
Pqbp1
Ndufa4
Ndufs5
Idi1
Stt3a
Cbx1
Phpt1
Osgep
Bcas2
Lman2
S100a11
Cbx5
Cox7a2
Tmed9
Galntl1
Cox8a
Zmpste24
Prep
Armc1
Pcyox1l
P4ha1
Fabp5
Rsu1
Ier3ip1
Hsbp1
Zadh2
Pgpep1
Mtch2
Fhl1
Tmsb10
Gng5
Cnpy4
Srgap2
Marcksl1
Slc16a1

B

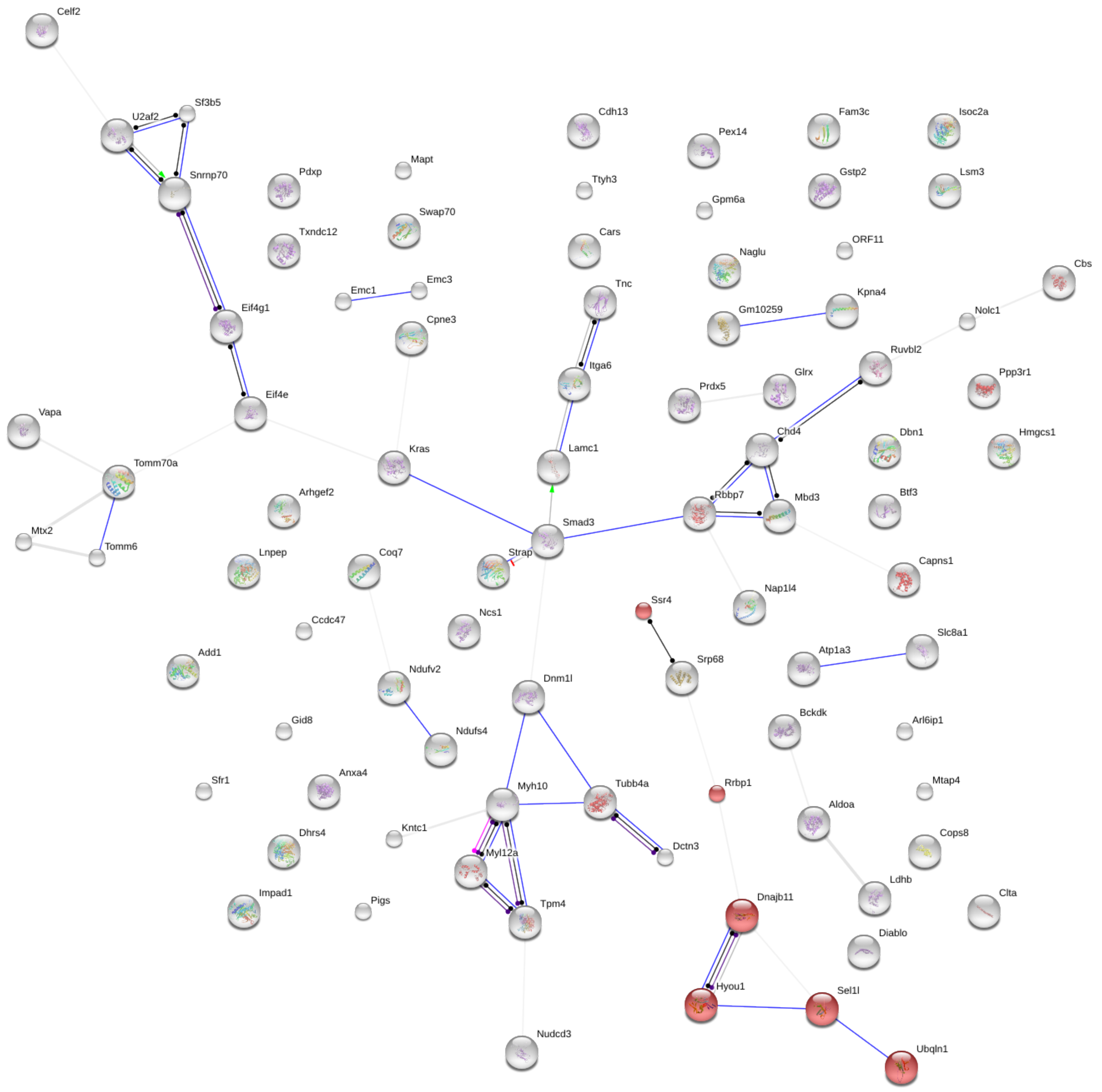
Celf2
U2af2
Sf3b5
Snrnp70
Eif4g1
Eif4e
Pdxp
Txndc12
Mapt
Swap70
Emc1
Emc3
Cpne3
Cdh13
Ttyh3
Cars
Tnc
Itga6
Lamc1
Pex14
Gpm6a
Naglu
Fam3c
Gstp2
ORF11
Isoc2a
Lsm3
Cbs
Gm10259
Kpna4
Nolc1
Ruvbl2
Prdx5
Glrx
Ppp3r1
Chd4
Dbn1
Hmgcs1
Vapa
Tomm70a
Mtx2
Tomm6
Arhgef2
Kras
Smad3
Strap
Rbbp7
Mbd3
Btf3
Capns1
Lnpep
Coq7
Ncs1
Nap1l4
Ssr4
Srp68
Ccdc47
Add1
Ndufv2
Ndufs4
Dnm1l
Atp1a3
Slc8a1
Bckdk
Arl6ip1
Gid8
Sfr1
Anxa4
Myh10
Tubb4a
Dctn3
Rrbp1
Aldoa
Mtap4
Cops8
Kntc1
Dhrs4
Myl12a
Tpm4
Ldhb
Clta
Impad1
Pigs
Dnajb11
Diablo
Hyou1
Sel1l
Ubqln1
Nudcd3

C

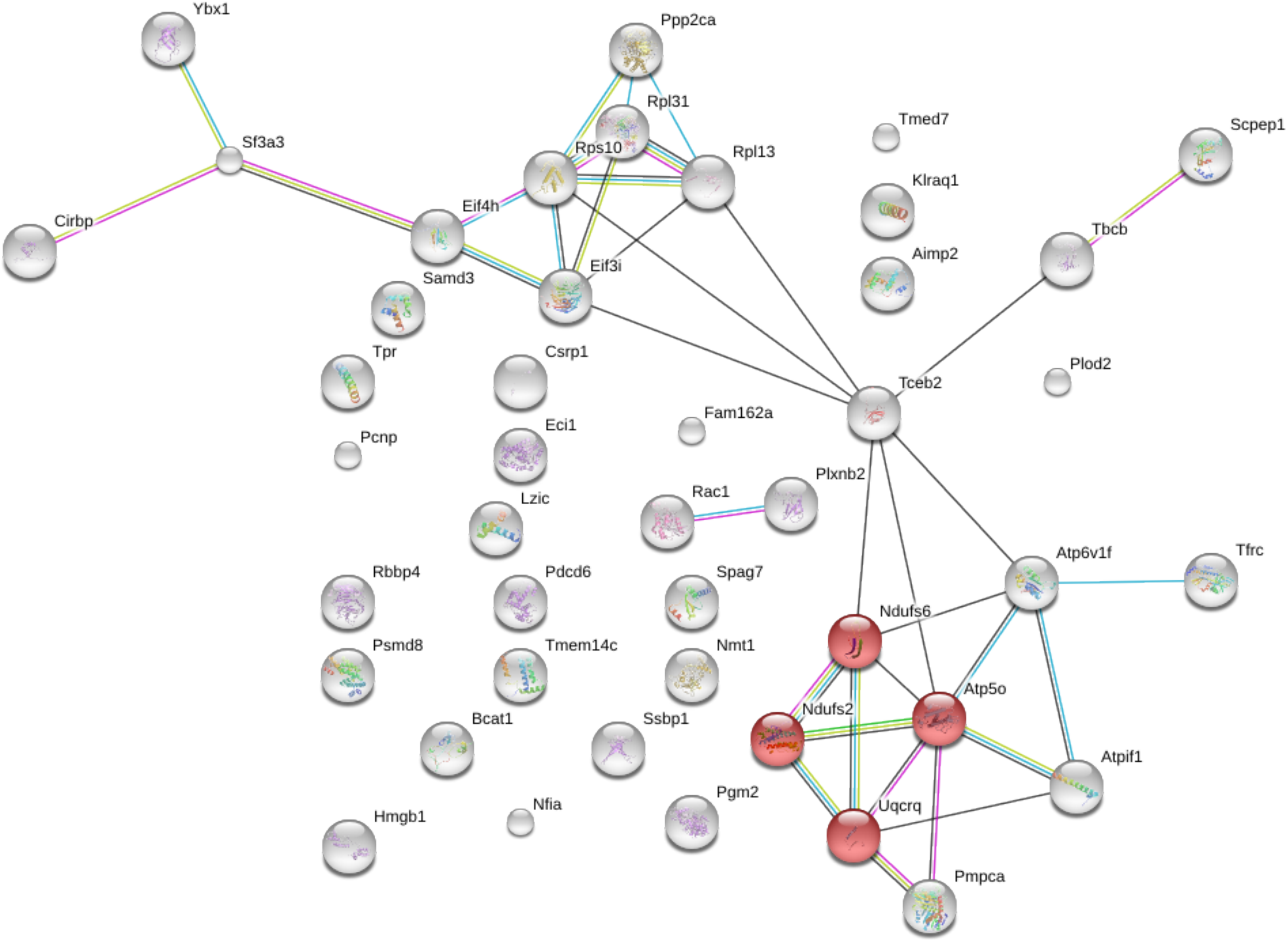
Ybx1
Ppp2ca
Rpl31
Tmed7
Scpep1
Sf3a3
Rps10
Rpl13
Klraq1
Cirbp
Eif4h
Tbcb
Samd3
Eif3i
Aimp2
Tpr
Csrp1
Plod2
Tceb2
Fam162a
Eci1
Pcnp
Plxnb2
Rac1
Lzic
Atp6v1f
Tfrc
Rbbp4
Pdcd6
Spag7
Ndufs6
Psmd8
Tmem14c
Nmt1
Atp5o
Ndufs2
Bcat1
Ssbp1
Atpif1
Uqcrq
Pgm2
Nfia
Hmgb1
Pmpca

Supplementary Figure 1: Proteins and their distributions following 100 nM BTZ and CFZ treatment in mouse E14 embryo-derived NSCS. Listed proteins are detected only in one group following drug treatments. **A.** CONT 24h, proteins shown with red are involved in ubiquitin proteasome system. **B.** BTZ 24h, proteins shown with red are involved in ER pathways and **C.** CFZ 24h, proteins shown with red are involved in oxidative phosphorylation. Figures were created with String v.10.0. Small nodes: protein of unknown 3D structure and large nodes: some 3D structure is known or predicted.

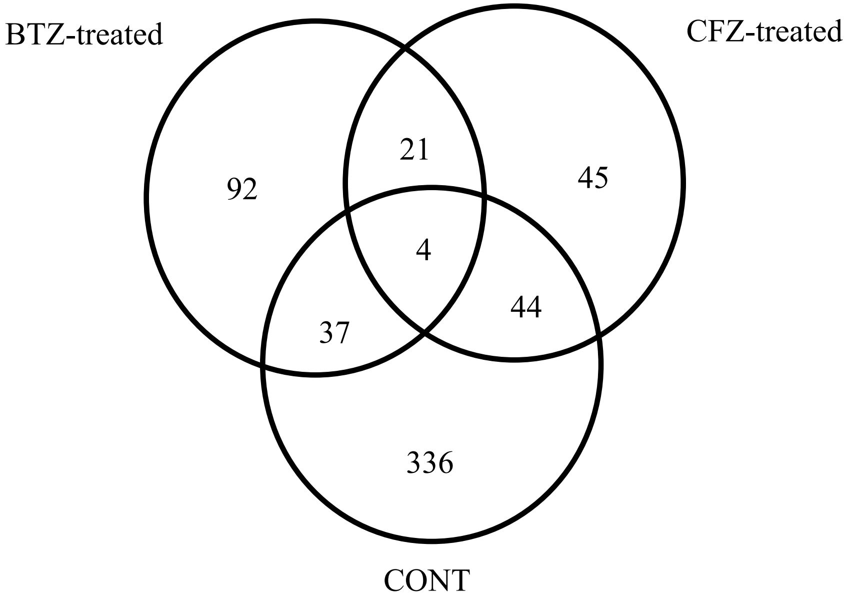

Supplementary Figure 2: Venn diagram indicating the overlap between the differentially expressed proteins identified in three different groups (CONT, BTZ and CFZ).

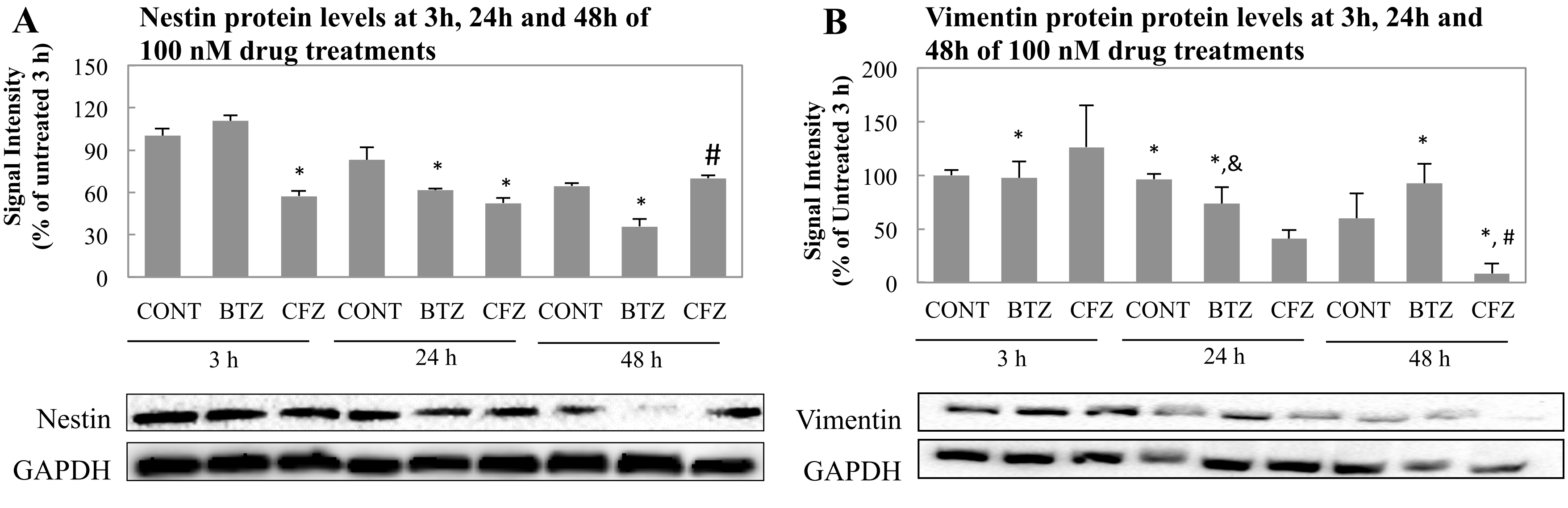

Supplementary Figure 3: Effects on cytoskeletal proteins **A.** Nestin and **B.** Vimentin in mouse NSCs following 3h, 24h and 48h of 100 nM BTZ and CFZ treatments. CONT 3h was set as 100%. Percentage of all lanes was calculated as proportioning the lane density to CONT 3h. Data denote mean ± % S.D. (n=3). * $p<0.05$ vs CONT group of each time point, [&] $p<0.05$ CFZ 24h vs BTZ 24h, [#] $p<0.05$ CFZ 48h vs BTZ 48h. Blots are cropped to improve the clarity. Full-length blots are included in the Supplementary Data.

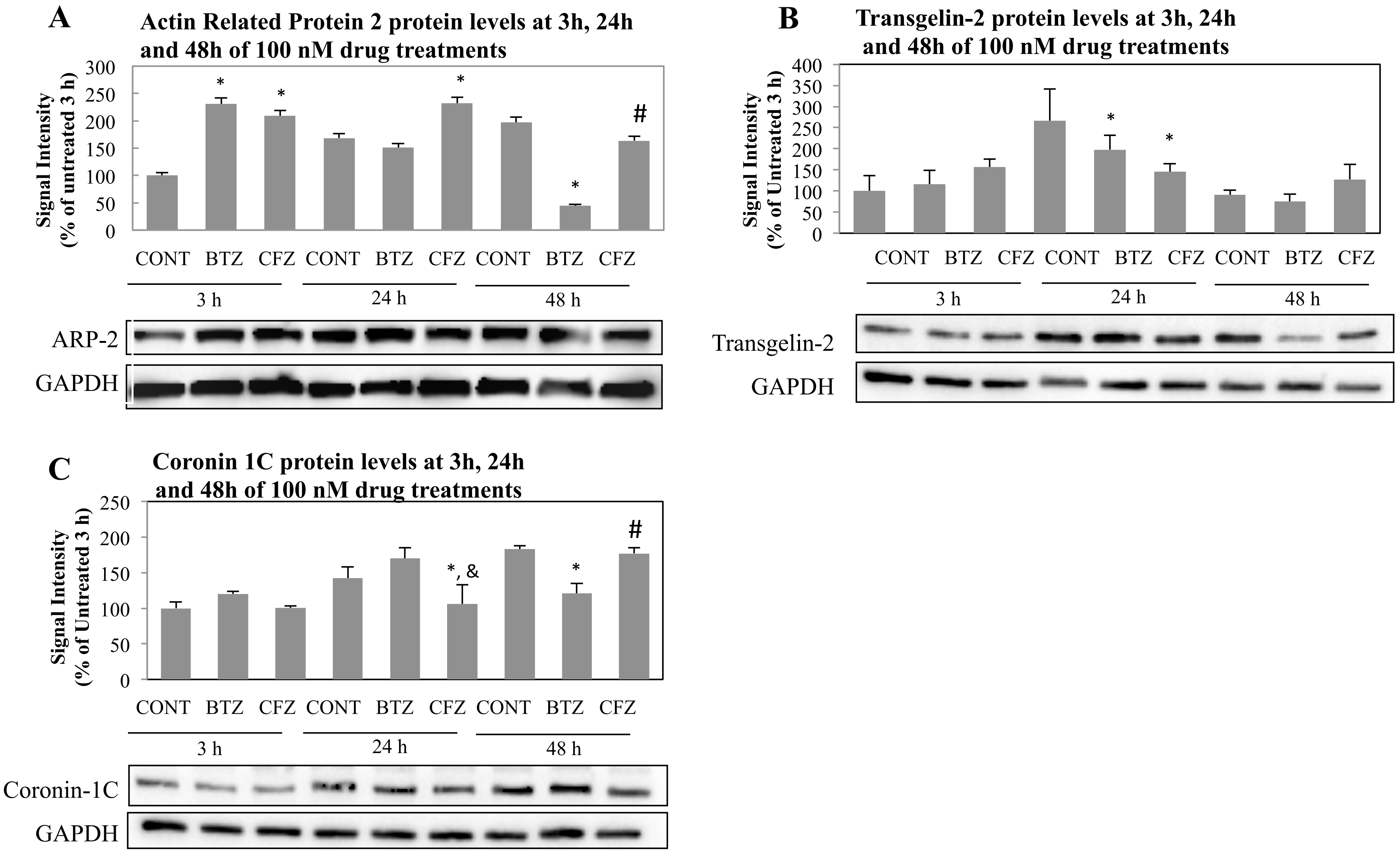

Supplementary Figure 4: Effects on actin related protein-2, transgelin-2 and coronin 1C protein expressions in mouse NSCs following 3h, 24h and 48h of 100 nM BTZ and CFZ treatments. CONT 3h was set as 100%. Percentage of all lanes was calculated as proportioning the lane density to CONT 3h. Data denote mean ± % S.D. (n=3). * $p<0.05$ vs CONT group of each time point, [&] $p<0.05$ CFZ 24h vs BTZ 24h, [#] $p<0.05$ CFZ 48h vs BTZ 48h. Blot is cropped to improve the clarity. Full-length blots are included in the Supplementary Data.

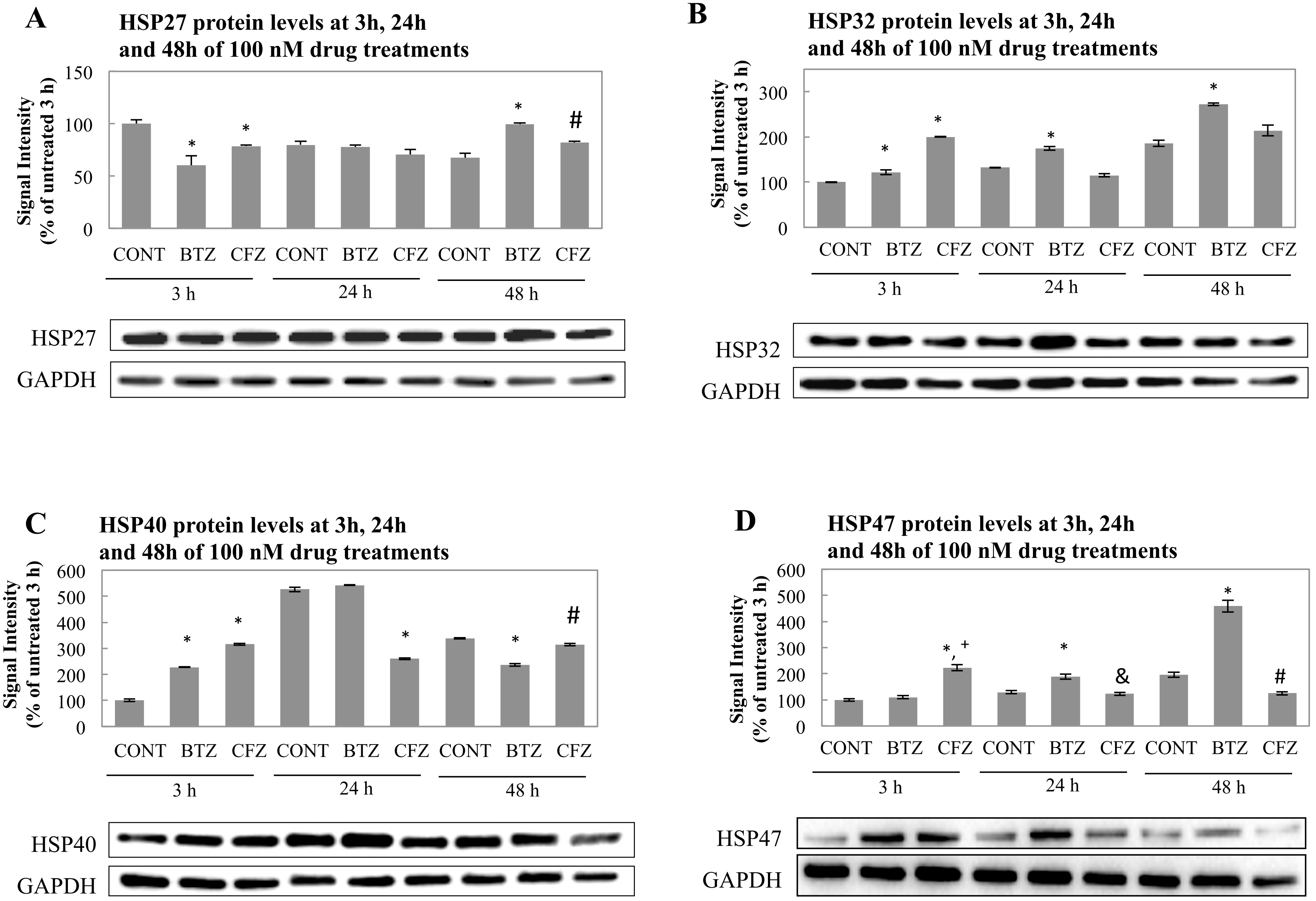

A
HSP27 protein levels at 3h, 24h and 48h of 100 nM drug treatments
Signal Intensity (% of untreated 3 h)
CONT BTZ CFZ CONT BTZ CFZ CONT BTZ CFZ
3 h
24 h
48 h
HSP27
GAPDH
B
HSP32 protein levels at 3h, 24h and 48h of 100 nM drug treatments
HSP32
GAPDH
C
HSP40 protein levels at 3h, 24h and 48h of 100 nM drug treatments
HSP40
GAPDH
D
HSP47 protein levels at 3h, 24h and 48h of 100 nM drug treatments
HSP47
GAPDH

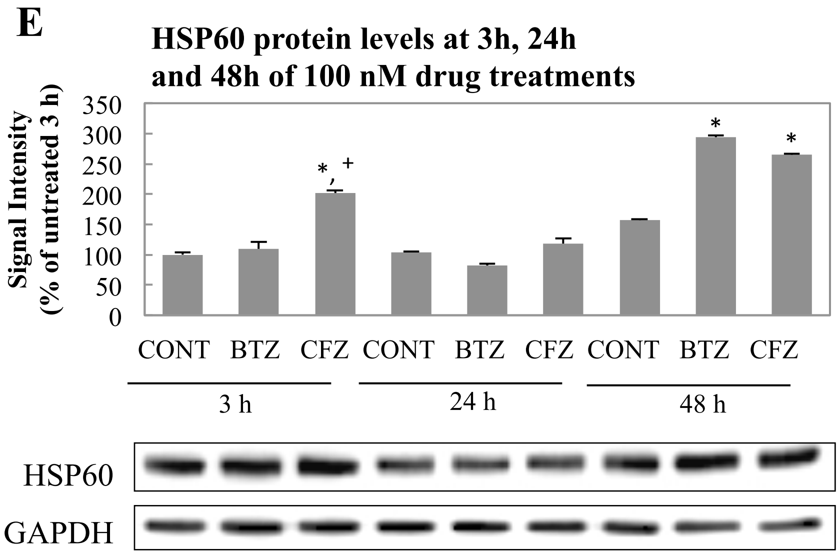

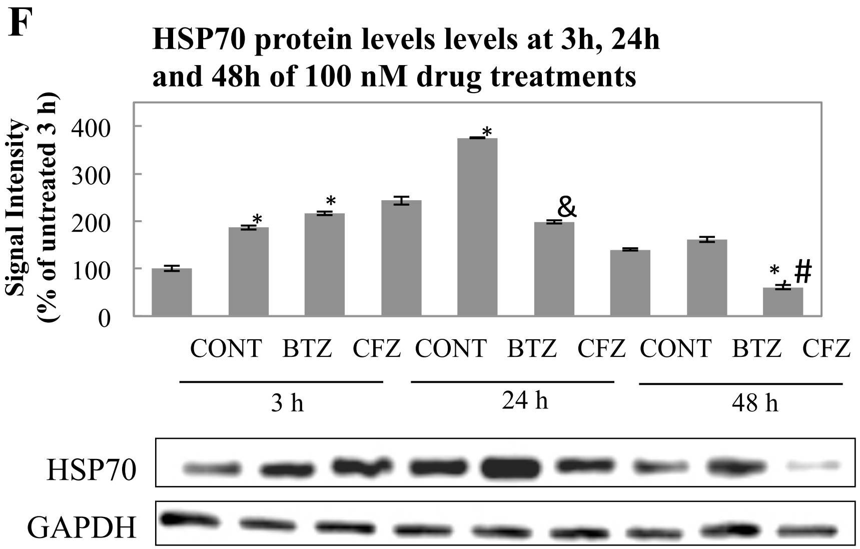

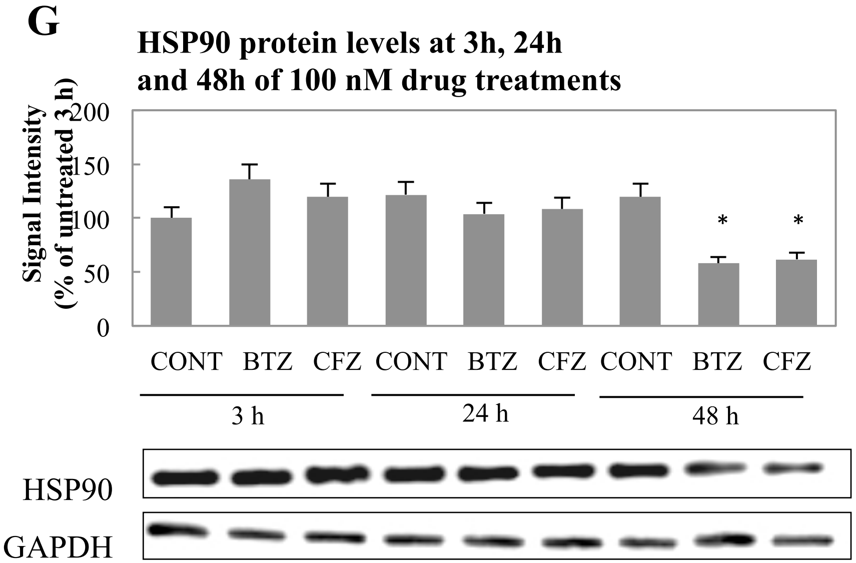

Supplementary Figure 5: Effects on heat shock proteins (HSPs) in mouse NSCs following 3h, 24h and 48h of 100 nM BTZ and CFZ treatments. CONT 3h was set as 100%. Percentage of all lanes was calculated as proportioning the lane density to CONT 3h. Data denote mean ± % S.D. (n=3). * $p<0.05$ vs CONT group of each time point, $^{\&}$ $p<0.05$ CFZ 24h vs BTZ 24h, $^{\#}$ $p<0.05$ CFZ 48h vs BTZ 48h. Blot is cropped to improve the clarity. Full-length blots are included in the Supplementary Data.

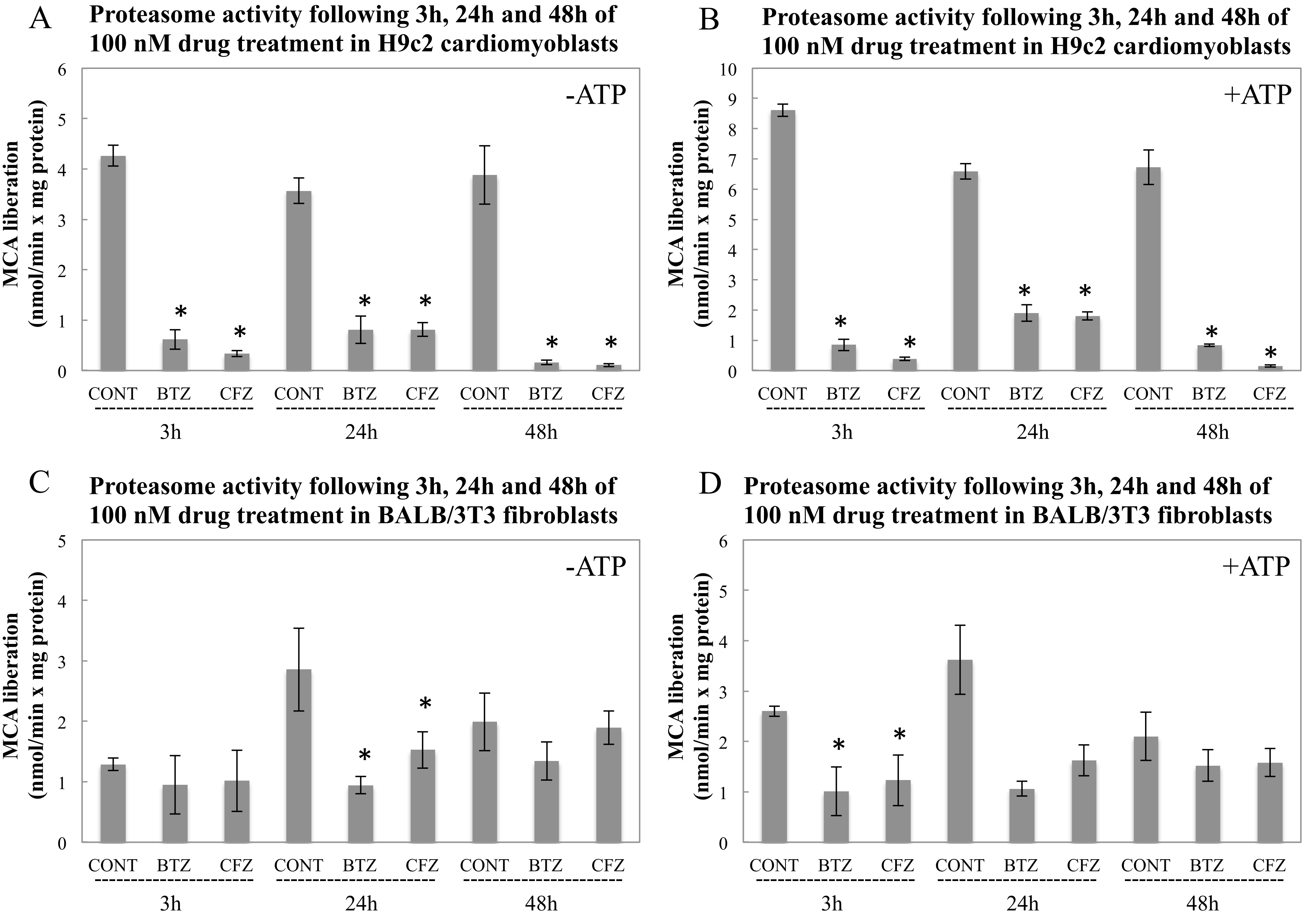
A
Proteasome activity following 3h, 24h and 48h of 100 nM drug treatment in H9c2 cardiomyoblasts
-ATP
MCA liberation (nmol/min x mg protein)
0 1 2 3 4 5 6
CONT BTZ CFZ CONT BTZ CFZ CONT BTZ CFZ
3h 24h 48h
B
Proteasome activity following 3h, 24h and 48h of 100 nM drug treatment in H9c2 cardiomyoblasts
+ATP
MCA liberation (nmol/min x mg protein)
0 1 2 3 4 5 6 7 8 9 10
CONT BTZ CFZ CONT BTZ CFZ CONT BTZ CFZ
3h 24h 48h
C
Proteasome activity following 3h, 24h and 48h of 100 nM drug treatment in BALB/3T3 fibroblasts
-ATP
MCA liberation (nmol/min x mg protein)
0 1 2 3 4 5
CONT BTZ CFZ CONT BTZ CFZ CONT BTZ CFZ
3h 24h 48h
D
Proteasome activity following 3h, 24h and 48h of 100 nM drug treatment in BALB/3T3 fibroblasts
+ATP
MCA liberation (nmol/min x mg protein)
0 1 2 3 4 5 6
CONT BTZ CFZ CONT BTZ CFZ CONT BTZ CFZ
3h 24h 48h

Supplementary Figure 6: Effects on proteasome activity in H9c2 cardiomyoblasts and BALB/3T3 embryo fibroblasts. Data denote mean ± S.D. *$p < 0.05$ vs. CONT (n = 3). Results were evaluated with ANOVA test followed by multiple comparison analysis. (A) represents proteasome activity without ATP addition in H9c2 cells; (B) represents proteasome activity with ATP addition into the reaction mixtures in H9c2 cells; (C) represents proteasome activity without ATP addition in BALB/3T3 cells; (B) represents proteasome activity with ATP addition into the reaction mixtures in BALB/3T3 cells.

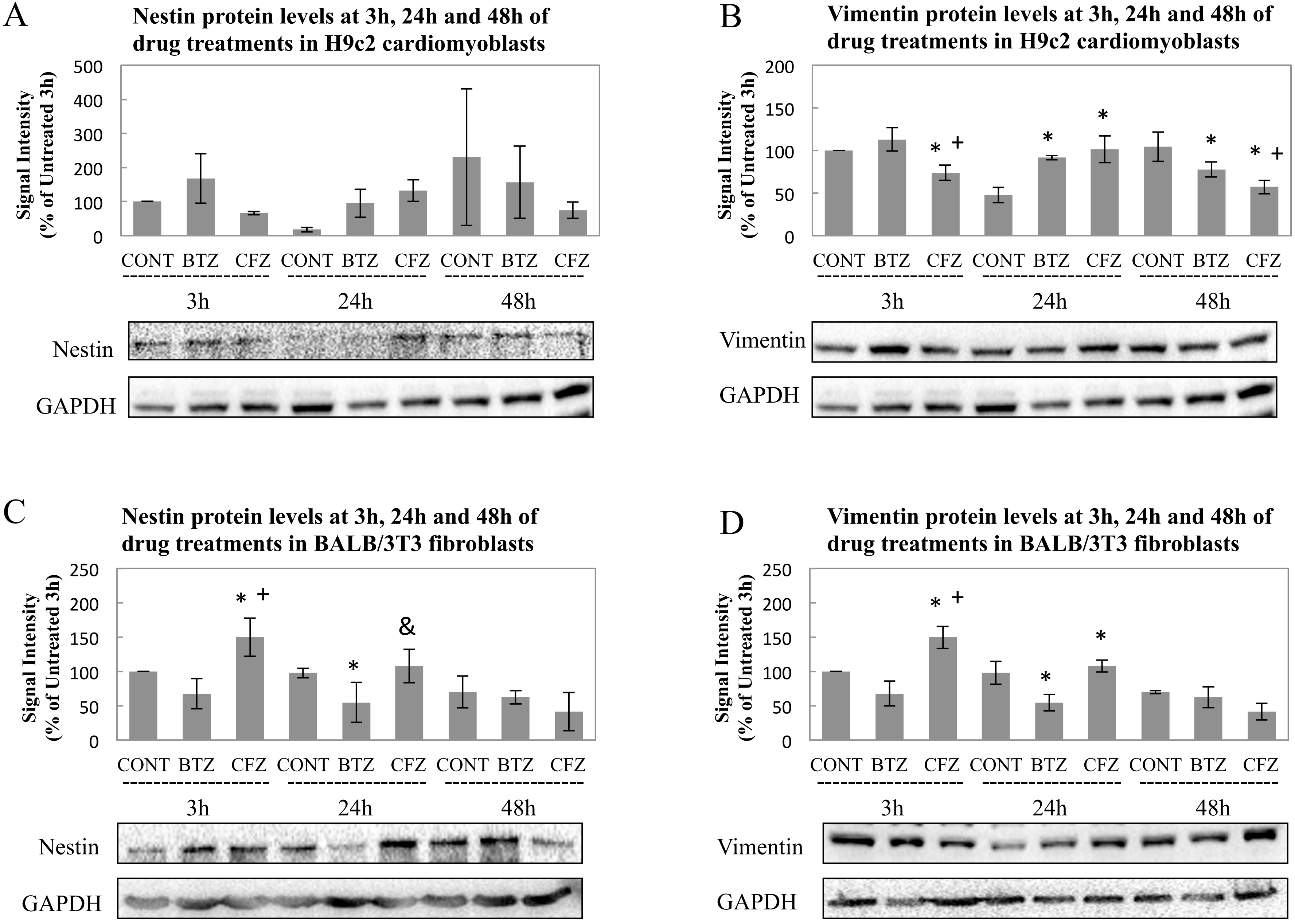
A
Nestin protein levels at 3h, 24h and 48h of drug treatments in H9c2 cardiomyoblasts
Signal Intensity (% of Untreated 3h)
500
400
300
200
100
0
CONT BTZ CFZ CONT BTZ CFZ CONT BTZ CFZ
3h
24h
48h
Nestin
GAPDH
B
Vimentin protein levels at 3h, 24h and 48h of drug treatments in H9c2 cardiomyoblasts
Signal Intensity (% of Untreated 3h)
200
150
100
50
0
* +
*
*
*
* +
CONT BTZ CFZ CONT BTZ CFZ CONT BTZ CFZ
3h
24h
48h
Vimentin
GAPDH
C
Nestin protein levels at 3h, 24h and 48h of drug treatments in BALB/3T3 fibroblasts
Signal Intensity (% of Untreated 3h)
250
200
150
100
50
0
* +
&
*
CONT BTZ CFZ CONT BTZ CFZ CONT BTZ CFZ
3h
24h
48h
Nestin
GAPDH
D
Vimentin protein levels at 3h, 24h and 48h of drug treatments in BALB/3T3 fibroblasts
Signal Intensity (% of Untreated 3h)
250
200
150
100
50
0
* +
*
*
CONT BTZ CFZ CONT BTZ CFZ CONT BTZ CFZ
3h
24h
48h
Vimentin
GAPDH

Supplementary Figure 7: Effects on nestin and vimentin proteins in H9c2 cardiomyoblasts and BALB/3T3 embryo fibroblasts following 3h, 24h and 48 h of 100 nM BTZ and CFZ treatments. **A** and **B.** Nestin expression and Vimentin expression in H9c2 cardiomyoblasts. **C** and **D.** Nestin expression and Vimentin expression in BALB/3T3 embryo fibroblasts. CONT 3h was set to 100%. Percentage of all lanes was calculated as proportioning the lane density to CONT 3h Data denote mean ± % S.D. * $p<0.05$ vs CONT group of each time point, $^{+}p<0.05$ CFZ 3h vs BTZ 3h, $^{\&}p<0.05$ CFZ 24h vs BTZ 24h. Blot is cropped to improve the clarity. Full-length blots are included in the Supplementary Data.

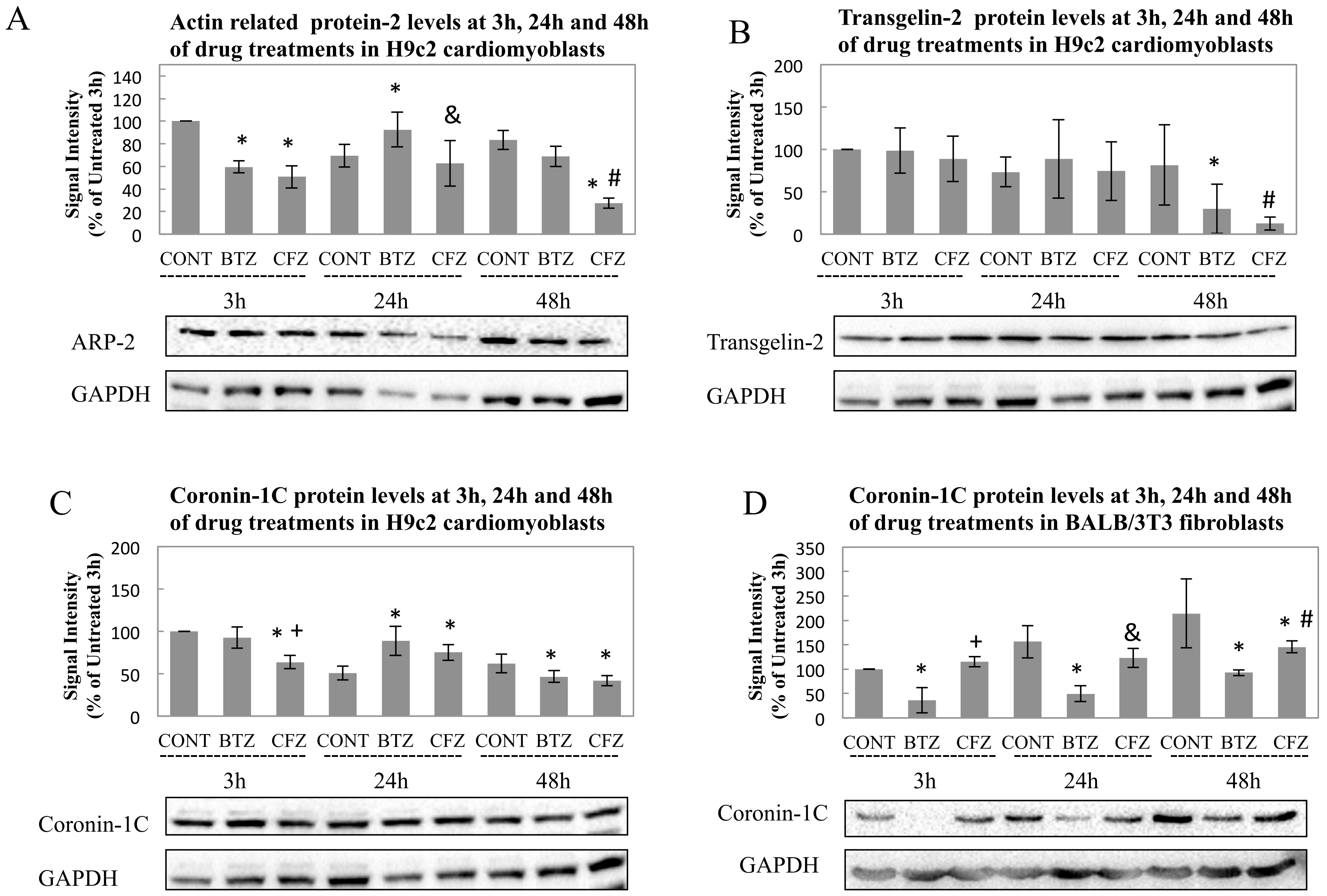
A
Actin related protein-2 levels at 3h, 24h and 48h of drug treatments in H9c2 cardiomyoblasts
Signal Intensity (% of Untreated 3h)
CONT BTZ CFZ CONT BTZ CFZ CONT BTZ CFZ
3h
24h
48h
ARP-2
GAPDH
B
Transgelin-2 protein levels at 3h, 24h and 48h of drug treatments in H9c2 cardiomyoblasts
Transgelin-2
C
Coronin-1C protein levels at 3h, 24h and 48h of drug treatments in H9c2 cardiomyoblasts
Coronin-1C
D
Coronin-1C protein levels at 3h, 24h and 48h of drug treatments in BALB/3T3 fibroblasts

Supplementary Figure 8: Effects on ARP-2, transgelin-2 and coronin-1C proteins in H9c2 cardiomyoblasts and BALB/3T3 embryo fibroblasts following 3h, 24h and 48 h of 100 nM BTZ and CFZ treatments. **A.** ARP-2**, B.** Nestin and **C.** Coronin-1C expressions in H9c2 cardiomyoblasts. **D.** Coronin-1C expressions in BALB/3T3 embryo fibroblasts. CONT 3h was set to 100%. Percentage of all lanes was calculated as proportioning the lane density to CONT 3h Data denote mean ± % S.D. * $p<0.05$ vs CONT group of each time point, $^{+}$ $p<0.05$ CFZ 3h vs BTZ 3h, $^{\&}$ $p<0.05$ CFZ 24h vs BTZ 24h, $^{\#}$ $p<0.05$ CFZ 48h vs BTZ 48h. Blot is cropped to improve the clarity. Full-length blots are included in the Supplementary Data.

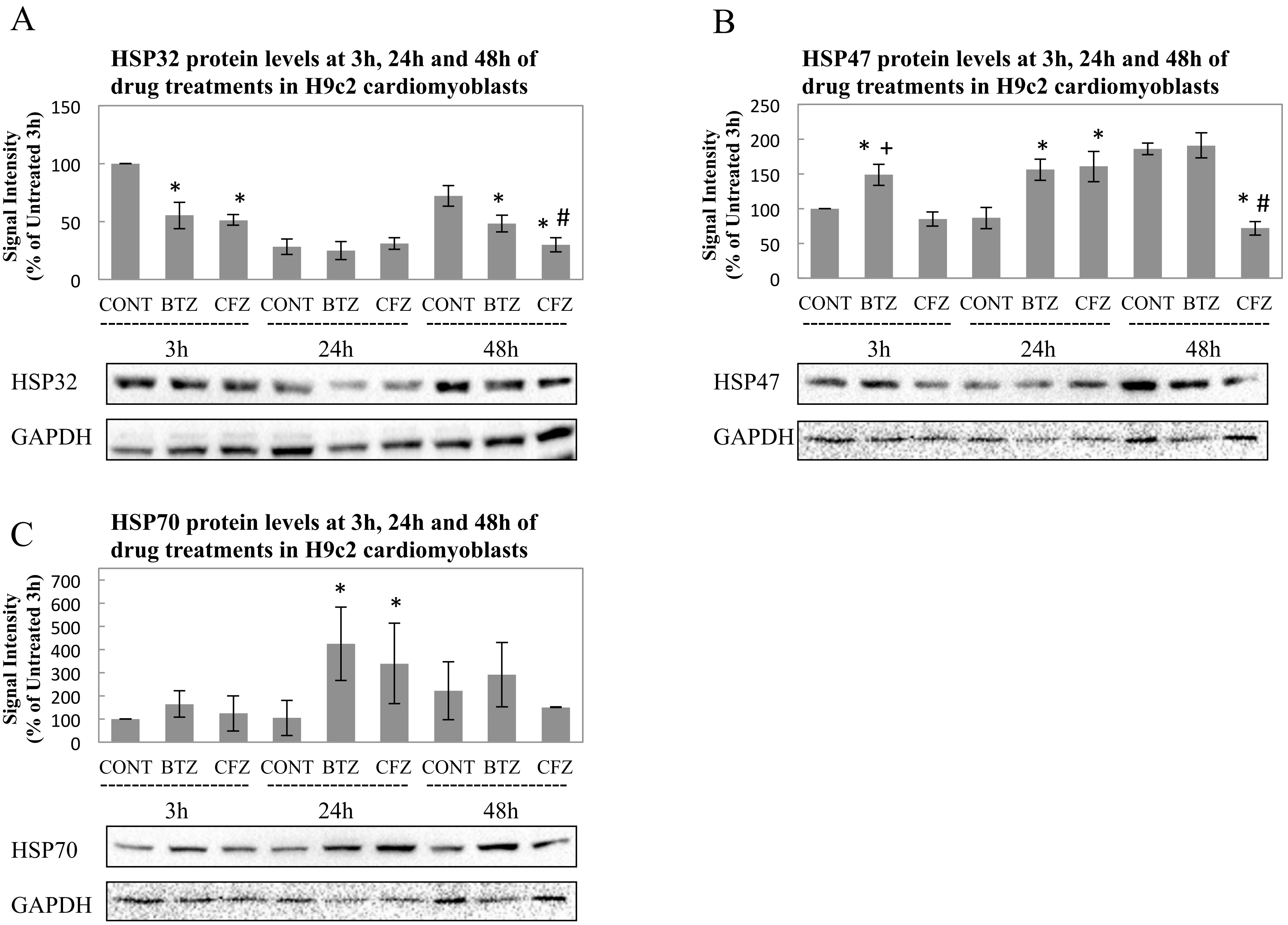
A
HSP32 protein levels at 3h, 24h and 48h of drug treatments in H9c2 cardiomyoblasts
Signal Intensity (% of Untreated 3h)
150
100
50
0
*
*
*
* #
CONT BTZ CFZ CONT BTZ CFZ CONT BTZ CFZ
3h
24h
48h
HSP32
GAPDH
B
HSP47 protein levels at 3h, 24h and 48h of drug treatments in H9c2 cardiomyoblasts
Signal Intensity (% of Untreated 3h)
250
200
150
100
50
0
* +
*
*
* #
CONT BTZ CFZ CONT BTZ CFZ CONT BTZ CFZ
3h
24h
48h
HSP47
GAPDH
C
HSP70 protein levels at 3h, 24h and 48h of drug treatments in H9c2 cardiomyoblasts
Signal Intensity (% of Untreated 3h)
700
600
500
400
300
200
100
0
*
*
CONT BTZ CFZ CONT BTZ CFZ CONT BTZ CFZ
3h
24h
48h
HSP70
GAPDH

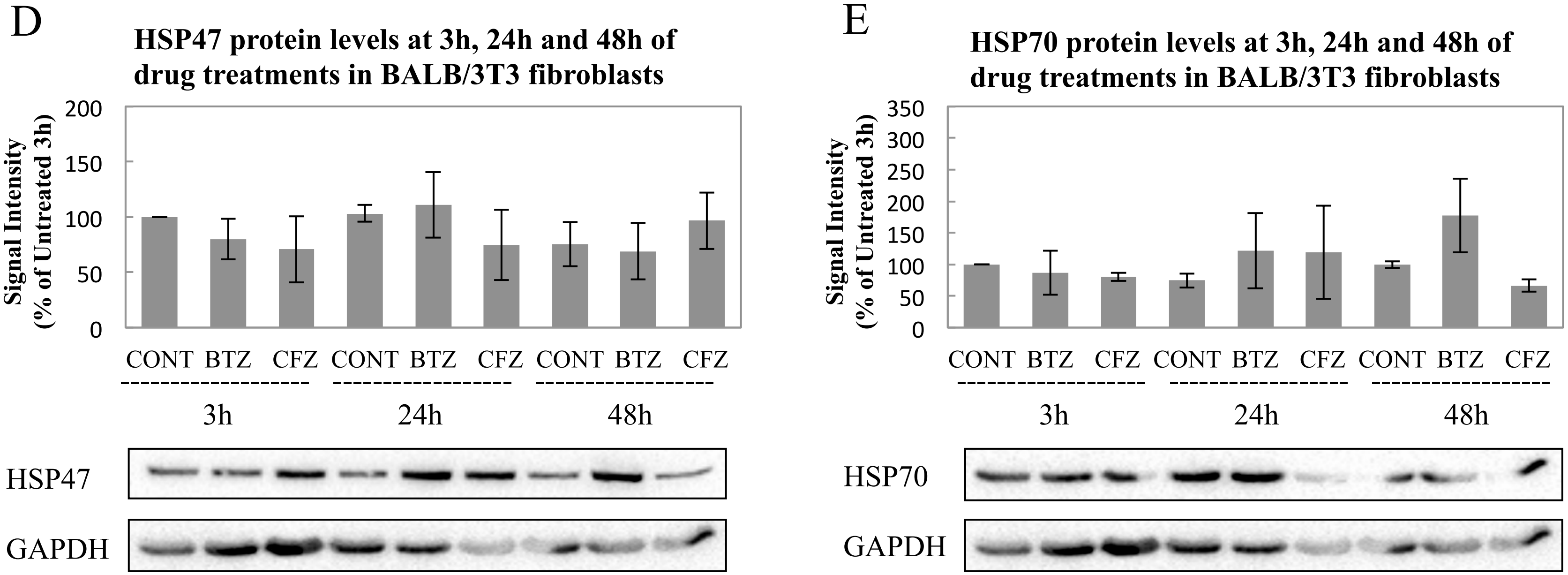

Supplementary Figure 9: Effects on heat shock proteins (HSPs) in H9c2 cardiomyoblasts and BALB/3T3 embryo fibroblasts following 3h, 24h and 48 h of 100 nM BTZ and CFZ treatments. **A.** HSP32**, B.** HSP47 and C. HSP70 expressions in H9c2 cardiomyoblasts. **D.** HSP47, **E.** HSP70 expressions in BALB/3T3 embryo fibroblasts. CONT 3h was set to 100%. Percentage of all lanes was calculated as proportioning the lane density to CONT 3h Data denote mean ± % S.D. * p<0.05 vs CONT group of each time point, $^{+}$ p<0.05 CFZ 3h vs BTZ 3h, $^{\&}$ p<0.05 CFZ 24h vs BTZ 24h, $^{\#}$ p<0.05 CFZ 48h vs BTZ 48h. Blot is cropped to improve the clarity. Full-length blots are included in the Supplementary Data.

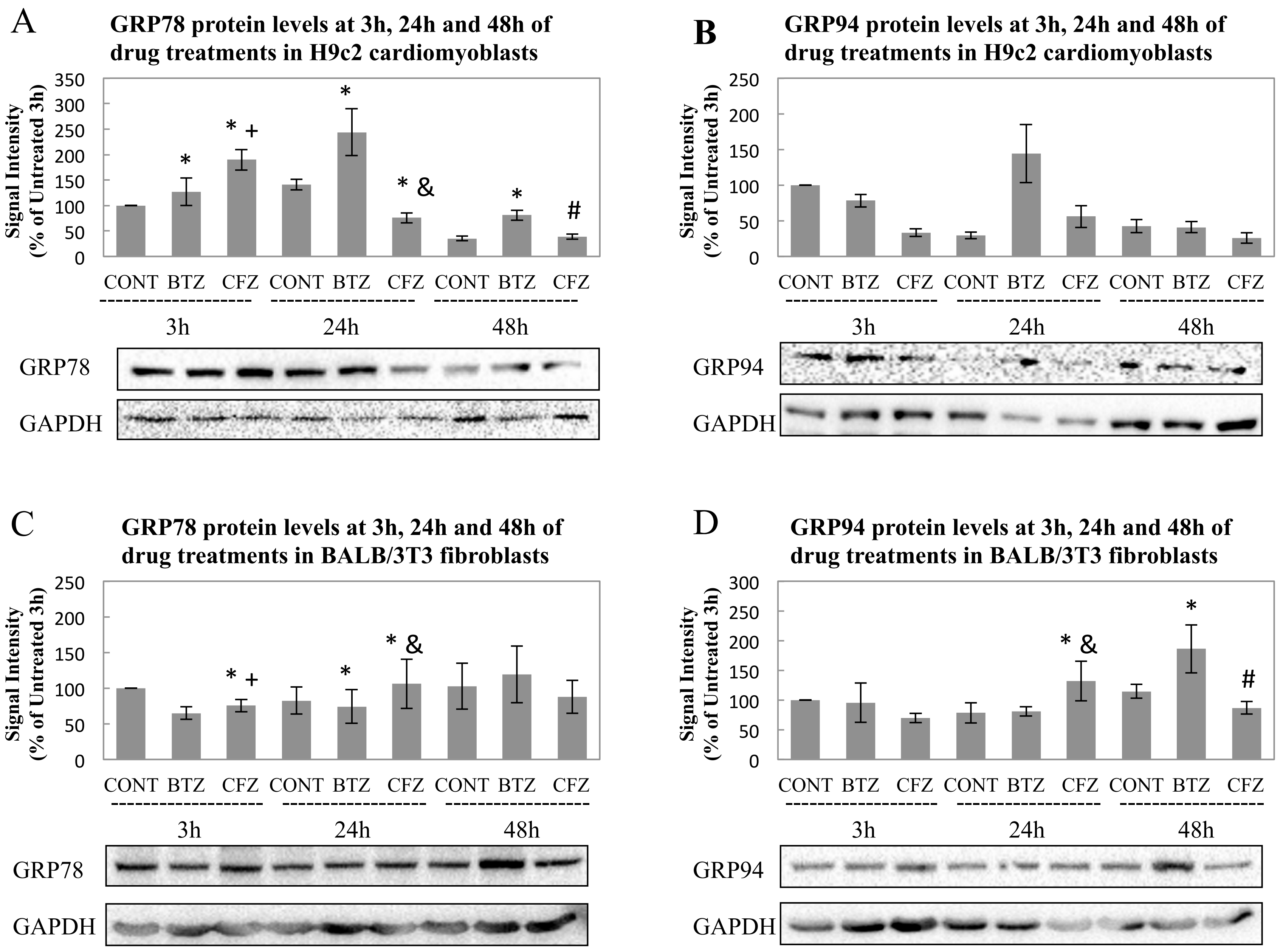

A
GRP78 protein levels at 3h, 24h and 48h of drug treatments in H9c2 cardiomyoblasts
Signal Intensity (% of Untreated 3h)
CONT BTZ CFZ CONT BTZ CFZ CONT BTZ CFZ
3h 24h 48h
GRP78
GAPDH
B
GRP94 protein levels at 3h, 24h and 48h of drug treatments in H9c2 cardiomyoblasts
Signal Intensity (% of Untreated 3h)
CONT BTZ CFZ CONT BTZ CFZ CONT BTZ CFZ
3h 24h 48h
GRP94
GAPDH
C
GRP78 protein levels at 3h, 24h and 48h of drug treatments in BALB/3T3 fibroblasts
Signal Intensity (% of Untreated 3h)
CONT BTZ CFZ CONT BTZ CFZ CONT BTZ CFZ
3h 24h 48h
GRP78
GAPDH
D
GRP94 protein levels at 3h, 24h and 48h of drug treatments in BALB/3T3 fibroblasts
Signal Intensity (% of Untreated 3h)
CONT BTZ CFZ CONT BTZ CFZ CONT BTZ CFZ
3h 24h 48h
GRP94
GAPDH

Supplementary Figure 10: Effects on GRP78 and GRP94 in H9c2 cardiomyoblasts and BALB/3T3 embryo fibroblasts following 3h, 24h and 48 h of 100 nM BTZ and CFZ treatments. **A.** GRP78**, B.** GRP94 expressions in H9c2 cardiomyoblasts. **C.** GRP78, **D.** GRP94 expressions in BALB/3T3 embryo fibroblasts. CONT 3h was set to 100%. Percentage of all lanes was calculated as proportioning the lane density to CONT 3h Data denote mean ± % S.D. * $p<0.05$ vs CONT group of each time point, $^{+}$ $p<0.05$ CFZ 3h vs BTZ 3h, $^{\&}$ $p<0.05$ CFZ 24h vs BTZ 24h, $^{\#}$ $p<0.05$ CFZ 48h vs BTZ 48h. Blot is cropped to improve the clarity. Full-length blots are included in the Supplementary Data.

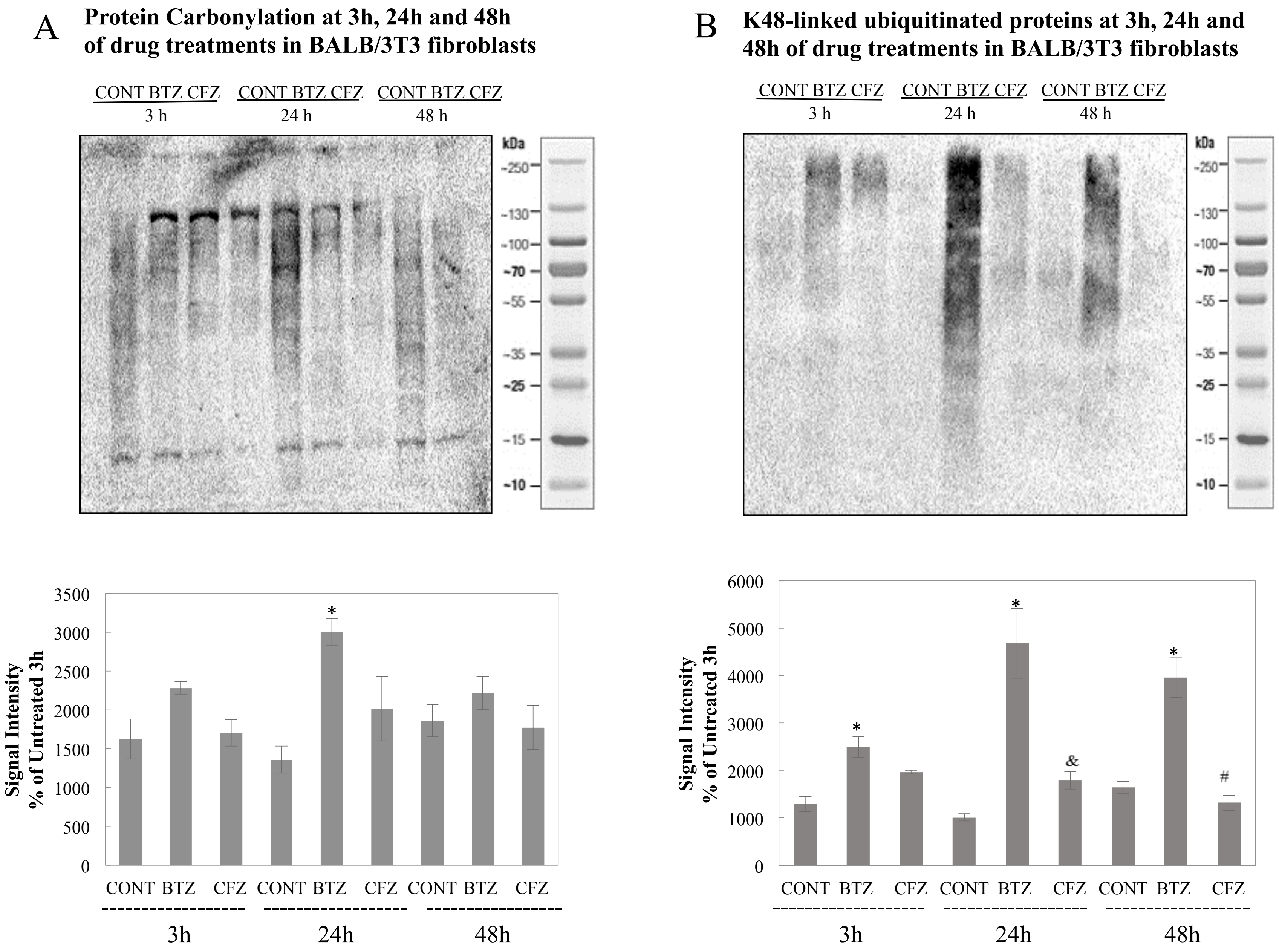
A
Protein Carbonylation at 3h, 24h and 48h of drug treatments in BALB/3T3 fibroblasts
CONT BTZ CFZ
3 h
CONT BTZ CFZ
24 h
CONT BTZ CFZ
48 h
kDa
250
130
100
70
55
35
25
15
10
Signal Intensity
% of Untreated 3h
3500
3000
2500
2000
1500
1000
500
0
*
CONT BTZ CFZ CONT BTZ CFZ CONT BTZ CFZ
3h
24h
48h
B
K48-linked ubiquitinated proteins at 3h, 24h and 48h of drug treatments in BALB/3T3 fibroblasts
CONT BTZ CFZ
3 h
CONT BTZ CFZ
24 h
CONT BTZ CFZ
48 h
kDa
250
130
100
70
55
35
25
15
10
Signal Intensity
% of Untreated 3h
6000
5000
4000
3000
2000
1000
0
*
*
&
*
#
CONT BTZ CFZ CONT BTZ CFZ CONT BTZ CFZ
3h
24h
48h

Supplementary Figure 11: Impact of BTZ and CFZ on protein carbonylation and accumulation of K48-linked ubiquitinated proteins in BALB/3T3 embryo fibroblasts. Cells were treated with BTZ and CFZ; cell lysates were prepared and analyzed via immunoblotting as described in methods. Blots represent one from three replicates (n=3). **A.** shows protein carbonyls following 3h, 24h and 48h treatment; **B.** shows K48-linked ubiquitinated-proteins following 3h, 24h and 48h treatment. Blot is cropped to improve the clarity. Full-length blots are included in the Supplementary Data. Data denote mean ± % S.D. $^{*}p < 0.05$ vs. CONT group, $^{\&}p < 0.05$ vs. BTZ group, $^{\#}p<0.05$ CFZ 48h vs BTZ 48h.

A

Protein Carbonylation at 3h, 24h and 48h of drug treatments in H9c2 cardiomyoblasts

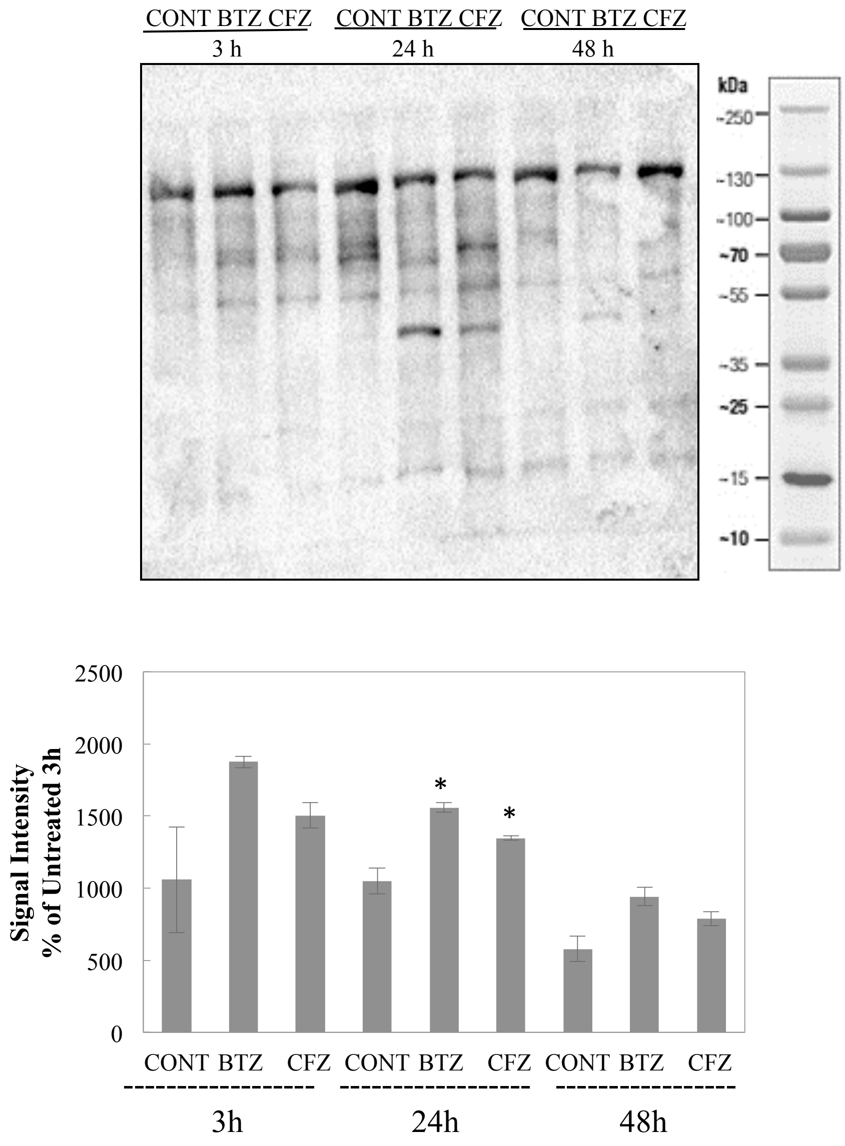

B

K48-linked ubiquitinated proteins at 3h, 24h and 48h of drug treatments in H9c2 cardiomyoblasts

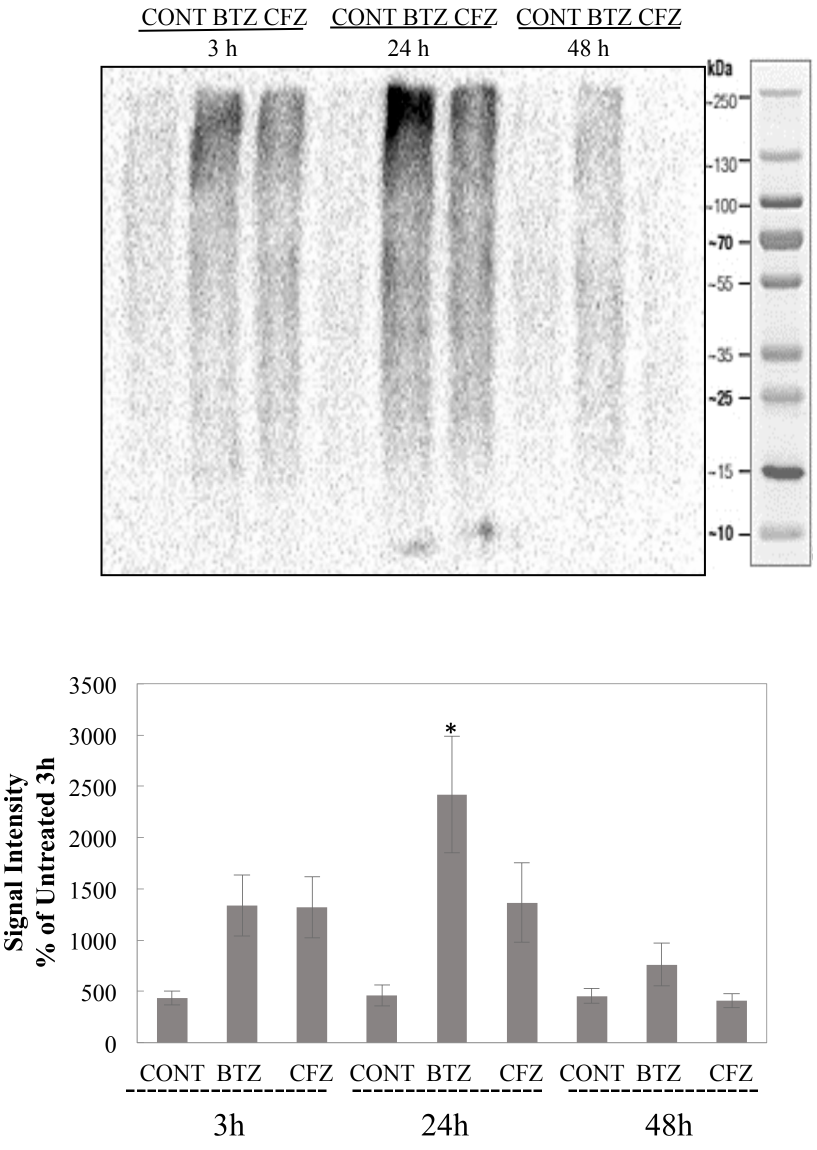

Supplementary Figure 12: Impact of BTZ and CFZ on protein carbonylation and accumulation of K48-linked ubiquitinated proteins in H9c2 cardiomyoblasts. Cells were treated with BTZ and CFZ; cell lysates were prepared and analyzed via immunoblotting as described in methods. Blots represent one from three replicates (n=3). **A.** shows protein carbonyls following 3h, 24h and 48h treatment; **B.** shows K48-linked ubiquitinated-proteins following 3h, 24h and 48h treatment. Blot is cropped to improve the clarity. Full-length blots are included in the Supplementary Data. Data denote mean ± % S.D. $*p < 0.05$ vs. CONT group.

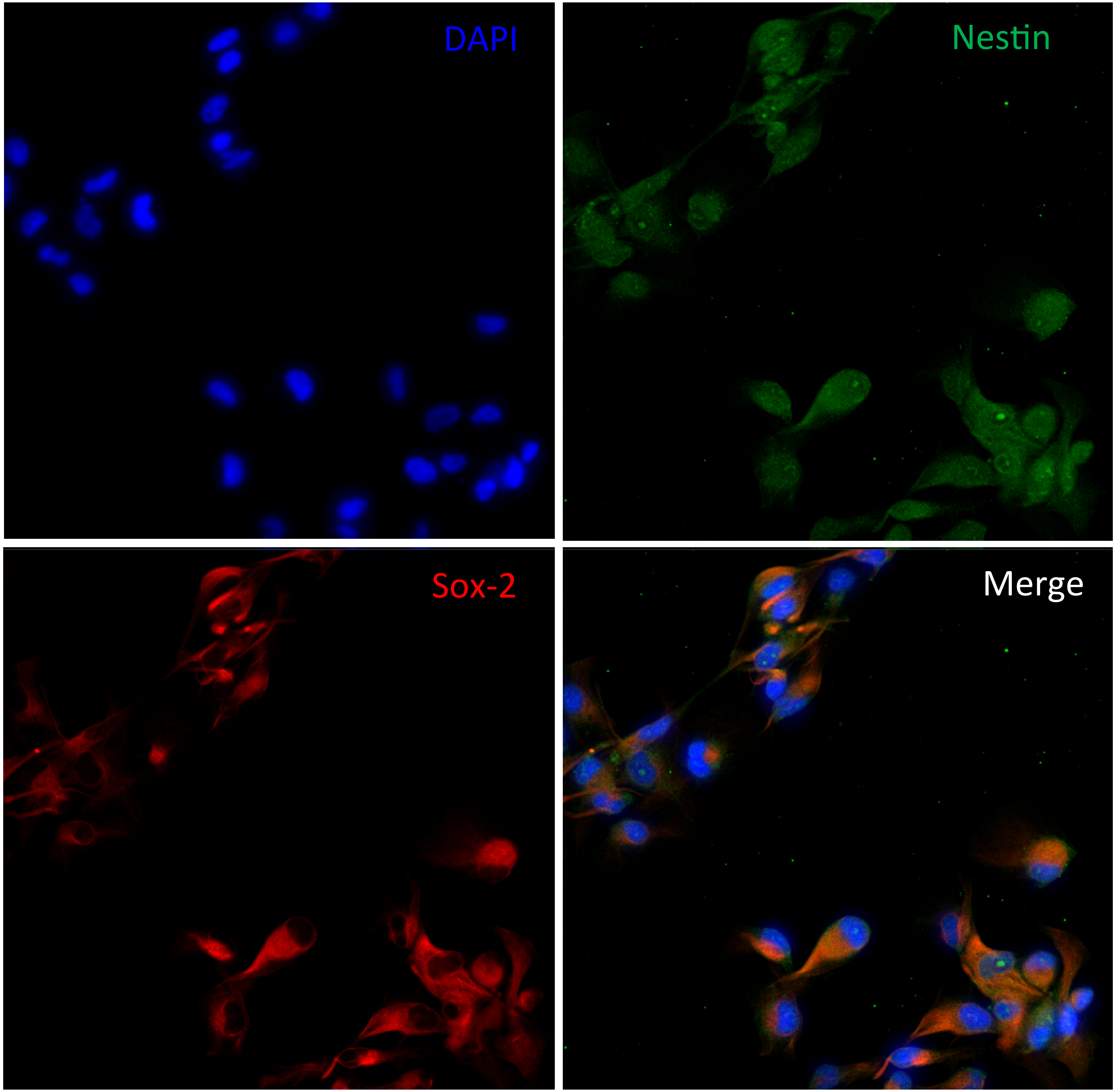

Supplementary Figure 13: Characterization of NSCs in the presence of EGF and FGF-2. Images respresent one of the replicate from three replicates (n=3).

Uncropped blots for the western blots

NSCs

Uncropped blot for Nestin (Figure 3A and Figure S3A)

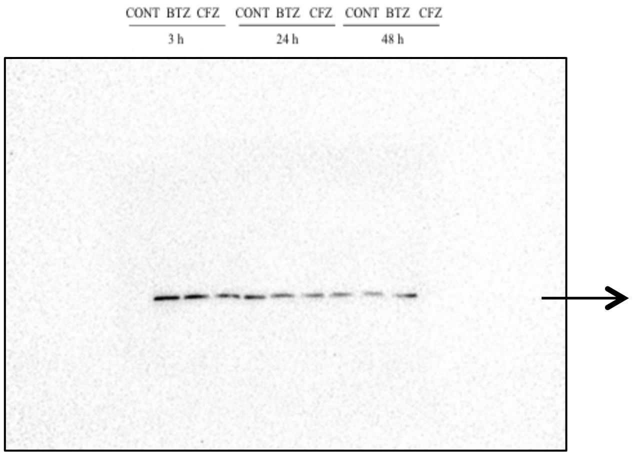

Nestin 170-200 kDa

Abcam Rat Anti-Nestin antibody (clone ab6142)

Uncropped blot for GAPDH (Figure 3A, Figure S3A, Figure 3C, Figure S4A)

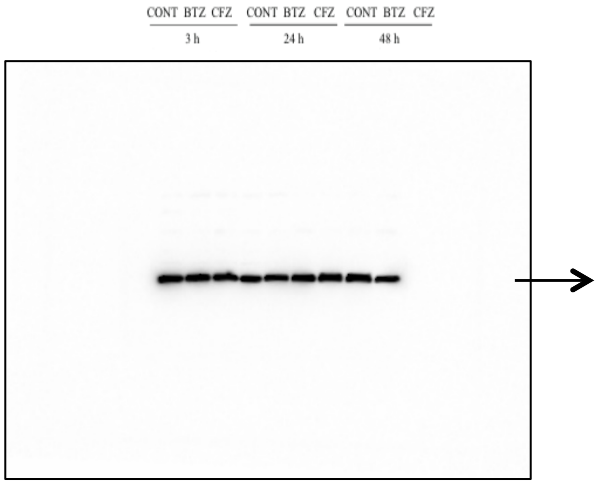

GAPDH 37 kDa

Abcam Anti-GAPDH antibody (clone 8245)

Uncropped blot for ARP2 (Figure 3C, Figure S4A)

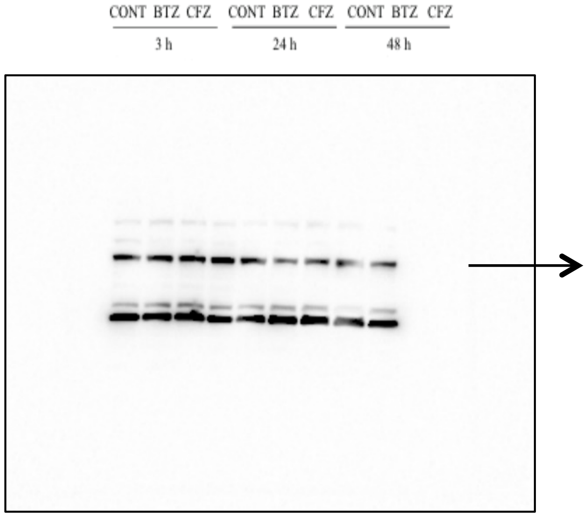

ARP2 44 kDa

CST Anti-ARP2 antibody (clone D85D5)

Membrane for Nesitn, ARP2 and GAPDH (Figure 3A, Figure S3A, Figure 3C, Figure S4A)

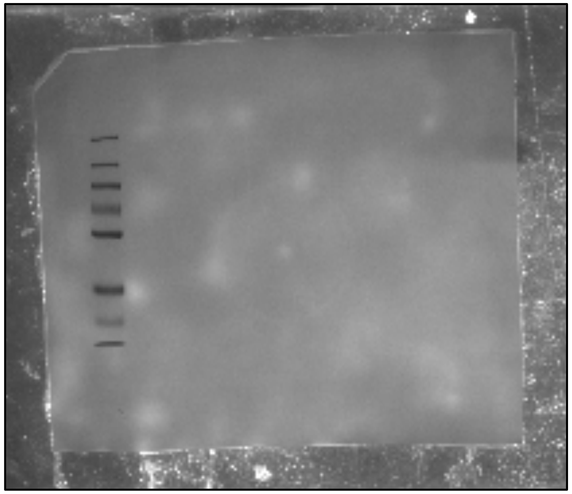

Uncropped blot for Vimentin (Figure 3B, Figure S3B)

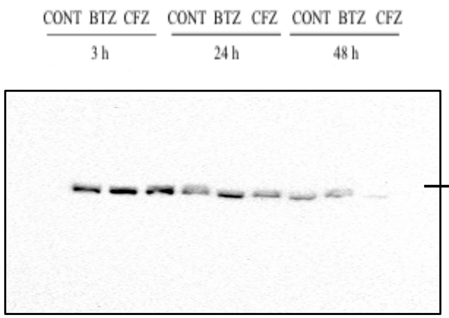

Vimentin 57.50 kDa

CST Anti-Vimentin Antibody (clone 3932)

Uncropped blot for GAPDH (Figure 3B, Figure S3B)

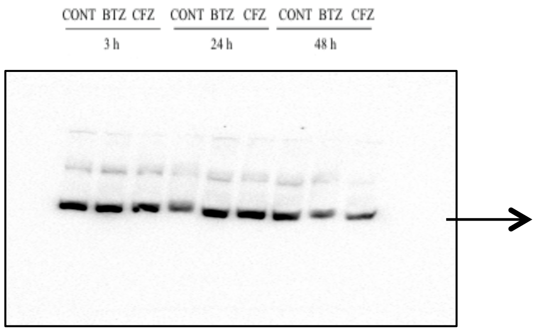

GAPDH 37 kDa

Abcam Anti-GAPDH antibody (clone 8245)

Membrane for Vimentin (Figure 3B, Figure S3B)

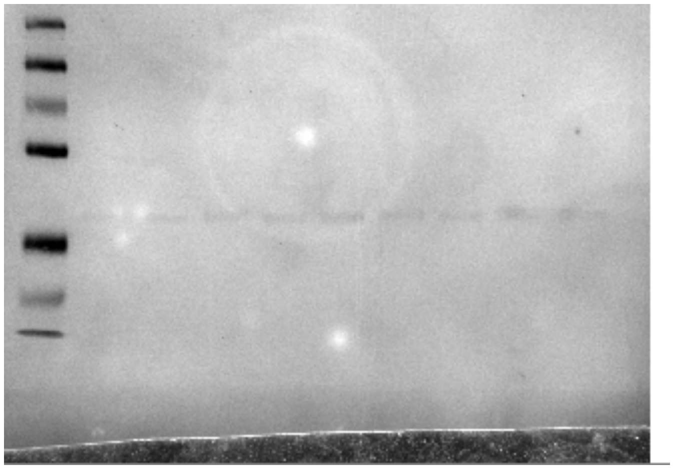

Uncropped blot for Coronin 1C (Figure 3E, Figure S4C)

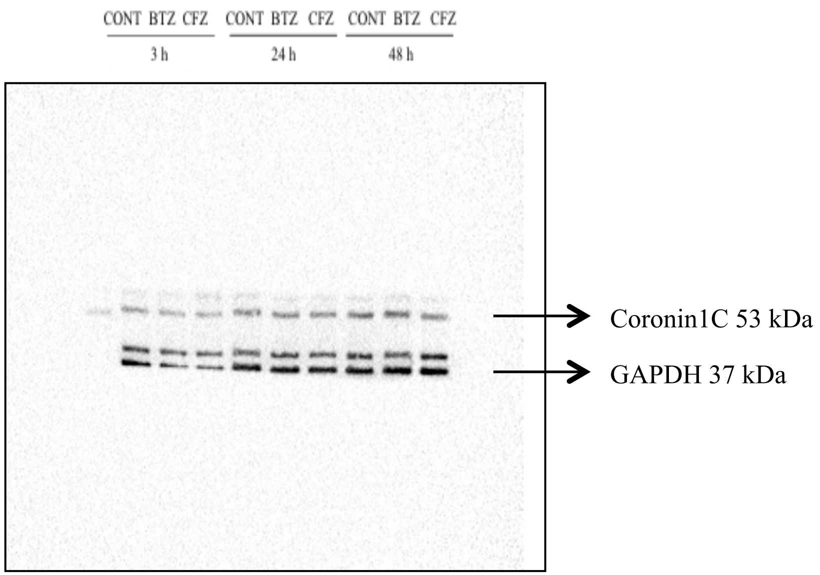

Abcam Anti-Coronin 1C Antibody (clone ab153954)

Uncropped blot for GAPDH (Figure 3E, Figure S4C)

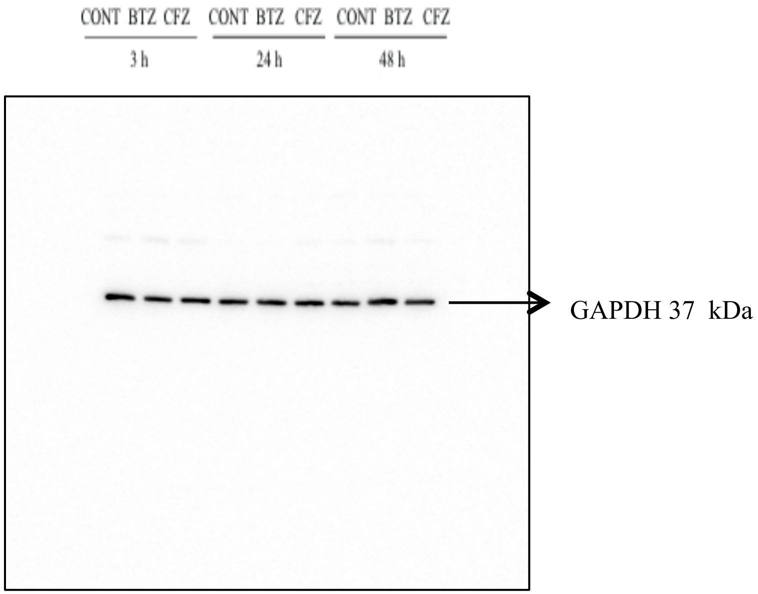

Abcam Anti-GAPDH antibody (clone 8245)

Membranes for Coronin 1C and GAPDH (Figure 3E, Figure S4C)

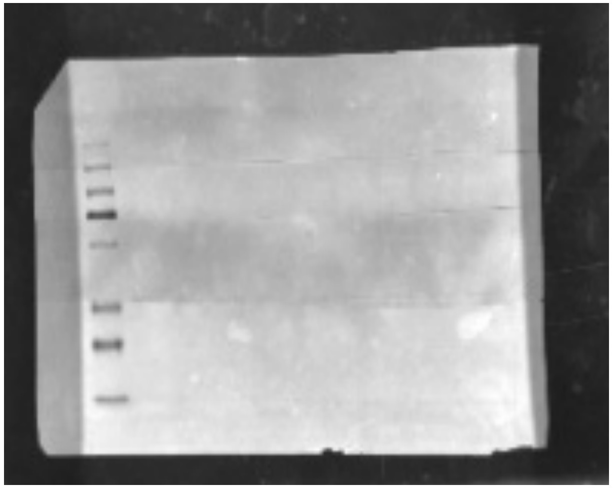

Uncropped blot for Transgelin-2 (Figure 3D, Figure S4B)

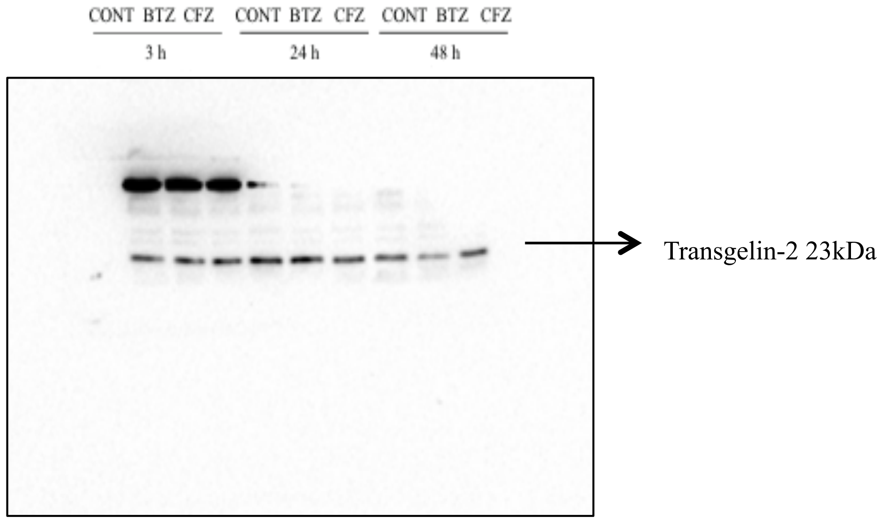

Abcam Anti-SM22 antibody (Anti-Transgelin-2)
(clone ab184522)

Uncropped blot for GAPDH (Figure 3D, Figure S4B)

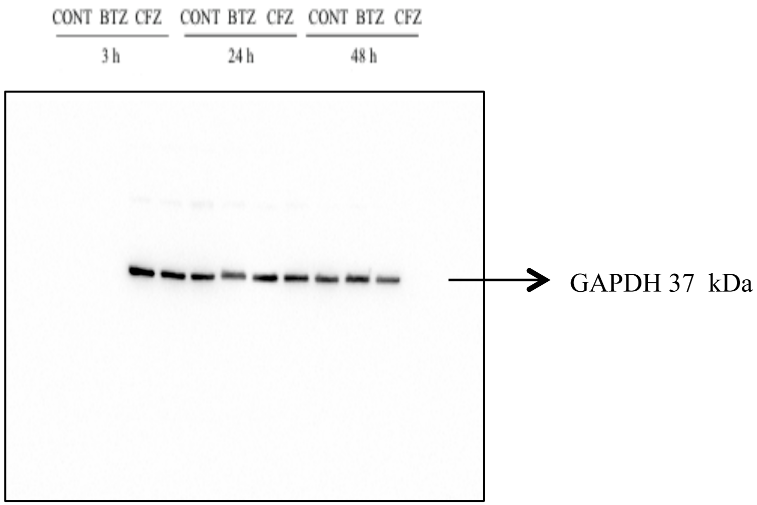

Abcam Anti-GAPDH antibody (clone 8245)

Membranes for Transgelin-2 and GAPDH (Figure 3D, Figure S4B)

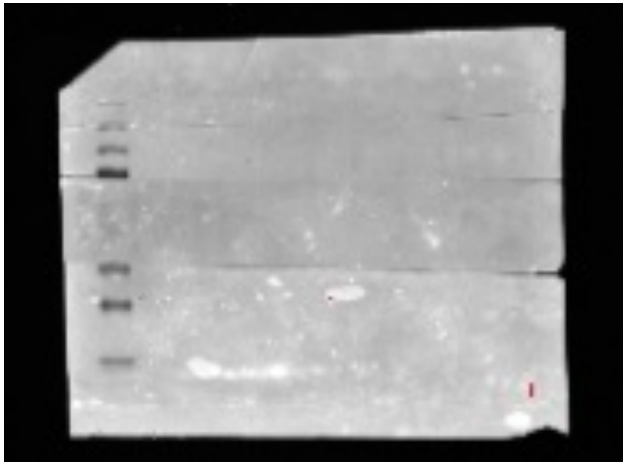

Uncropped blot for HSP32 (Figure 4A, Figure S5B)

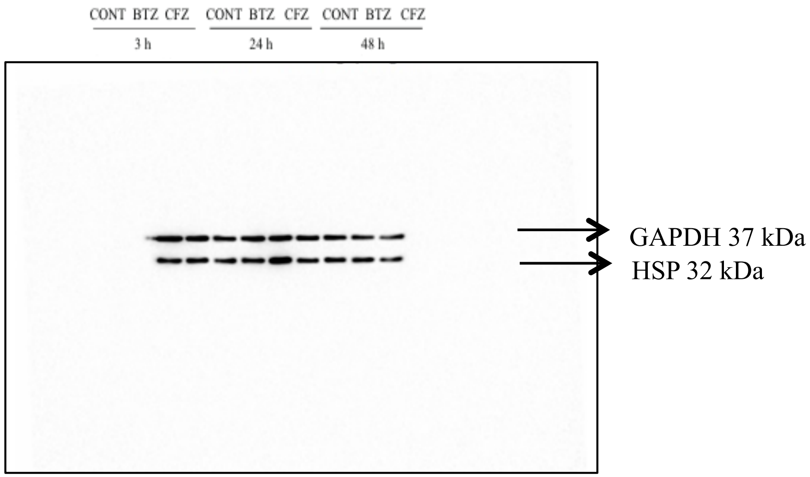

Enzo Anti-HO-1 (HO-1-1, anti-HP32)

Uncropped blot for HSP47 and GAPDH (Figure 4B, Figure S5D)

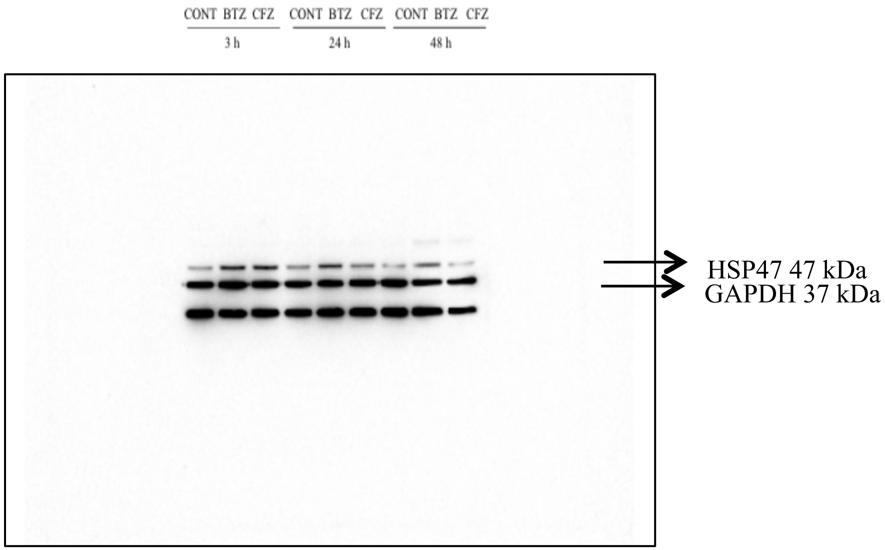

Abcam Anti-HSP47 Antibody (clone ab77609)

Membrane for HSP47, HSP32 and GAPDH (Figure 4A, Figure 4B, Figure S5B, Figure S5D)

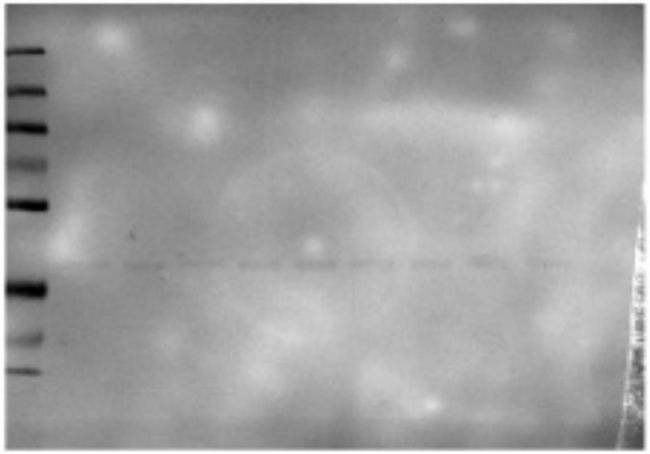

Uncropped blot for HSP70 (Figure 4C, Figure S5F)

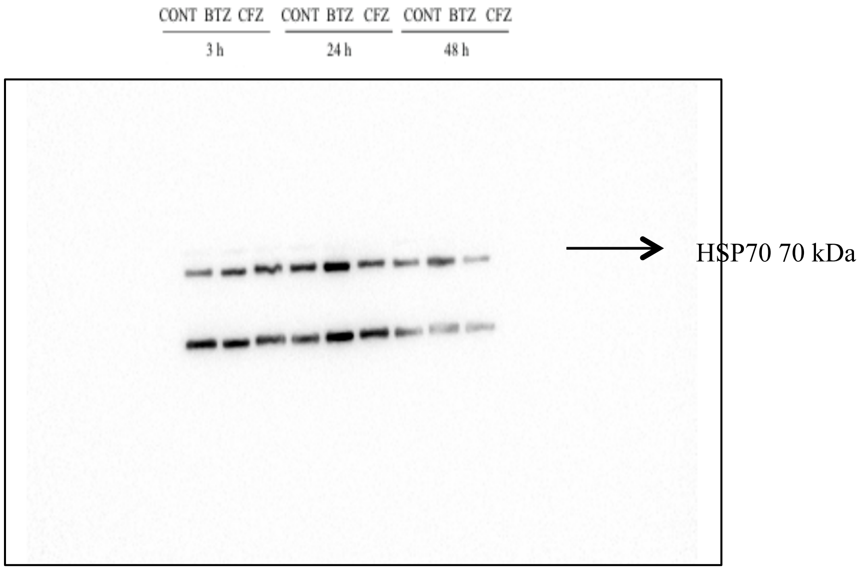

CST Anti-HSP70 Antibody (clone D69)

Uncropped blot for GAPDH (Figure 4C, Figure S5F and Figure S5G)

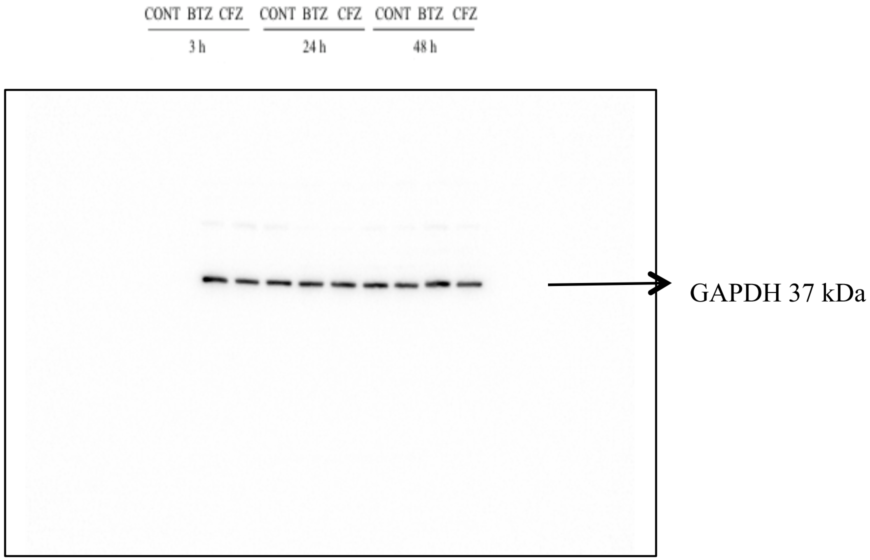

Abcam Anti-GAPDH antibody (clone 8245)

Uncropped blot for HSP90 (Figure S5G)

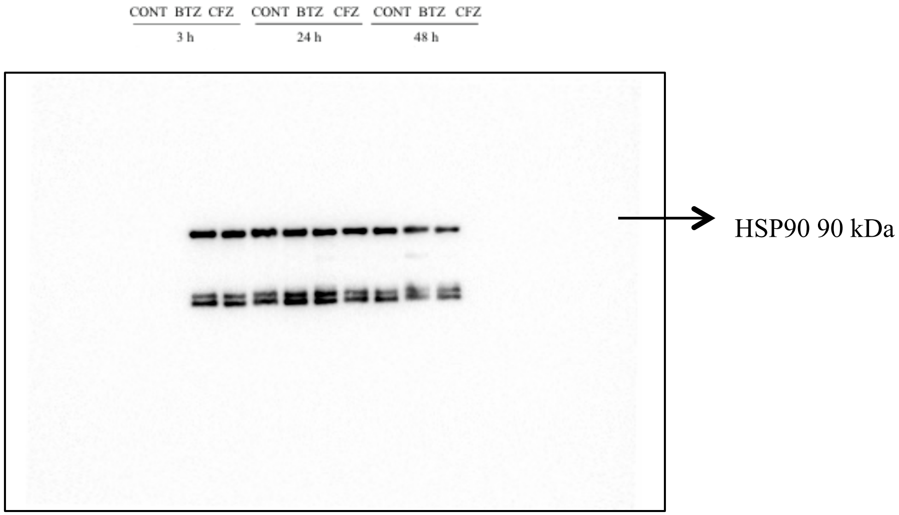

CST Anti-HSP90 Antibody (clone C45G5)

Membrane for HSP70, HSP90 and GAPDH (Figure 4C, Figure S5F and Figure S5G)

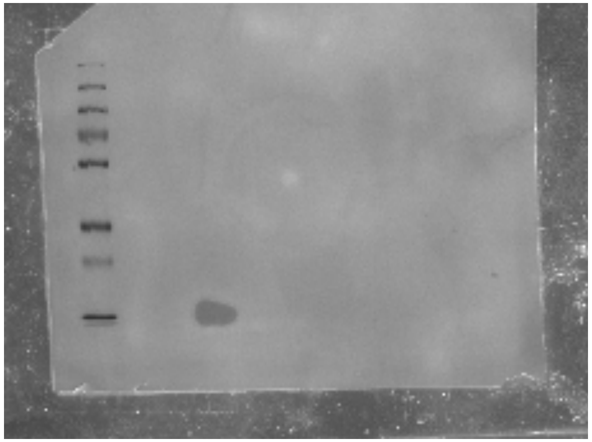

Uncropped blot for GRP94 and GRP78 (Figure 4D and Figure 4E)

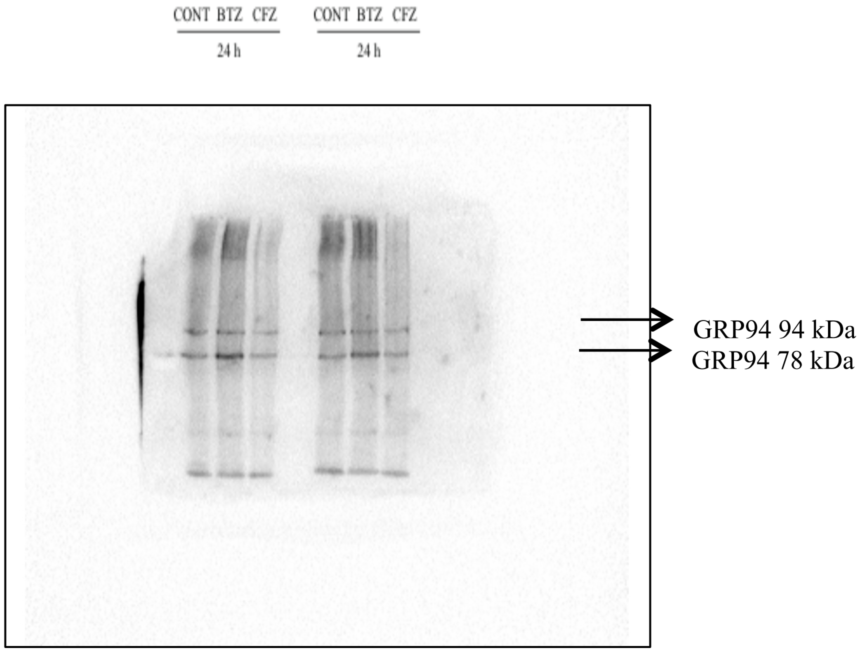

CST Anti-Grp94 Antibody (clone 2104)
CST Anti-BiP Antibody (clone C50B12)

Membrane for GRP94 and GRP78 (Figure 4D, Figure 4E)

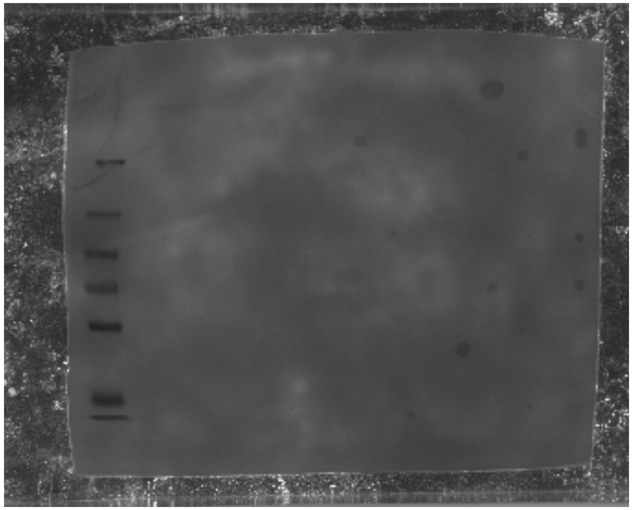

Uncropped blot for Co-precipitated B-actin by HSP70 (Figure 5A)

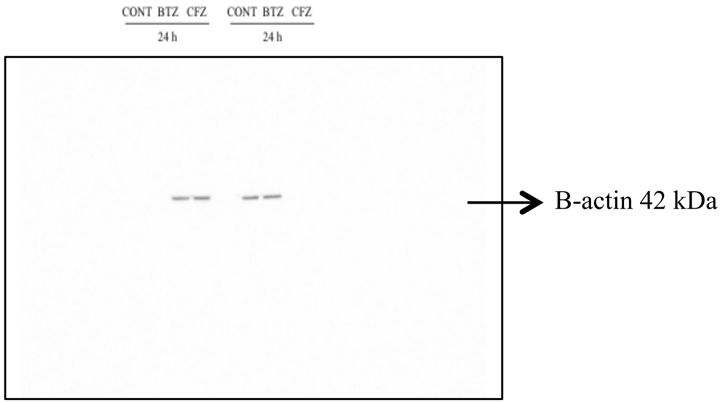

Abcam Anti-beta Actin Antibody (clone ab8227)

Membrane for B-actin (Figure 5A)

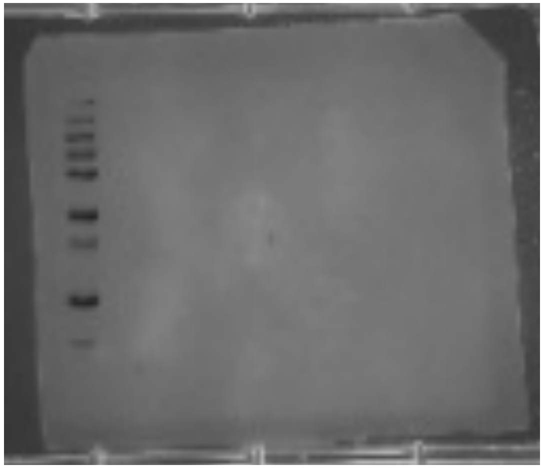

Uncropped blot for Co-precipitated HSP70 by B-actin (Figure 5B)

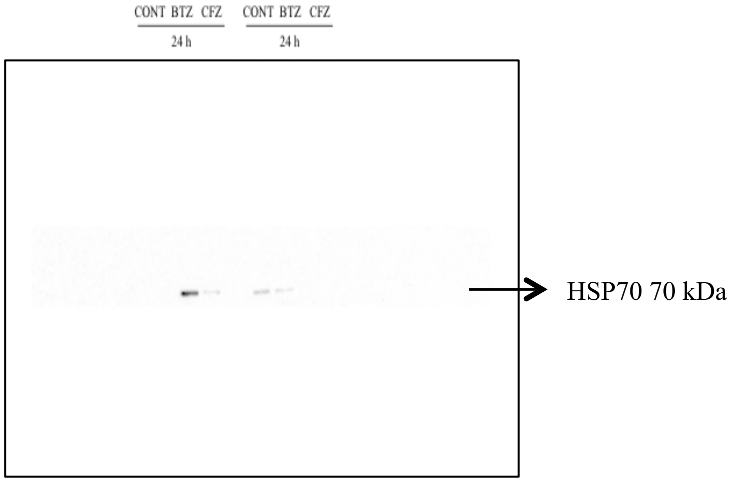

Thermo Scientific Inc. Anti-HSP70 Antibody (clone 5A5)

Membrane for HSP70 (Figure 5B)

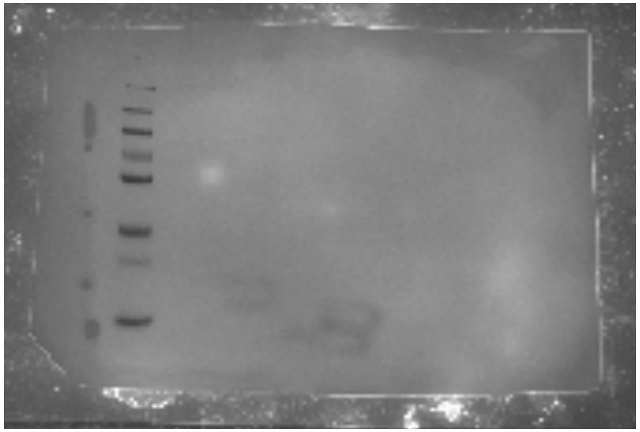

Uncropped blot for Precipitation Control (Figure 5A and Figure 5B)

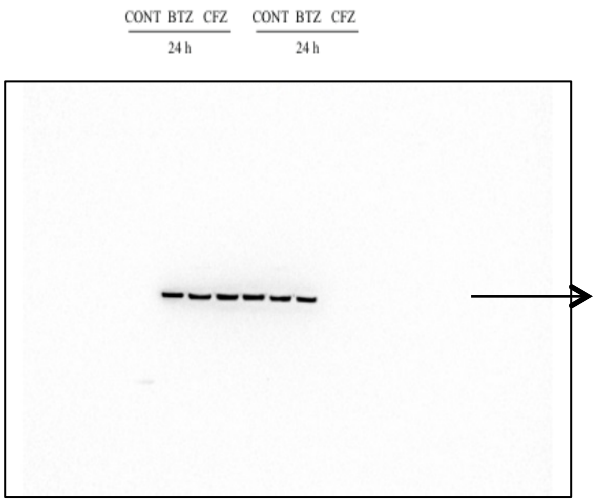

B-actin 42 kDa

Abcam Anti-beta Actin Antibody (clone ab8227)

Uncropped blot for Precipitation Control (Figure 5A and Figure 5B)

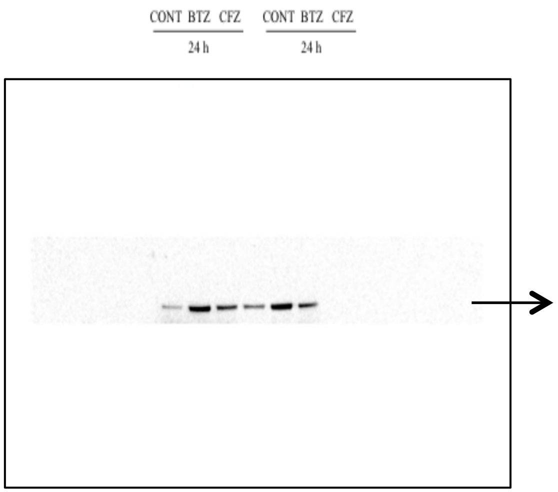

HSP70 70 kDa

Thermo Scientific Inc. Anti-HSP70 Antibody (clone 5A5)

Membrane for Precipitation Controls (Figure 5A and Figure 5B)

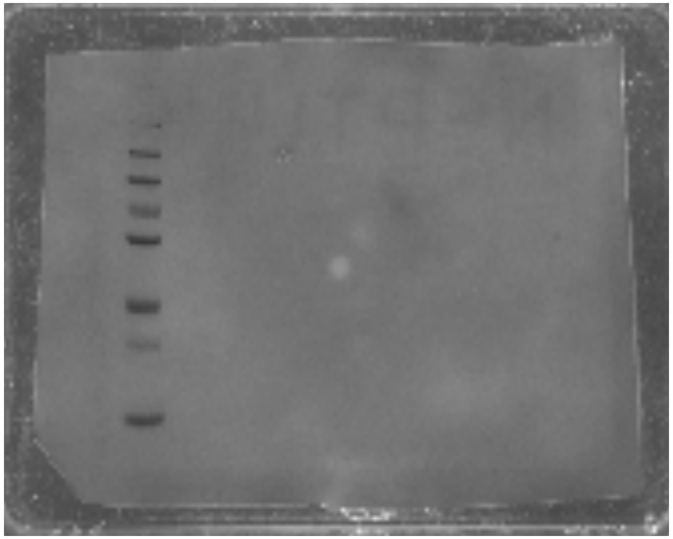

Membrane for K48-linked ubiquitinated proteins (Figure 6B)

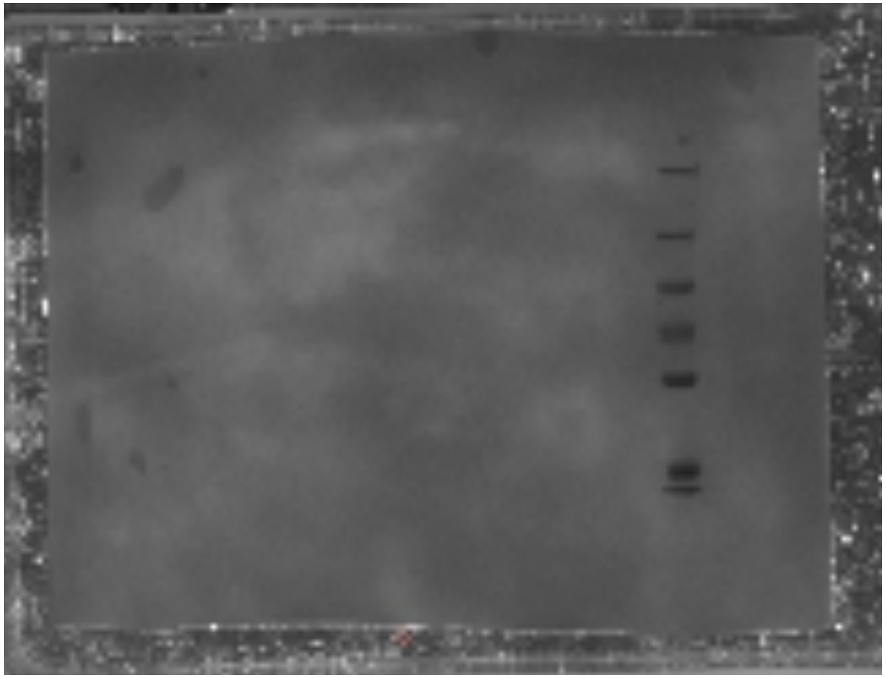

Membrane for Protein Carbonylation (Figure 6A)

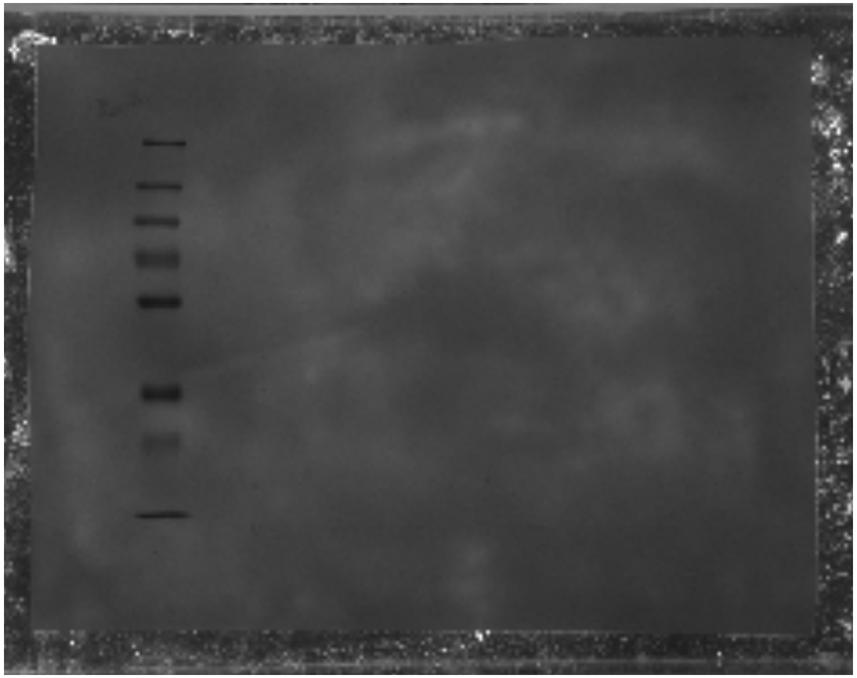

Uncropped blot for HSP27 (Figure S5A)

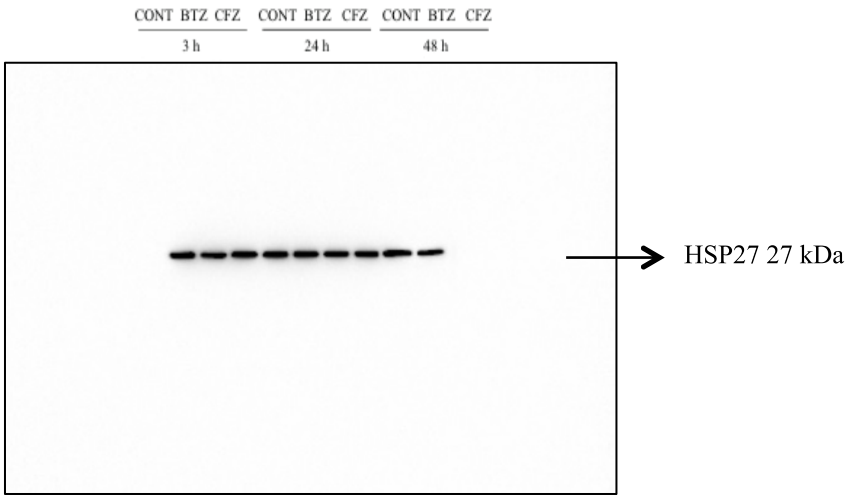

CST Anti-HSP27 Antibody (clone G31)

Uncropped blot for HSP60 and GAPDH (Figure S5C)

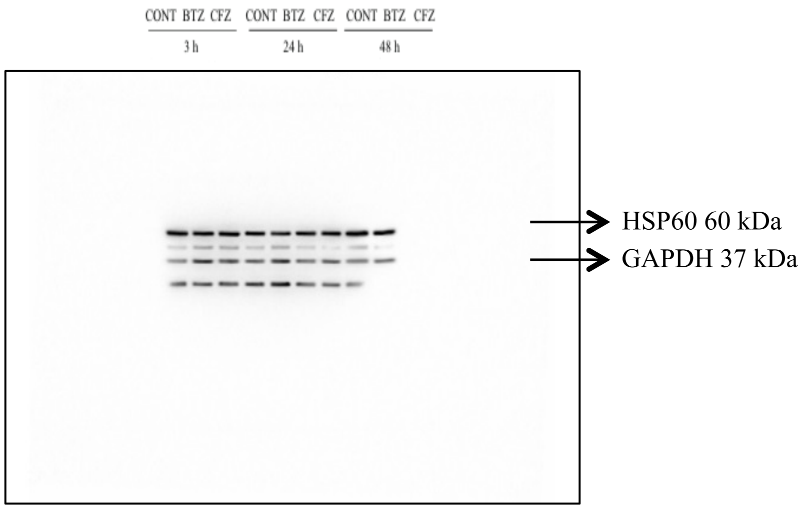

CST Anti-HSP60 Antibody (clone D307)

Membrane for HSP27, HSP60 and GAPDH (Figure S5A and Figure S5C)

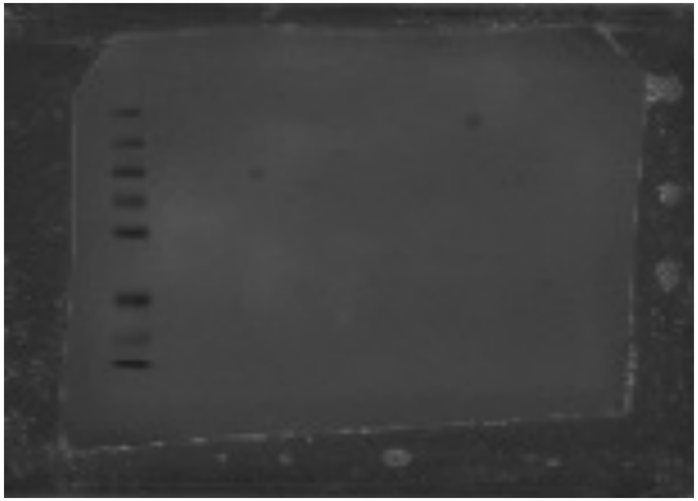

Uncropped blot for HSP40 (Figure S5C)

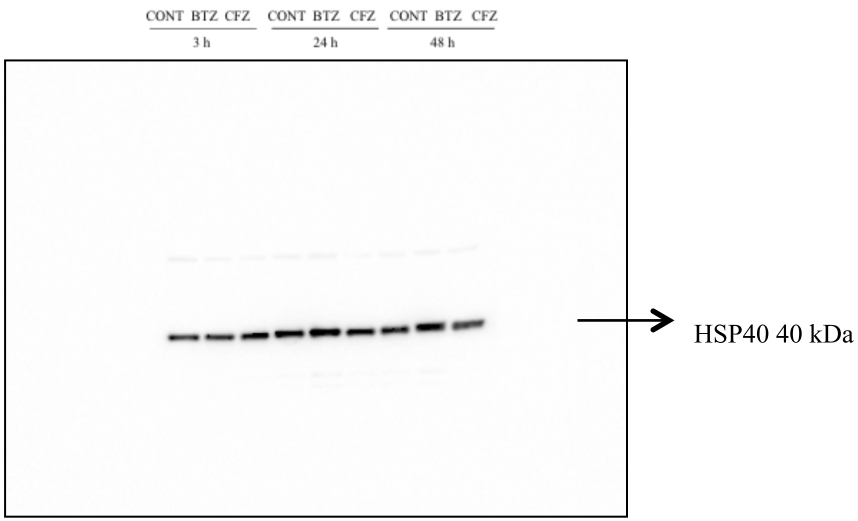

CST Anti-HSP40 Antibody (clone C64B4)

Uncropped blot for GAPDH (Figure 5SC)

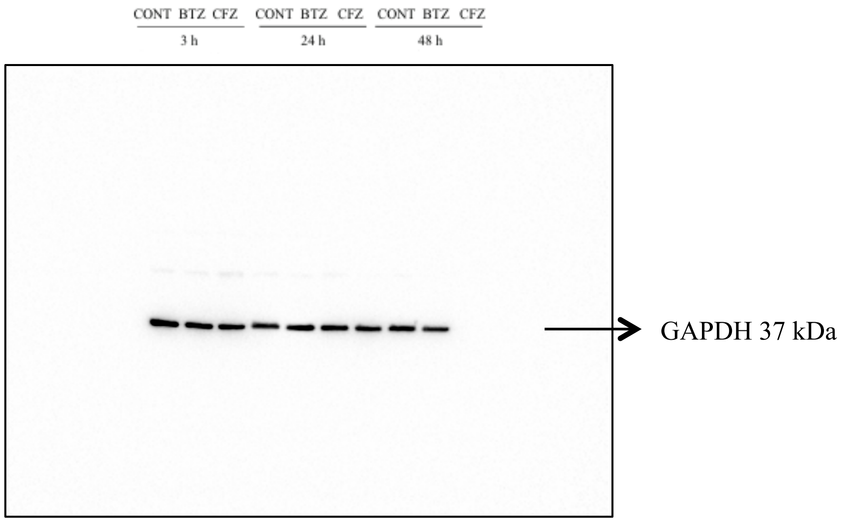

Abcam Anti-GAPDH antibody (clone 8245)

Membrane for HSP40 and GAPDH (S5C)

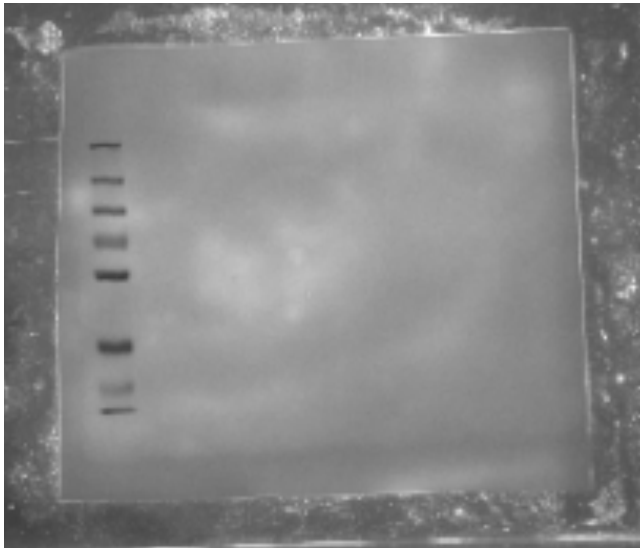

H9c2 CARDIOMYOCYTES

Uncropped blot for Nestin (Figure S7A)

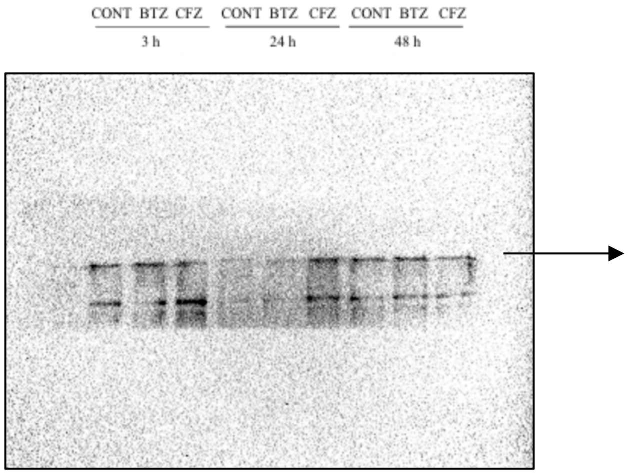

Uncropped blot for Vimentin (Figure S7B)

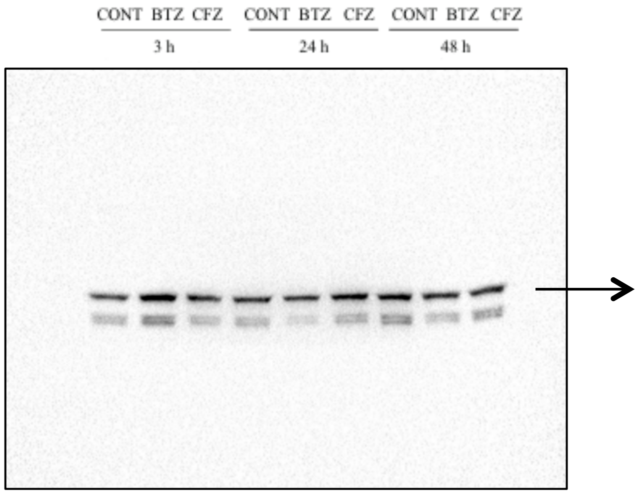

Uncropped blot for Transgelin-2 (Figure S8B)

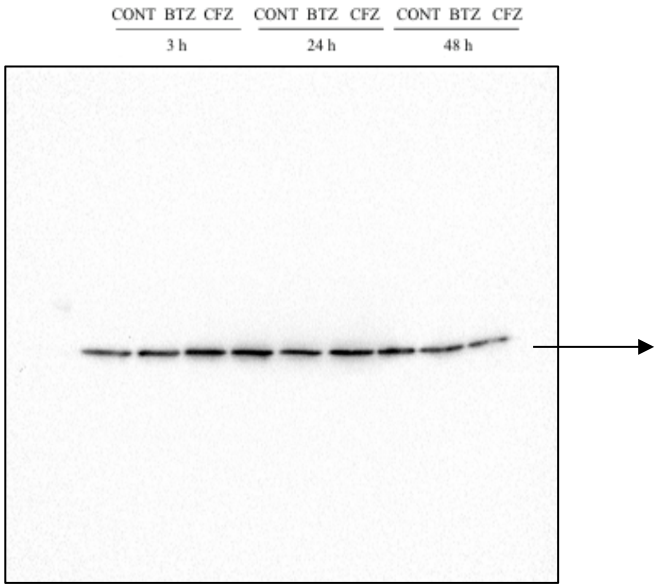

Uncropped blot for Coronin 1C (Figure S8C)

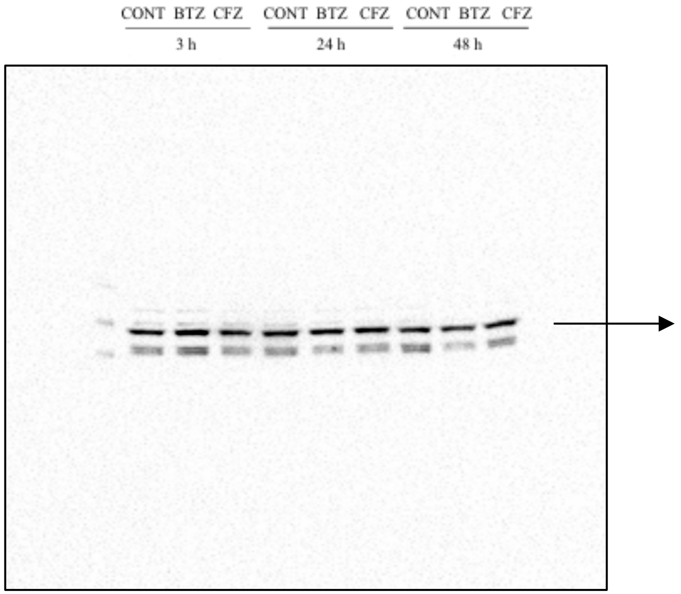

Uncropped blot for HSP32 (Figure S9A)

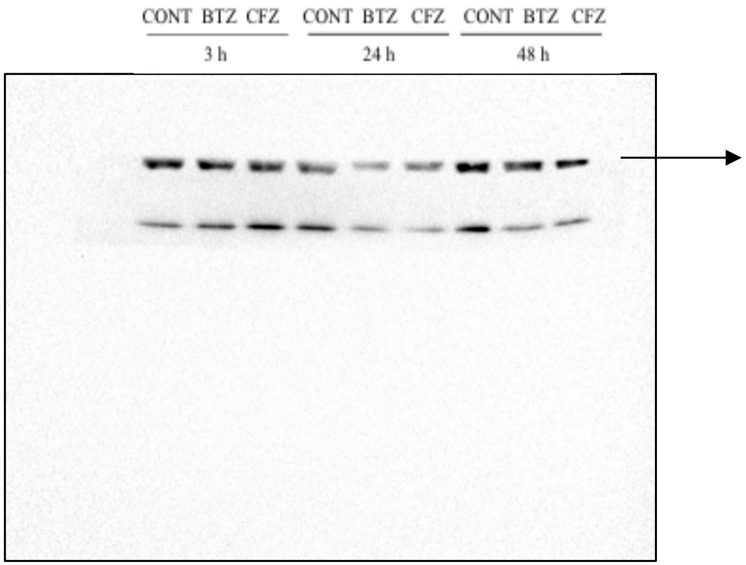

Uncropped blot for GAPDH (Figure S7A, S7B, S8B, S8C and S9A

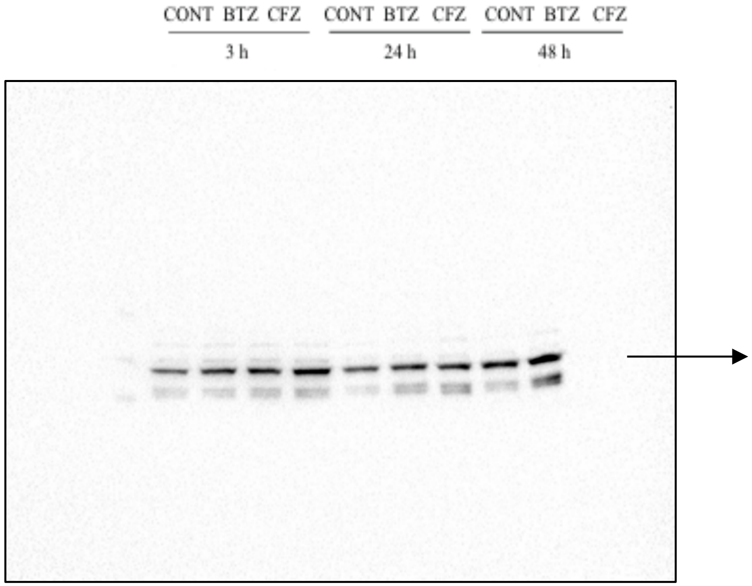

Membrane for Nestin, Vimentin, Transgelin-2, Coronin 1C, HSP32 and GAPDH (Figure S7A, S7B, S8B, S8C and S9A)

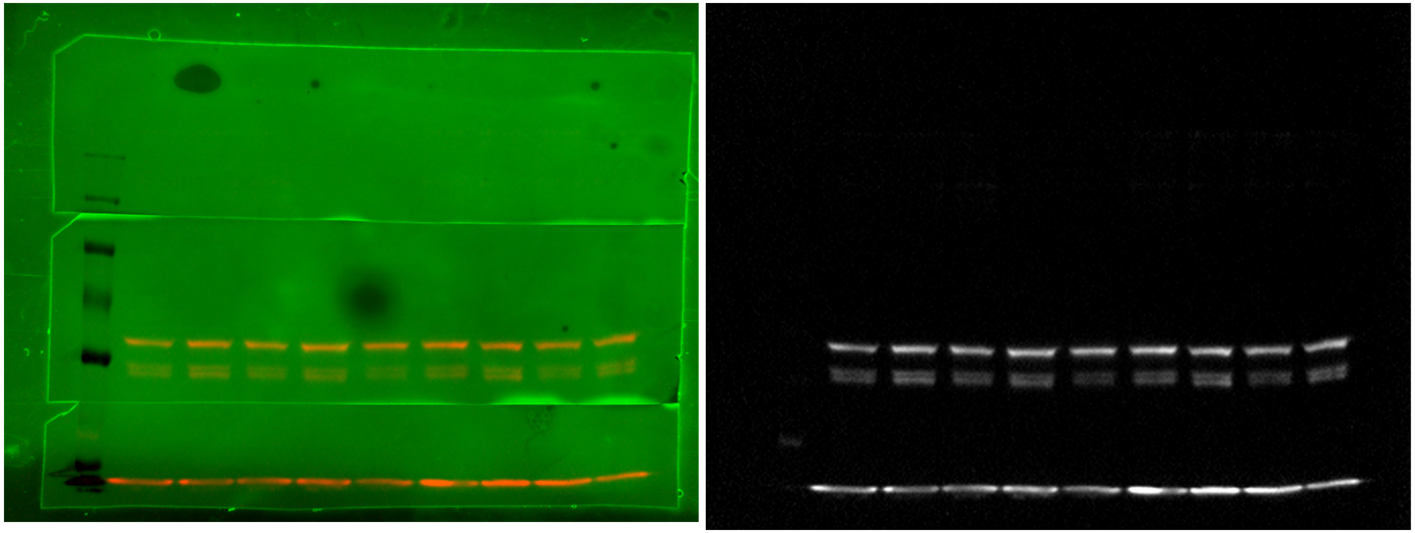

Uncropped blot for ARP2 (Figure S8A)

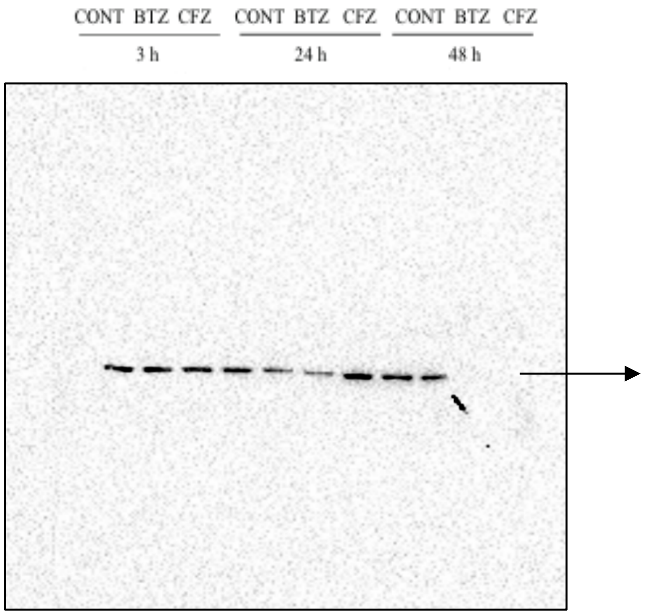

Uncropped blot for GRP94 (Figure S10B)

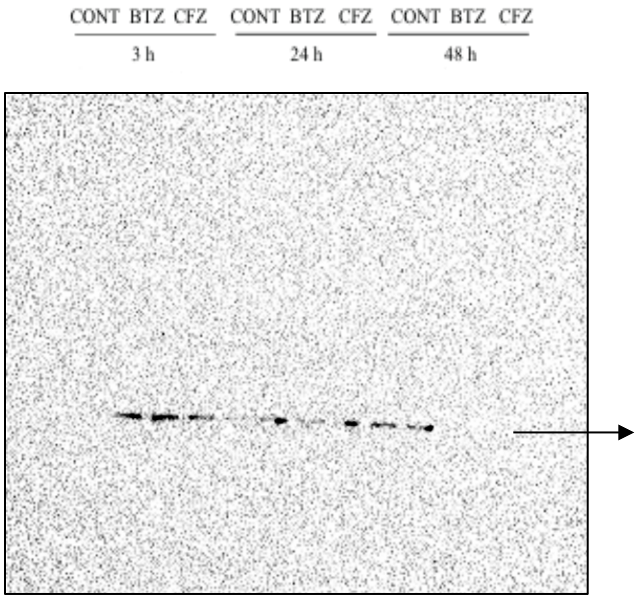

Uncropped blot for GAPDH (Figure S8A and S10B)

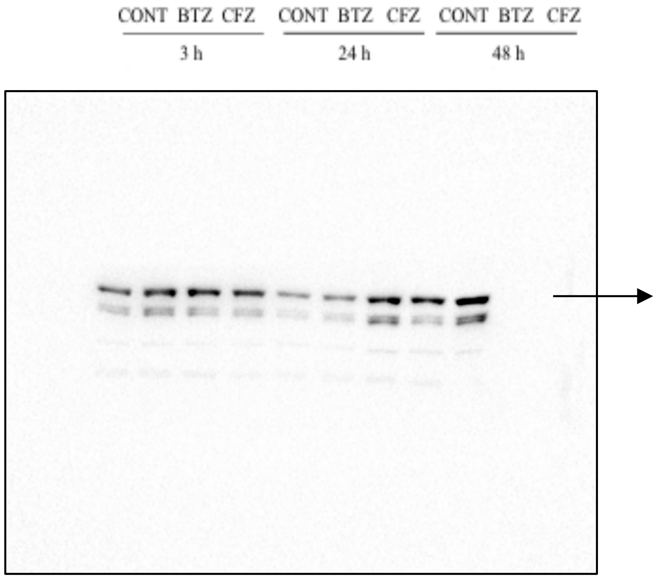

Membrane for ARP2, GRP94 and GAPDH (Figure S8A, S10B)

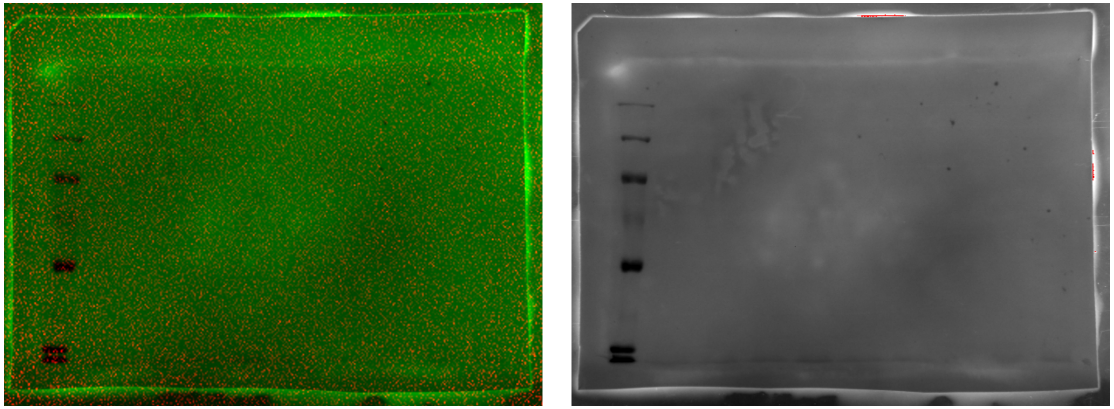

Uncropped blot for HSP47 (Figure S9B)

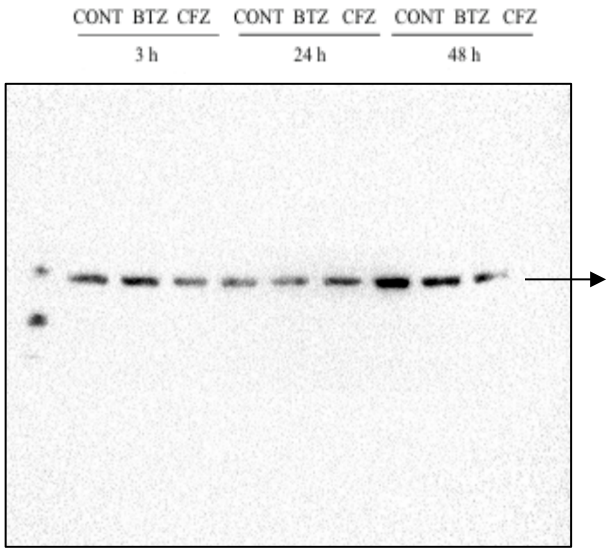

Uncropped blot for HSP70 (Figure S9E)

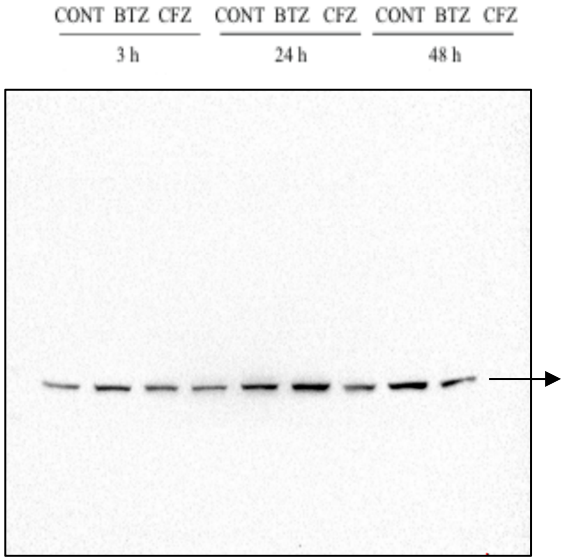

Uncropped blot for GRP78 (Figure S10A)

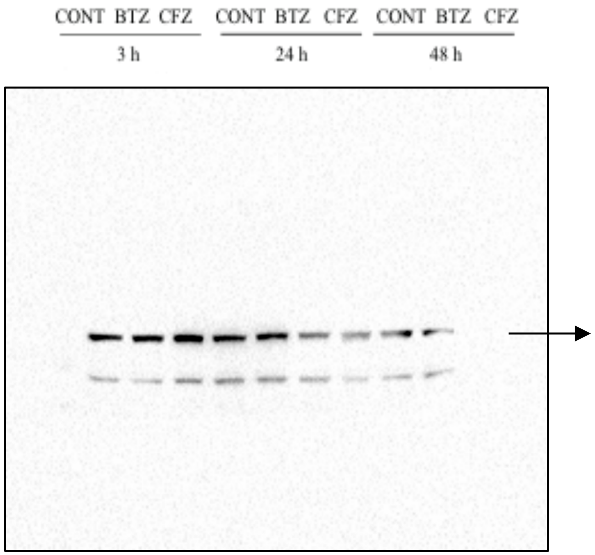

Uncropped blot for GAPDH (Figure S9B, S9E and S10A)

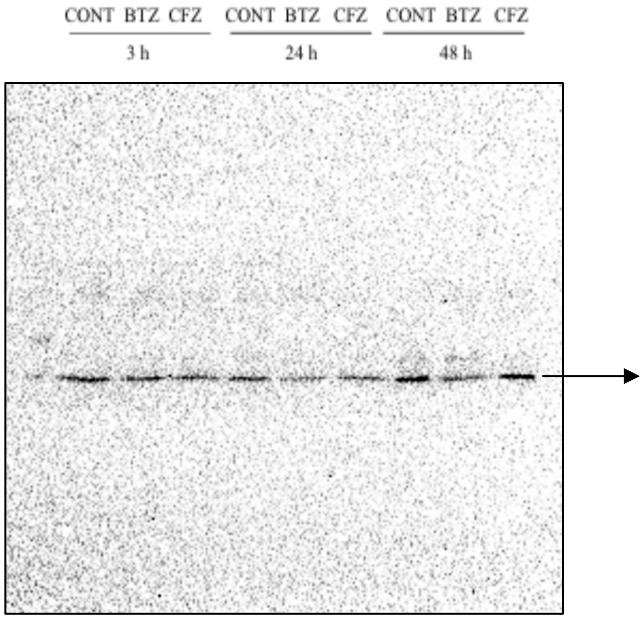

Uncropped Membrane for HSP47, HSP 70, GRP 78 and GAPDH (Figure S9B, S9E and S10A)

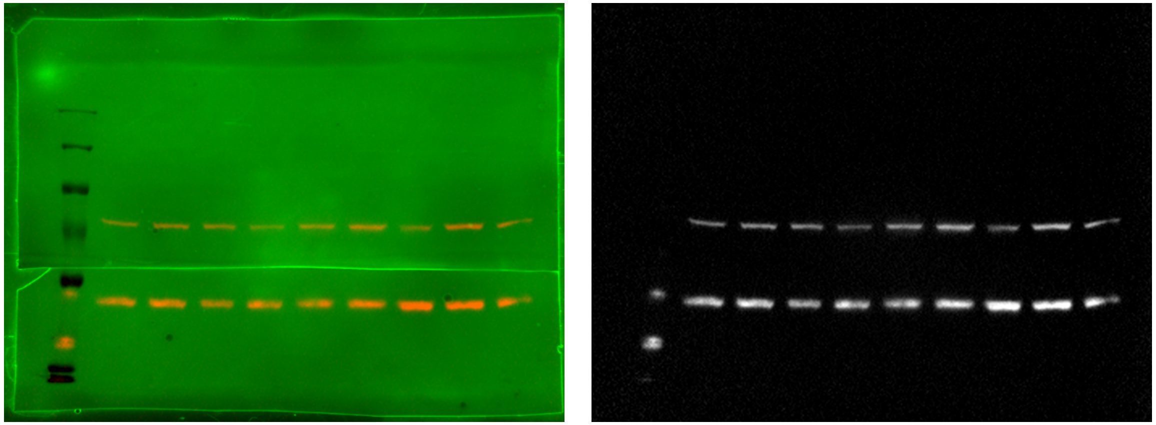

Uncropped blot for Protein Carbonylation (Figure S12A)

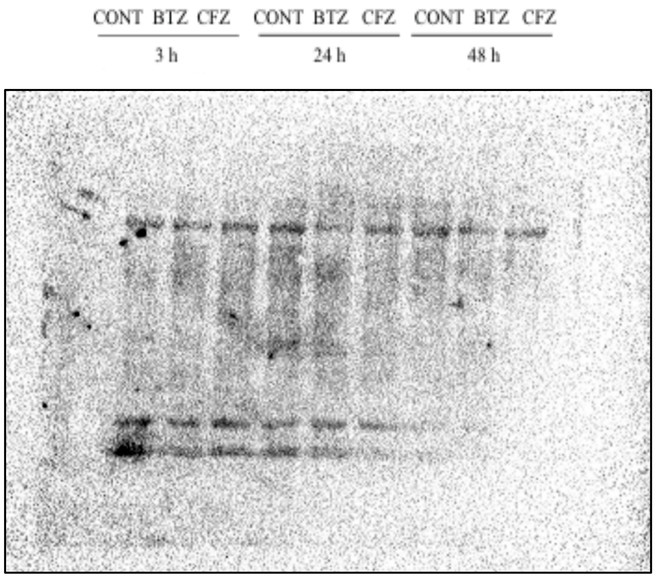

Membrane for Protein Carbonylation (Figure S12A)

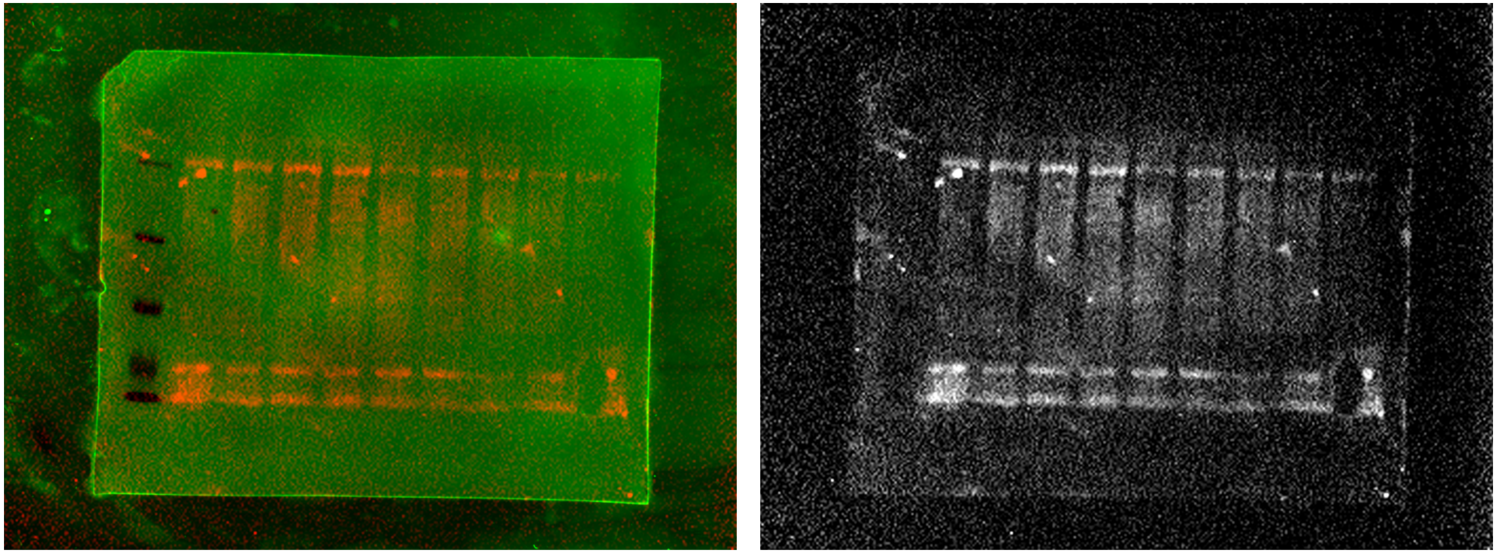

Uncropped blot for K48-linked ubiquitinated proteins (Figure S12B)

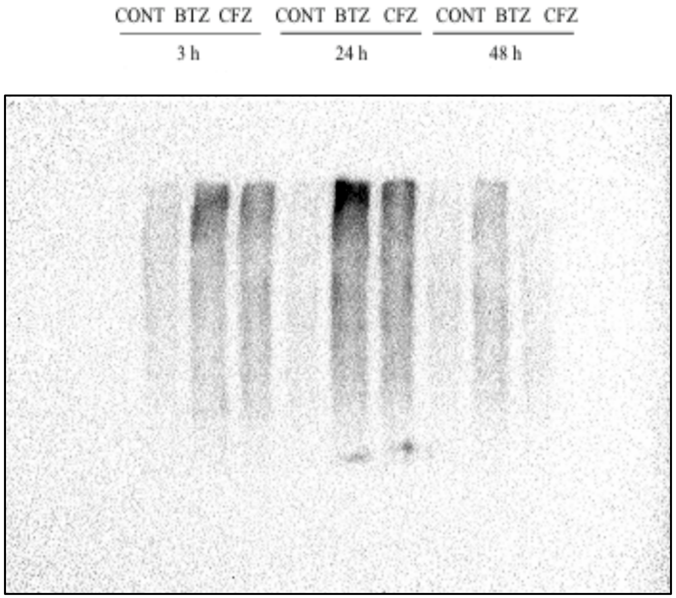

Membrane for K48-linked ubiquitinated proteins (Figure S12B)

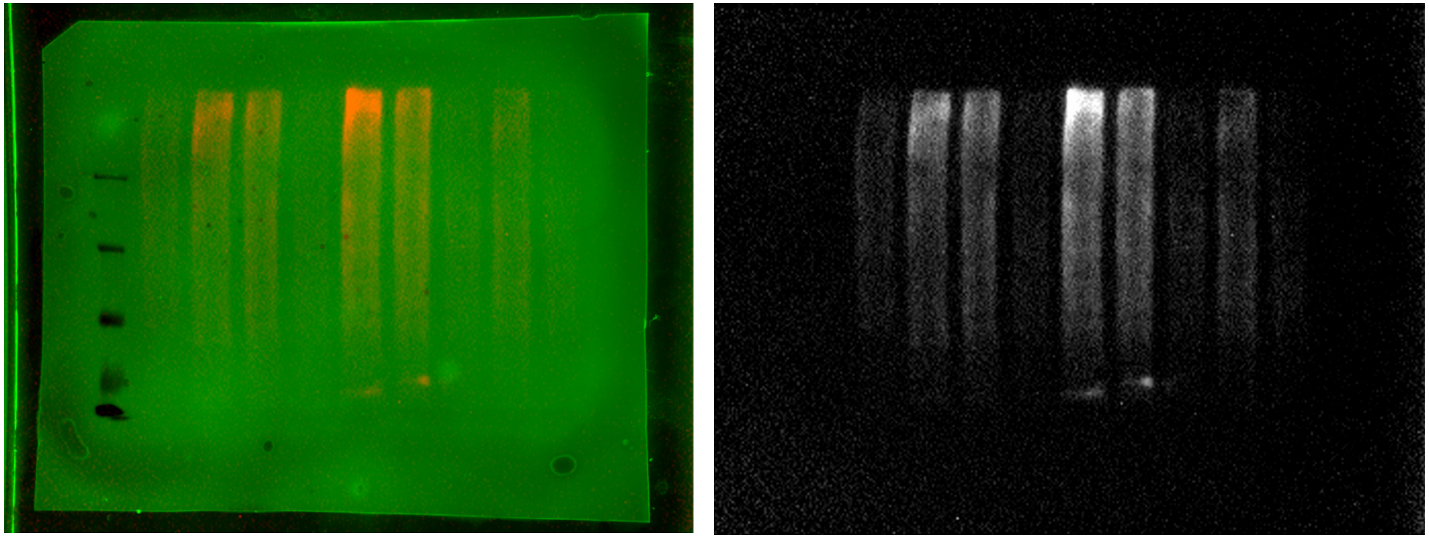

BALB/3T3 Cells

Uncropped blot for Nestin (Figure S7C)

CONT BTZ CFZ CONT BTZ CFZ CONT BTZ CFZ

3 h 24 h 48 h

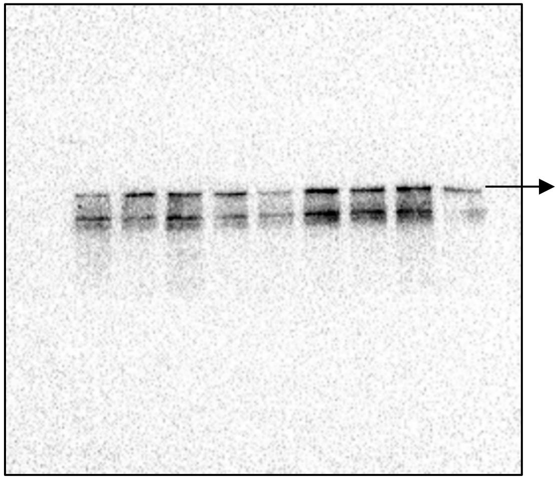

Uncropped blot for Coronin 1C (Figure S8D)

CONT BTZ CFZ CONT BTZ CFZ CONT BTZ CFZ

3 h 24 h 48 h

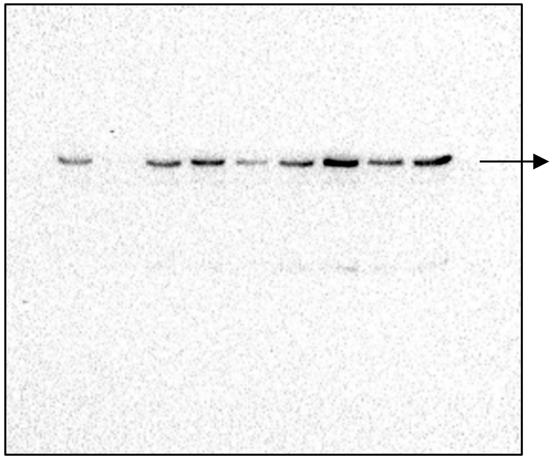

Uncropped blot for GRP78 (Figure S10C)

CONT BTZ CFZ CONT BTZ CFZ CONT BTZ CFZ

3 h 24 h 48 h

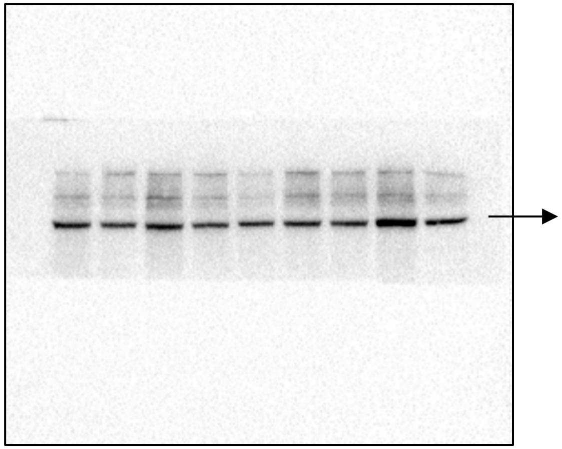

Uncropped blot for GAPDH (Figure S7C, S8C and S10C)

CONT BTZ CFZ CONT BTZ CFZ CONT BTZ CFZ

3 h 24 h 48 h

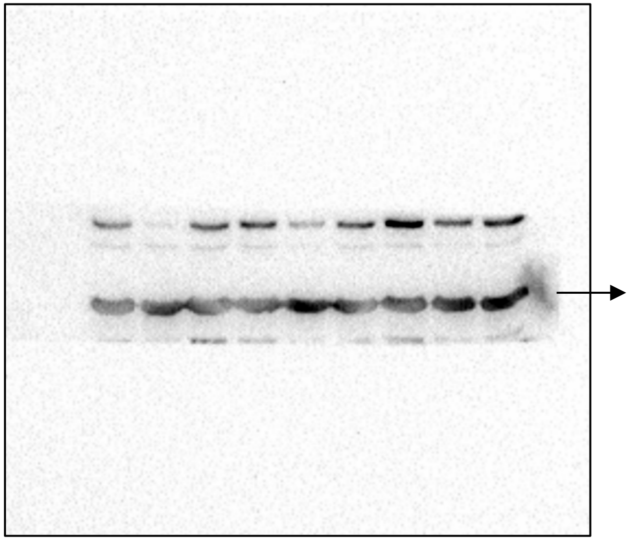

Membrane for Nestin, Coronin 1C, GRP78 and GAPDH (Figure S7C, S8D and S10C)

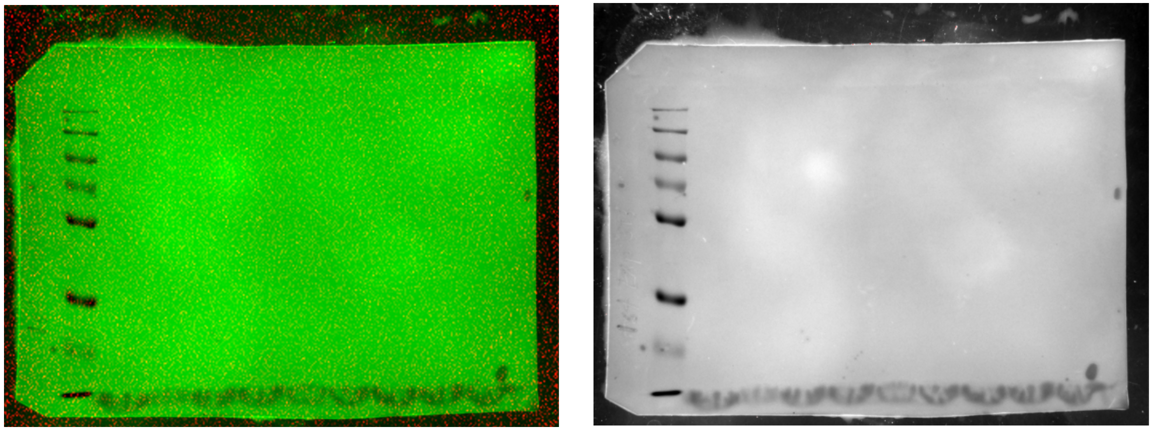

Uncropped blot for HSP47 (Figure S9D)

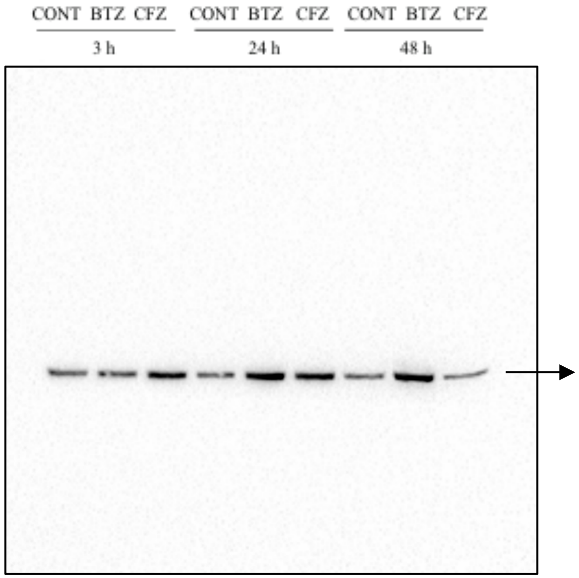

Uncropped blot for HSP70 (Figure S9E)

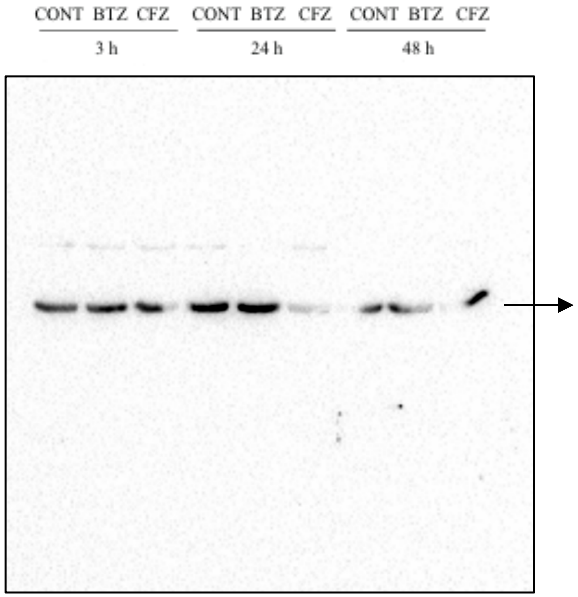

Uncropped blot for GRP94 (Figure S10D)

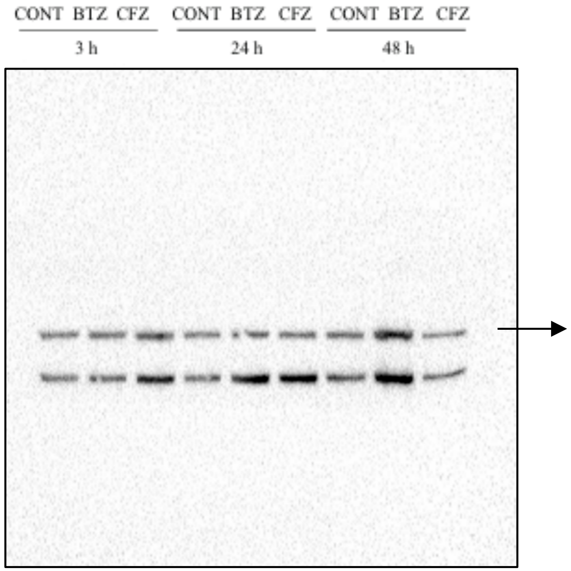

Uncropped blot for GAPDH (Figure S9D, S9E, S10C and S10D)

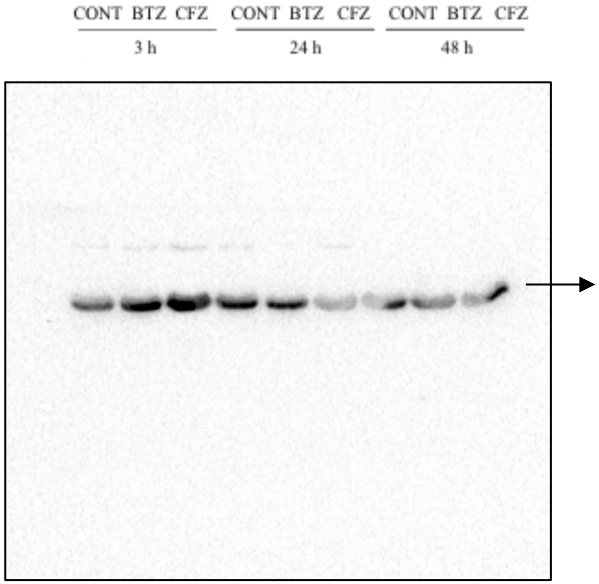

Membrane for HSp47, HSP70, GRP78, GRP94 and GAPDH (Figure S9D, S9E, S10C and S10D)

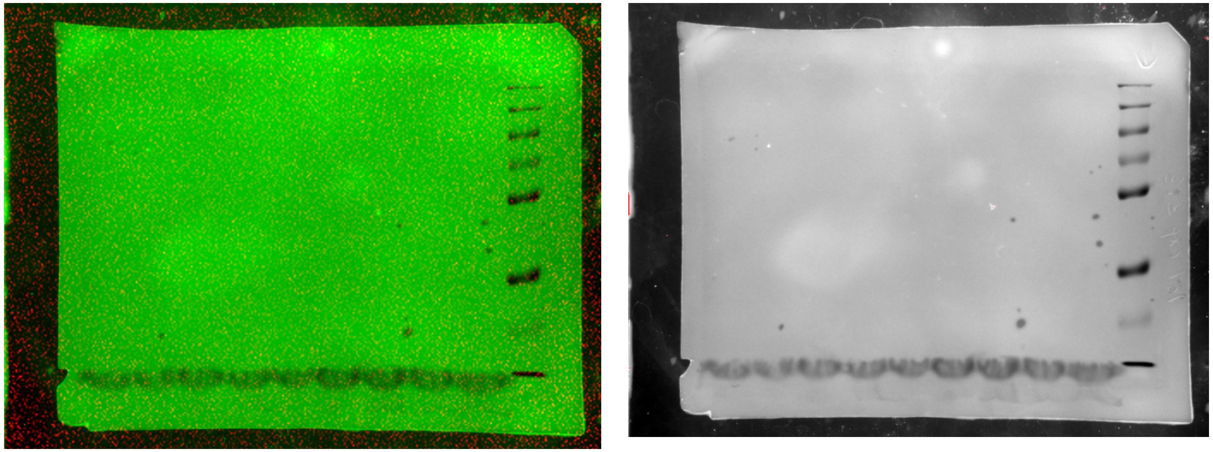

Uncropped blot for Vimentin (Figure S7D)

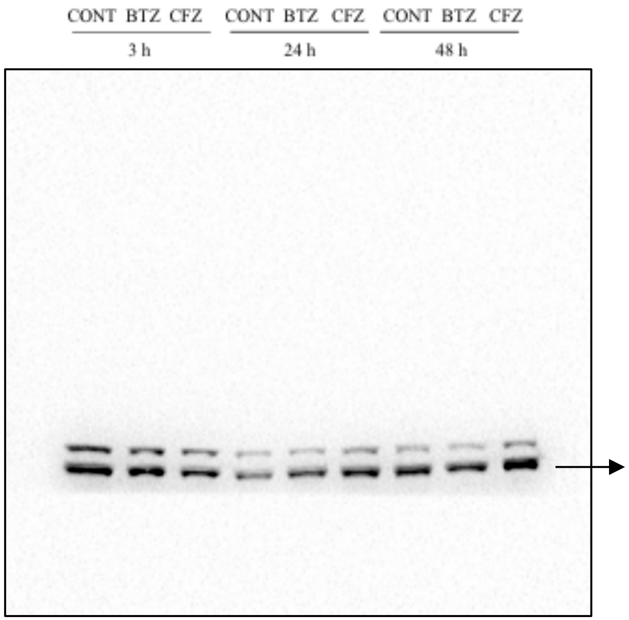

Uncropped blot for GAPDH (Figure S7D)

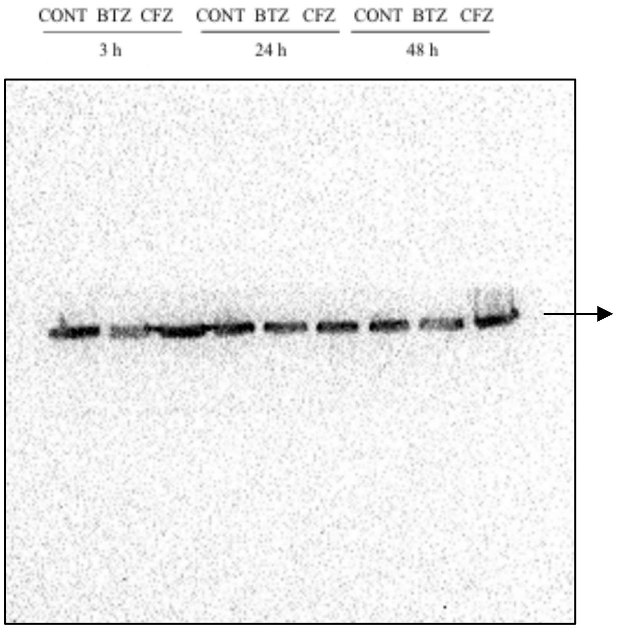

Membrane for Vimentin and GAPDH (Figure S7D)

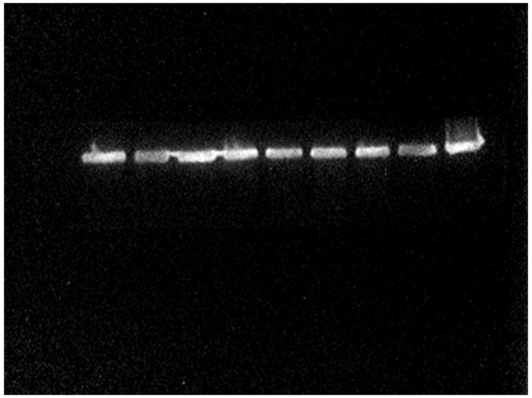

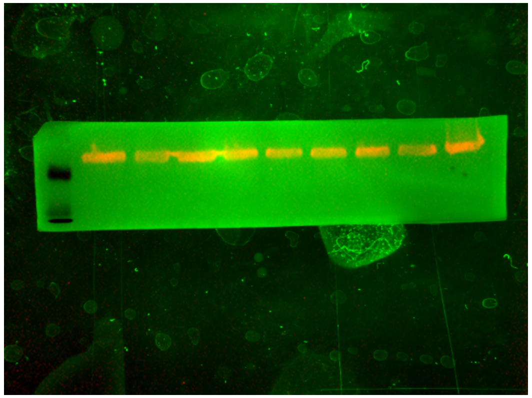

Uncropped blot for Protein Carbonylation (Figure S11A)

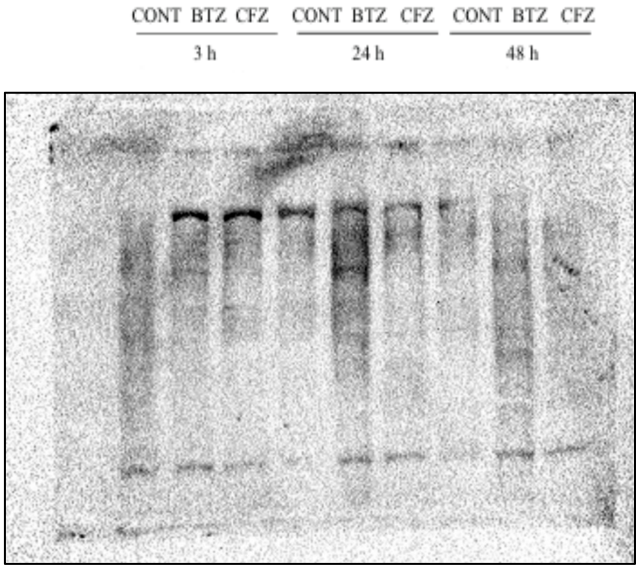

Membrane for Protein Carbonylation (Figure S11A)

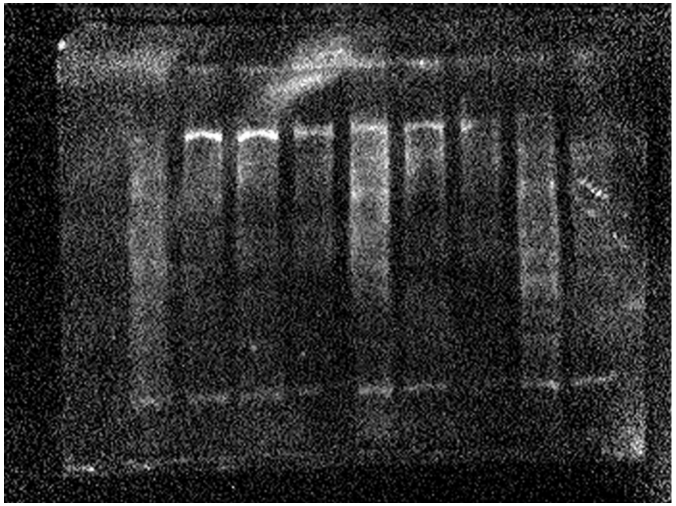

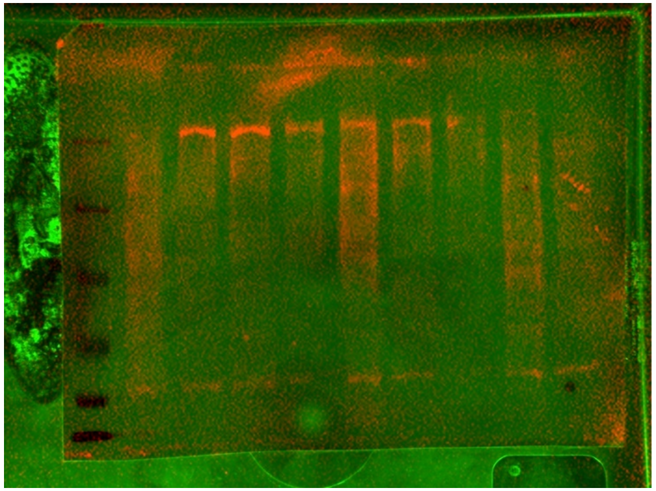

Uncropped blot for K48-linked ubiquitinated proteins (Figure S11B)

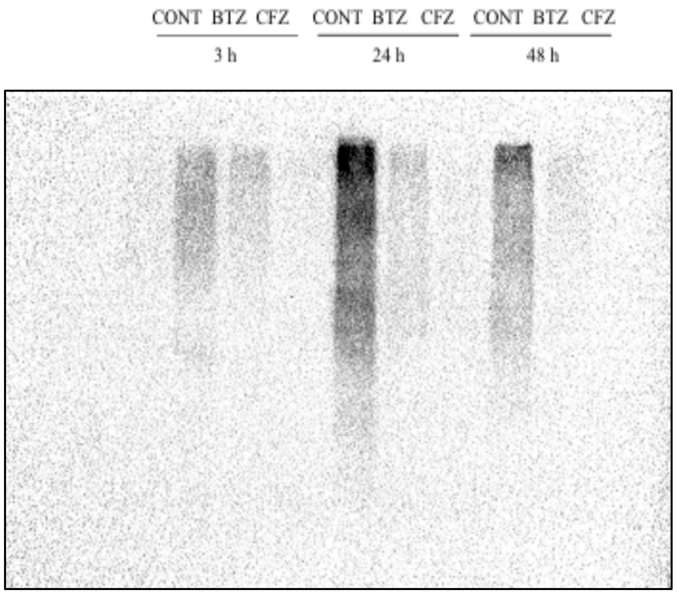

Membrane for K48-linked ubiquitinated proteins (Figure S11B)

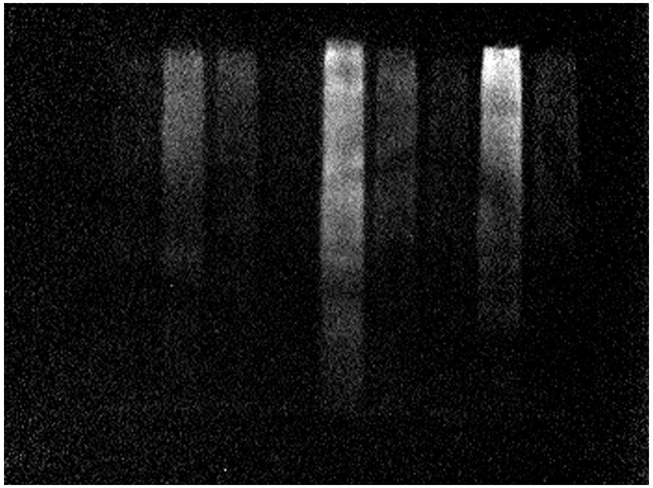

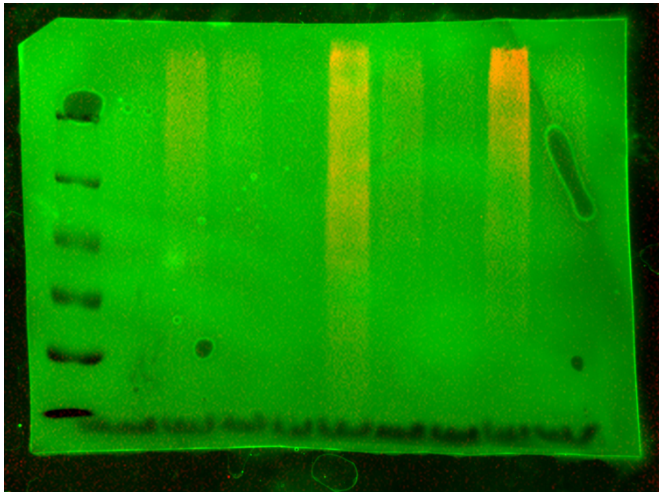

Supplement: Supplementary file 1 — Dataset 1 [file 41598_2018_34507_MOESM1_ESM.pdf]
